# Exploration and Exploitation Zones in a Minimalist Swarm Optimiser

**DOI:** 10.3390/e23080977

**Published:** 2021-07-29

**Authors:** Mohammad Majid al-Rifaie

**Affiliations:** School of Computing & Mathematical Sciences, University of Greenwich, Park Row, London SE10 9LS, UK; m.alrifaie@gre.ac.uk

**Keywords:** exploration, exploitation, diversity, zone analysis, dispersive flies optimisation, DFO

## Abstract

The trade off between exploration and exploitation is one of the key challenges in evolutionary and swarm optimisers which are led by guided and stochastic search. This work investigates the exploration and exploitation balance in a minimalist swarm optimiser in order to offer insights into the population’s behaviour. The minimalist and vector-stripped nature of the algorithm—dispersive flies optimisation or DFO—reduces the challenges of understanding particles’ oscillation around constantly changing centres, their influence on one another, and their trajectory. The aim is to examine the population’s dimensional behaviour in each iteration and each defined exploration-exploitation zone, and to subsequently offer improvements to the working of the optimiser. The derived variants, titled unified DFO or uDFO, are successfully applied to an extensive set of test functions, as well as high-dimensional tomographic reconstruction, which is an important inverse problem in medical and industrial imaging.

## 1. Introduction

Information exchange and communication between particles in swarm intelligence manifest themselves in a variety of forms, including the use of different update equations and strategies; deploying extra vectors in addition to the particles’ current positions; and dealing with tunable parameters. Ultimately, the goal of the optimisers is to achieve a balance between global exploration of the search space and local exploitation of potentially suitable areas in order to guide the optimisation process [1,2].

The motivation for studying dispersive flies optimisation, or DFO [3], is the algorithm’s minimalist update equation and its sole reliance on particles’ positions at time *t* to generate the positions at time t+1, therefore not using additional vectors. This characteristic [4] is in contrast to several other population-based algorithms and their variants which, besides using position vectors, use a subset of the following: velocities and memories (personal best and global best) in particle swarm optimisation (PSO) [5], mutant and trial vectors in differential evolution (DE) [6], pheromone and heuristic vectors in Ant Colony Optimisation (ACO) [7], and so forth. Besides only using position vectors in any given iteration (similar to some evolution strategies, such as CMA-ES [8]), the only tunable parameter in DFO, other than population size, is the restart threshold, Δ, which controls the component-wise restart in each dimension. This is again contrary to many well-known swarm and evolutionary algorithms dealing with several (theoretically- or empirically-driven) tunable parameters, such as: learning factors, inertia weight in PSO, crossover or mutation rates, tournament and elite sizes, constricting factor in DE and/or Genetic Algorithms (GA) [9], heuristic strength, greediness, pheromone decay rate in ACO, impact of distance on attractiveness, scaling factor and speed of convergence in Firefly algorithm (FF) [10], and so on. It is worthwhile to note that DFO is not the only minimalist algorithm, and there have been several attempts to present ‘simpler’, more compact algorithms to better understand the dynamic of population’s behaviour, as well as the significance of various communication strategies, but often still with more vectors and parameters, and often at the expense of performance. Perhaps one of the most notable minimalist swarm algorithm is barebones particle swarms [11]. Another barebones algorithm is barebones differential evolution [12], which is a hybrid of the barebones particle swarm optimiser and differential evolution, aiming to reduce the number of parameters, albeit with more than only the position vector. It is well understood that swarm intelligence techniques are dependant on the tuning of their parameters. This ultimately results in the need to adjust a growing number of parameters which becomes increasingly complex.

This paper aims at identifying and investigating knowledge-based exploration and exploitation zones in a minimalist, vector-stripped algorithm; therefore, using the analysis to propose ways to measure exploration and exploitation probabilities, with the ultimate goal of controlling the behaviour of the population by suggesting dimensionally-dependent exploration-exploitation balance without degrading the algorithm performance. Furthermore, the paper highlights the limitations and challenges of the proposed methods, which are also applied to tomographic reconstruction, where images are reconstructed using tomography.

In this work, the swarm optimiser is first presented in Section 2, followed by the analysis in Section 3, which subsequently leads to proposing adaptable exploration-exploitation mechanisms. Finally, in Section 4, the experiment results on a comprehensive set of benchmarks are presented.

## 2. Background

Dispersive flies optimisation (DFO) belongs to the broad family of population-based, swarm intelligence optimisers, which has been applied to various areas, including medical imaging [13], solving diophantine equations [14], PID speed control of DC motor [15], optimising machine learning algorithms [16], training deep neural networks [17], computer vision and quantifying symmetrical complexities [18,19], beer organoleptic optimisation [20], and analysis of autopoiesis in computational creativity [21].

In this algorithm, components of the position vectors are independently updated in each iteration, taking into account: the current particle’s position; the current particle’s best neighbouring individual (consider ring topology, where particles have left and right neighbours); and the best particle in the swarm. The update equation is

(1) $$\begin{array}{ccc} x_{id}^{t+1} & = & x_{i_{n}d}^{t}+u\left(x_{sd}^{t}-x_{id}^{t}\right), \end{array}$$

where

- $x_{id}^{t}$: position of *i*th particle in *d*th dimension at time step *t*;
- $x_{i_{n}d}^{t}$: position of ${\vec{x}}_{i}^{t}$’s best *neighbouring* individual (in ring topology) in *d*th dimension at time step *t*;
- $x_{sd}^{t}$: position of the *swarm*’s best individual in the *d*th dimension at time step *t*;
- $u∼U\left(0,1\right)$: generated afresh for each individual and each dimension update.

As a diversity-promotion mechanism, individual components of the population’s position vectors are reset if a random number generated from a uniform distribution on the unit interval $U\left(0,1\right)$ is less than the disturbance or *restart threshold*, Δ. This ensures a restart to the otherwise permanent stagnation over a likely local minima. In this method, which is summarised in Algorithm 1, each member of the population is assumed to have two neighbours (i.e., ring topology) and particles are not clamped to bounds, therefore, when out-of-bounds, are left unevaluated. The source code for standard DFO is available on http://github.com/mohmaj/DFO, accessed on 26 July 2021.
**Algorithm 1** Dispersive flies optimisation (DFO)
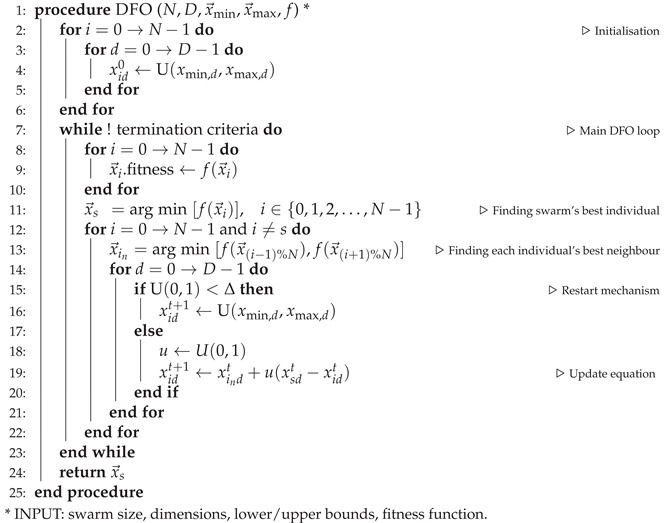


As a population-based continuous optimiser, DFO bears several similarities with other swarm and evolutionary algorithms. Stemming from its barebones and vector-stripped nature, DFO allows for further analysis while demonstrating competitive performance, despite being bare of “accessories”. As stated, DFO’s update mechanism relies solely on the position vectors at time *t* to produce the position vectors for time t+1, without storing extra vectors, and, in terms of tunable parameters, other than population size, DFO uses one extra parameter for adjusting the global diversity of the population. To provide more context and before the analysis, a number of well-known algorithms, along with their tunable (and/or theoretically-driven) parameters, are provided.

For instance, PSO, in many of the proposed variants, commonly uses the following parameters: population size; $c_{1}$, controlling the impact of cognitive component; $c_{2}$, controlling the impact of social component; χ or *w*, depending on the update equation. In addition to the position of particle *i*, ${\vec{x}}_{i}$, each particle has an associated velocity, ${\vec{v}}_{i}$, and memory, ${\vec{p}}_{i}$, vectors. Other variants of PSO, including barebone PSOs were also introduced to simplify the algorithm, with the ultimate goal of offering insight into the algorithm’s underlying behaviour. In one such case, one of the inventor of PSO, Kennedy, describes the process as “*strip[ping] away some traditional features*” with the hope of *revealing the mysteries of the algorithm* [11]. In this particular model, the velocity vectors are removed, while the algorithm still benefits from having memories, a work that was carried out to shed light on the behaviour of the algorithm. Other contributions have tried to further explore the simplified version and enhance its performance, demonstrating the capability of the simplified version in contrast with the original models [22,23,24,25].

Other than PSO, parameters and adjustable configurations of other well-known algorithms include those of GAs [9]: population size, $p_{c}$: crossover rate, $p_{m}$: mutation rate, tournament size, elite size; DE [6,26]: population size, $p_{c}$: crossover rate, equations used to calculate the mutation vector (e.g., the most notable ones are: DE/rand/1, DE/rand-to-best/1, DE/best/1, DE/best/2, DE/rand/2), F: constricting factor; ACO [7]: *m*: number of ants (population size), β: heuristic strength, α: greediness, ρ: pheromone decay rate; Firefly algorithm or FA [10]: population size, *m*: impact of distance on attractiveness, α which could be replaced with $\alpha S_{k}$ in cases where scales vary significantly in different dimensions, *d*. Thus, given *d* dimensions (k=1,…,d), adding *d* extra parameters, γ determining the speed of convergence, in theory, γ∈[0,∞), with γ=0 maintaining a constant attractiveness of $\beta =\beta_{0}$.

Looking at the update equations of DFO, PSO and DE’s mutant vector (DE1: DE/rand-to-best/1 and DE2: DE/best/1), certain similarities can be identified:

(2) $$\begin{array}{ccc} \mathrm{PSO} & : & v_{id}^{t+1}=wv_{id}^{t}+c_{1}r_{1}\left(p_{id}-x_{id}^{t}\right)+c_{2}r_{2}\left(g_{d}-x_{id}^{t}\right) \end{array}$$

(3) $$\begin{array}{ccc} & : & x_{id}^{t+1}=v_{id}^{t+1}+x_{id}^{t} \end{array}$$

(4) $$\begin{array}{ccc} & : & x_{id}^{t+1}=f_{\mathrm{PSO}}\left(v_{id}^{t+1},\;p_{id},\;g_{d},\;x_{id}^{t}\right), \end{array}$$

(5) $$\begin{array}{ccc} \mathrm{DE}\;1 & : & v_{id}^{t+1}=x_{id}^{t}+F\left(x_{best,d}^{t}-x_{id}^{t}\right)+F\left(x_{r_{1}d}^{t}-x_{r_{2}d}^{t}\right)\\ & : & \ldots \end{array}$$

(6) $$\begin{array}{ccc} & : & x_{id}^{t+1}=f_{\mathrm{DE}1}\left(v_{id}^{t+1},\;u_{id}^{t+1},\;{\vec{x}}_{d}^{t}\right), \end{array}$$

(7) $$\begin{array}{ccc} \mathrm{DE}\;2 & : & v_{id}^{t+1}=x_{best,d}^{t}+F\left(x_{r_{1}d}^{t}-x_{r_{2}d}^{t}\right)\\ & : & \ldots \end{array}$$

(8) $$\begin{array}{ccc} & : & x_{id}^{t+1}=f_{\mathrm{DE}2}\left(v_{id}^{t+1},\;u_{id}^{t+1},\;{\vec{x}}_{d}^{t}\right), \end{array}$$

(9) $$\begin{array}{ccc} \mathrm{DFO} & : & x_{id}^{t+1}=x_{i_{n}d}^{t}+u\left(x_{sd}^{t}-x_{id}^{t}\right) \end{array}$$

(10) $$\begin{array}{ccc} & : & x_{id}^{t+1}=f_{\mathrm{DFO}}\left({\vec{x}}_{d}^{t}\right), \end{array}$$

where, for PSO, *w* is the inertia weight whose optimal value is problem dependent [27]; $v_{id}^{t}$ is the velocity of particle *i* in dimension *d* at time step *t*; $c_{1,2}$ are the learning factors (also referred to as acceleration constants) for personal best and neighbourhood best, respectively; $r_{1,2}$ are random numbers adding stochasticity to the algorithm, and they are drawn from a uniform distribution on the unit interval $U\left(0,1\right)$; $p_{id}$ is the personal best position of particle ${\vec{x}}_{i}$ in dimension *d*; $g_{d}$ is swarm best at dimension *d*; and $f_{\mathrm{PSO}}$ takes as input the variables needed at time *t* in order to return the particle’s component’s position at time t+1. For DE’s mutant vector (DE1: DE/rand-to-best/1 and DE2: DE/best/1), $v_{id}$ is *d*th gene of the *i*th chromosomes’s mutant vector ($\vec{v}$ in PSO and DE are different, albeit they carry the same name in the literature); $u_{id}$ is *d*th gene of the *i*th chromosomes’s trial vector; $r_{1}$ and $r_{2}$ are different from *i* and are distinct random integers drawn from the range $\left[1,N\right]$; and $x_{best,d}^{t}$ is the *d*th gene of the best chromosome at generation *t*; *F* is a positive control parameter for constricting the difference vectors.

In these update equations, similarities between PSO’s Equations (2)–(4) and DE1’s Equations (5) and (6) can be observed, including current and best positions, and the use of extra components to steer the update process (e.g., in PSO: velocity, $\vec{v}$, and memories, $\vec{p}$; and in DE1: mutant vector, $\vec{v}$, and trial vector, $\vec{u}$), as shown in Equations (4) and (6).

On the other hand, there are similarities between DE2 (DE/best/1) and DFO, as shown in Equations (7)–(10). In their update equations, the focus ($x_{best,d}$ and $x_{i_{n}d}$) is either the best chromosome in the population or the best neighbouring particle, and the spread is determined by taking into account two members of the population: in DE2’s instance, it uses the distances between two random chromosomes, and, in DFO’s case, the distance between the best particle and the current particle’s positions is calculated; both of these distances are then “controlled” (i.e., by *F* in DE2, and by u=U(0,1) in DFO). Furthermore, DFO’s use of evolutionary phases (i.e., mutation, crossover, and selection) can be demonstrated in the restart mechanism, update equation, and the elitism strategy, respectively, where particles’ current positions determine their next positions, i.e., ${\vec{X}}^{t+1}=f_{\mathrm{DFO}}\left({\vec{X}}^{t}\right)$, with $\vec{X}$ being a 2D matrix of particles positions.

Therefore, following on the above and to quote Kennedy [11]: “*The particle swarm algorithm has just enough moving parts to make it hard to understand*”, and this work builds on of its key motivation to analyse a minimalistic algorithm to:

- reduce the challenges of understanding particles oscillating around the constantly changing centres (in each iteration, independently),
- understand particles’ influence on one another (and their contribution to the swarm’s next iteration), and
- strip the parameters in the analysis to understand the trajectory of particles (moving between different regions in the feasible search space).

To address these challenges, the minimalist, vector-stripped features of the optimiser are used to provide an analysis of the population’s exploration-exploitation behaviour.

## 3. Exploration-Exploitation Zones Analysis

As shown in the update equation, Equation (1), for each particle, the search focus is $\vec{\mu }={\vec{x}}_{i_{n}}$, and the spread, $\vec{\sigma }={\vec{x}}_{s}-{\vec{x}}_{i}$, is the distance between the best particle in the swarm and the current particle. Therefore, the equation could be rewritten for each particle’s dimension as

(11) $$\begin{array}{c} x=\mu +u\sigma . \end{array}$$

The spatial location of particles and their proximity to the global optimum of a given function, informs the role played by μ and σ. Considering one dimension of a problem and for ease of read in the remaining of this section, *x* refers to $x_{i}^{t}$; $x^{\prime }$ refers to $x_{i}^{t+1}$; *g* refers to $x_{s}^{t}$; and *n* refers to $x_{i_{n}}^{t}$. Furthermore, *exploitation* refer to the approaching of *x* to *g* (i.e., $|g-x^{\prime }\left|<|g-x\right|$). By the same token, *exploration* refers to the increasing distance between *x* and *g* (i.e., $|g-x^{\prime }\left|>|g-x\right|$). This section presents the unified exploration-exploitation analysis where a number of zones are identified, and their roles in terms of exploration and exploitation are investigated and ultimately measured.

Consider *x* is to be uniform in [−L,R], while *g* and *n* are fixed. Given this, the areas highlighting exploitation can be plotted using *A* and *B* below:

$$\begin{array}{cc} g & =0,\\ n & =1,\\ A:u & =1-\frac{1}{\left|x\right|},\\ B:u & =\frac{1}{x}-1. \end{array}$$

**Figure 1:**
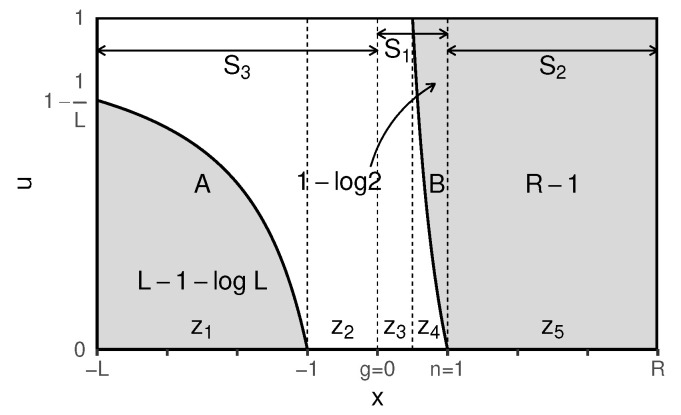
Unified exploitation probability, P(exploit) or *p*. The shaded areas in the graph represent exploitation, where particles in these areas at time *t* will be exploiting at time t+1.

To proceed, and as shown in Figure 1, the exploitation probabilities in the following four cases are presented individually:

1. R,L≥1,
2. R=L=1,
3. $L\in \left[0,1\right],R\in \left[\frac{1}{2},1\right]$,
4. $L\in \left[0,1\right],R\in \left[0,\frac{1}{2}\right]$.

$$\begin{array}{cc} R,L\geq 1: & \\ \; & P\left(\mathrm{exploit}\right)=\frac{L+R-1-\log2L}{L+R}\\ \; & \mathrm{put}\;L=R=x\\ \; & P\left(\mathrm{exploit}\right)=\frac{2x-1-\log2x}{2x}\\ \; & \lim_{x\to \infty }P\left(\mathrm{exploit}\right)=\lim_{x\to \infty }1-\frac{1}{2x}-\frac{\log2x}{2x}\\ \; & =1\\ R=L=1: & \\ \; & P\left(\mathrm{exploit}\right)=\frac{1-\log2}{2}\\ L\in \left[0,1\right],R\in \left[\frac{1}{2},1\right]:\\ \; & P\left(\mathrm{exploit}\right)=\frac{2R-1-\log2R}{L+R}\\ L\in \left[0,1\right],R\in \left[0,\frac{1}{2}\right]:\\ \; & P(\mathrm{exploit})=0. \end{array}$$

**Figure A2:**
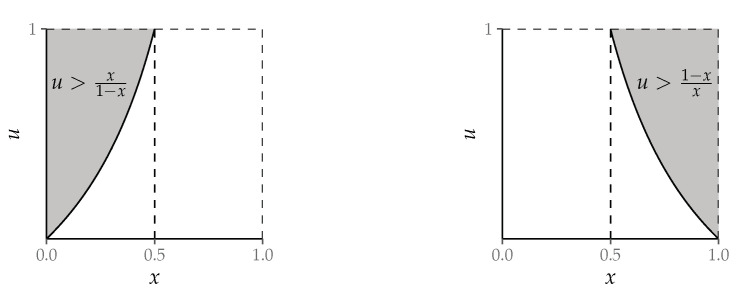
Exploitation probability in scenario 1. (**Left**) n≤x≤g; (**Right**) g≤x≤n.

The exploitation probability for instances when R=L=1 is derived from the first case (i.e., R,L≥1); the probability confirms the findings in the scenario-based analysis presented in Appendix A.1 for scenario 1 (see $S_{1}$ in Figure 1 or Figure A2), where *x* is between *g* and *n* (x∈[0,1]). This illustrates the link between the unified and the scenario-based analyses. The scenario-based analysis can be found in Appendix A, where the three scenarios, S${}_{1}$, S${}_{2}$, S${}_{3}$ are examined. Furthermore, the scenario-based analysis assumes a start from the initial state and is based on the position of *x* in relation to *n* and *g*. While the scenario-based analysis is independent of the feasible bounds to the search space, this aspect is taken into account in the unified exploitation analysis.

Based on the analysis, for {x∈R:−L≤x≤R} and given the tendency of L,R≥1 in the scaled space (influenced by the proximity of *g* and *n* over time), the unified exploitation probability, P(exploit) or *p*, is summarised as:

(12) $$\begin{array}{cc} p=P(\mathrm{exploit}) & =P(|x^{\prime }-g|<|x-g|)\\ & =\frac{L+R-1-\log2L}{L+R}. \end{array}$$

### 3.1. Self-Adaptive Variants

Based on the analysis, an immediate line of research is to measure the iteration-based, dimensional probabilities of exploitation (*p*) to facilitate diversity adjustment. The dimensional diversity mechanism can be facilitated through an adaptable restart threshold, $\Delta_{\mathrm{dynamic}}$ (as opposed to a pre-determined parameter value, Δ).

#### 3.1.1. Unified DFO (uDFO)

**Figure 2:**
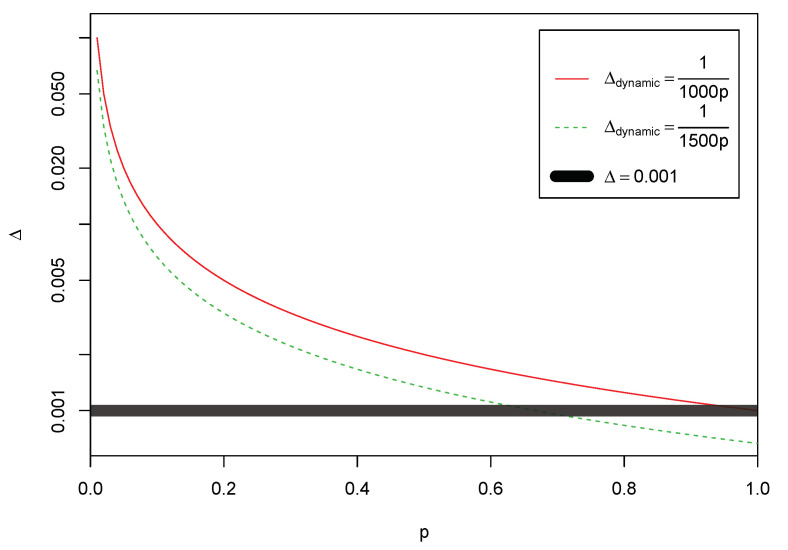
Component-wise restart threshold, based on *p*, with $\Delta_{\mathrm{dynamic}}=\left\{1/1000p,1/1500p\right\}$. The restart threshold of the original DFO (Δ=0.001) is illustrated in black.

In one such approach, the unified exploitation probability, *p*, is measured for each dimension and in each iteration. Using *p*, the component-wise restart is triggered when $r<\Delta_{\mathrm{dynamic}}$, where r=U(0,1), and $\Delta_{\mathrm{dynamic}}$ controls the restart mechanism dynamically. In order to take into account the previously reported empirical restart threshold of Δ=0.001 [3], in one set of experiments, $\Delta_{\mathrm{dynamic}}$ is set to 1/1000p, where $\Delta_{\mathrm{dynamic}}=\Delta$ when p=1, or higher when p<1. This approach has similarities with standard DFO at the high end of *p*. Alternatively, in the second approach, $\Delta_{\mathrm{dynamic}}=1/1500p$, where the previously derived empirical restart threshold is reached when $p=\bar{0.6}$, and higher when $p<\bar{0.6}$ (see Figure 2).

The adapted versions of the algorithm, which benefit from the unified exploitation probability, are termed *unified* DFO or uDFO. Using the proposed methods enabled the adaptive, dimension-dependant diversity to be present throughout the optimisation process, and it was reduced when the population is more inclined towards exploitation, be it local or global.

**Figure 3:**
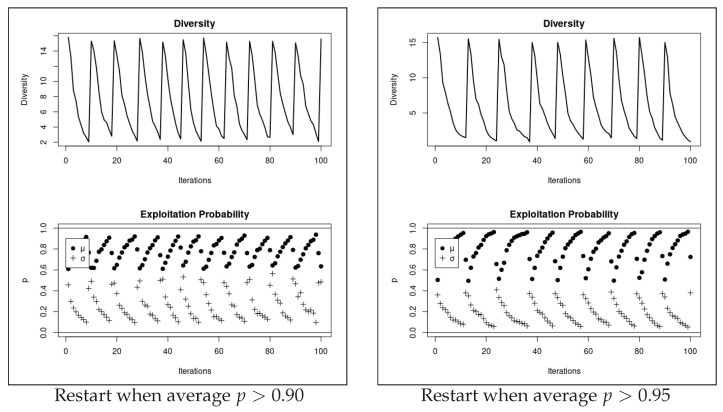
Relation between exploitation probability, *p*, and diversity. This figure illustrates the commencing of the restart mechanism when the dimensional average of p>{0.90,0.95}. In the bottom graphs, μ and σ represent *p*’s average and standard deviation in each iteration. As shown, increased diversity, which is the average distance around the population centre (see top graphs), decreases the *p* values (see bottom graphs), and vice versa.

To demonstrate the evident effect of individual’s restart on *p* over the iterations, a sample run of DFO with Δ=0 is illustrated in Figure 3; here, the behaviour of *p* is visualised during the optimisation process of Rastrigin function where the restart mechanism is triggered when the dimensional average of p>{0.90,0.95}. As shown in the bottom graphs, the black circles, which represent *p*’s average over the dimensions, increase until reaching 0.90 (on the left graph) or 0.95 (on the right graph), when the restart mechanism is triggered. As the graph on the right shows, the average *p* is allowed to increase higher before the restart mechanism is activated. The impact of *p* on diversity can be observed in the top graphs.

#### 3.1.2. Unified DFO with Zone-Relocation (uDFO${}_{z5}$)

**Figure 4:**
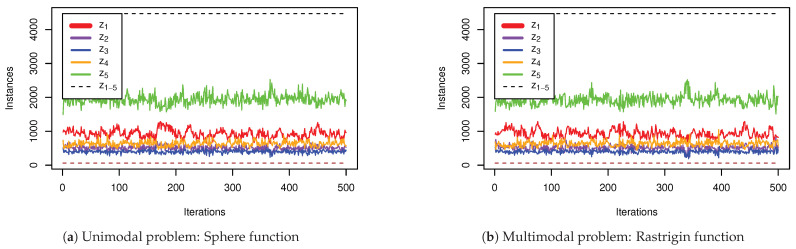
Particle component’s visit-frequency in each zone. These figures illustrate the number of particle components in each zone over the iterations in two representative sample runs, optimising (**a**) Sphere function, which is a unimodal problem, and (**b**) Rastrigin function, as a multimodal problem. This illustrates that, irrespective of the modality and the landscapes of the functions, $z_{5}$ is the most frequently visited zone, and $z_{3}$ is the least visited one.

Figure 1 highlights the exploration and exploitation-related, scaled zone borderlines at x∈{−L,−1,0,0.5,1,R}, and, based on that, the search space is categorised into 5 *zones* ($z_{1-5}$). Using the zones provides a fitting way to investigate the behaviour of the individuals in the context of the unified exploitation probabilities, as well as particle trajectories. In these zones, $z_{2,3}$ are explore-only, $z_{5}$ is exploit-only, and $z_{1,4}$ influence both exploration and exploitation. In other words, zones impacting exploration are $z_{1-4}$, and zones impacting exploitation are $z_{1,4,5}$. Figure 4 illustrates the visit-frequency of particle components in each zone over the iterations, highlighting the most visited zone, $z_{5}$, and the least visited one, $z_{3}$.

**Figure 5:**
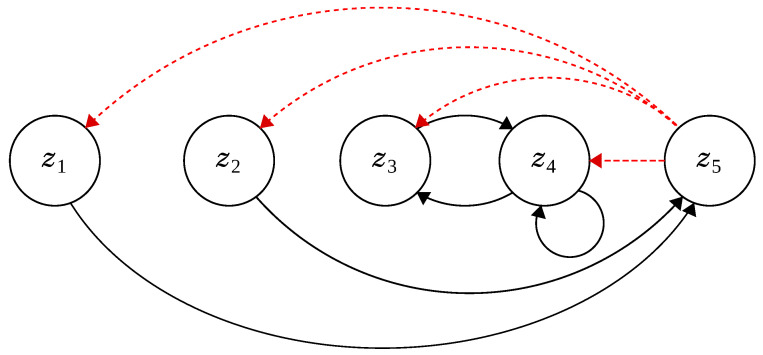
State transition between zones. This figure shows the state transition of components between zones. Transitions from $z_{5}$ are highlighted in red, as dashed lines.

Additionally, having these properties, investigating the state transitions from one zone at time *t* ($x^{t}$) to the next at time t+1 ($x^{t+1}$) provides each particle’s dimensional trajectory, which is illustrated in Figure 5 and summarised below:

- $x^{t}\in z_{1}\to x^{t+1}\in z_{5}$
- $x^{t}\in z_{2}\to x^{t+1}\in \left[1,2\right]\subset z_{5}$,
- $x^{t}\in z_{3}\to x^{t+1}\in z_{4}$,
- $x^{t}\in z_{4}\to x^{t+1}\in \left\{z_{3},z_{4}\right\}$,
- $x^{t}\in z_{5}\to x^{t+1}\in \left\{z_{1},z_{2},z_{3},z_{4}\right\}$.

**Figure 6:**
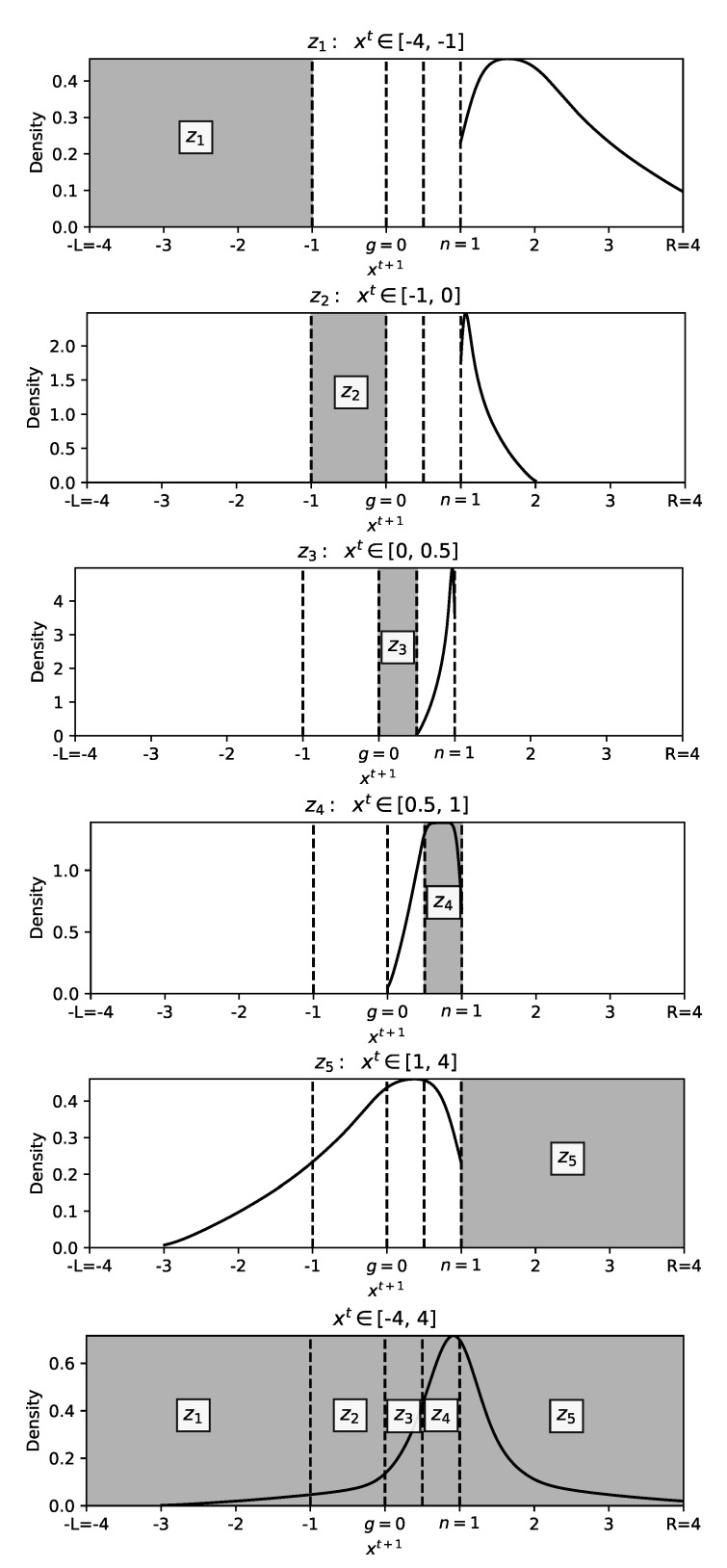
Density plots for transition trajectory of 1 million independent components from each of the zones at time *t* (shaded) to t+1 in one-step updates. The dashed lines represent zones boundaries. The *x*-axes represent the scaled positions in range [−L,R] with L,R=4, g=0, n=1, and the *y*-axes illustrate the trajectory density. For instance, the top graph shows the trajectory density of $x^{t}$ values in $z_{1}$ which are originated from range [−4,−1] at time *t*, and trajected to [1,4] at time t+1. The bottom graph presents the density plot across all zones, highlighting the focus as μ=n. Note that the number of components initialised in each zone is equal.

Following on the state transition, in order to show the trajectory density for each of these transitions, 1,000,000 component updates are initiated from each of the zones with L,R=4. The density plots from different zones are shown in Figure 6; for instance, the top of Figure 6 illustrates the transition of components from $z_{1}$ to $z_{5}$ with higher density of components near *n*. In addition to presenting the density plot for each individual zone, the bottom of Figure 6 shows the trajectory density of the independent updates across *all zones*, illustrating the densest area, which is in line with the search focus being *n*.

State transition analysis allows for devising a strategy to control diversity through particle position’s *zone-relocation*. Observing the density plot for $z_{5}$ in Figure 6 or the state transition from $z_{5}$ in Figure 5, it is evident that particles in $z_{5}$ at time *t* will be relocated to $z_{1-4}$ at time t+1, a unique disseminating possibility only available to components in $z_{5}$.

**Figure 7:**
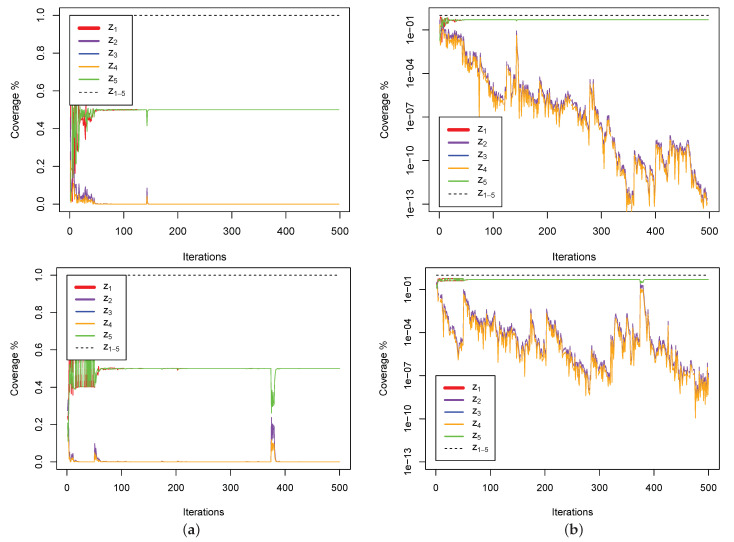
Zones coverage in Sphere (**top**), and Rastrigin (**bottom**) functions. The plots in this figure show the coverage of each zone in each iteration. (**a**) highlights larger updates in the location of *g* or *n* throughout the optimisation process, this is because the coverage ranges of $z_{2,3,4}$ indicate updates in the position of *g* or *n*, while (**b**), which uses the logarithmic scale for the *y*-axis, is used to illustrate the continuous smaller updates in the position of *g* or *n*. The error values at the end of iteration 500 are $2.12\times 10^{-15}$ and $1.73\times 10^{-6}$ respectively.

To better understand each zone’s coverage, the behaviour of the optimiser is investigated in a single dimension of a particle when optimising a unimodal function (Sphere) and a multimodal function (Rastrigin). The plots in Figure 7 illustrate the area covered by each zone. It is shown that $z_{1,5}$ cover the widest range (irrespectively of the problem’s modality), and, as evidenced in Figure 1, $z_{2}$’s coverage area is equal in size to the area covered by $z_{3,4}$. The intuition that the distance between *g* and *n* reduces over time is clearly illustrated in Figure 7b, as manifested by shrinking of areas covered by $z_{2,3,4}$; and, as shown in Figure 7a, the more occasional higher increase of distance between *g* and *n* indicates the identification of a new local optimum (caused by a larger jump which momentarily reduces the coverage of $z_{1,5}$ and increases the coverage of $z_{2,3,4}$).

Given the state transition analysis and the zone coverage for $z_{5}$, another experiment is proposed so that, when the restart mechanism is triggered with $r<\Delta_{\mathrm{dynamic}}$, components are relocated to $z_{5}$. The $\Delta_{\mathrm{dynamic}}$ will be chosen between the better performing algorithm from $\Delta_{\mathrm{dynamic}}=\left\{1/1000p,1/1500p\right\}$. Using this strategy, components are effectively restarted to the exploit-only zone. As a result, while expecting lower diversity, the purpose of zone-relocation experiment is to examine the impact of ‘targeted’ restarts, with potential follow-up exploitation and visits to other zones. The adapted algorithm using the proposed zone-relocation strategy is termed uDFO${}_{z5}$.

## 4. Experiments and Results

This experiments reported in this section examine the results of the exploitation study over a comprehensive benchmark [28], which consists of the functions presented in various sources [29,30,31]. The combined benchmark, CEC05 + CEC13 + ENG13, provides 84 unique problems whose details are presented in Reference [28] and are summarised in Table A1. The benchmark includes functions with the following properties: U: unimodal, M: multimodal, S: separable, NS: non-separable, N: noisy, B: $x^{*}$ on bounds (where $x^{*}$ is the optimum), NV: $x^{*}$ in narrow valley, UB: $x^{*}$ outside initialisation volume, F: neutrality (has flat areas), HC: high conditioning, SD: sensitivity (*f* has one or more sensitive directions), A: asymmetric, D: deceptive ($x^{*}$ is far from next local optimum), and C: composition.

In this section, uDFO with $\Delta_{\mathrm{dynamic}}=\left\{1/1000p,1/1500p\right\}$ and uDFO${}_{z5}$ are compared against the standard DFO (with Δ=0.001) and DFO${}_{\Delta =0}$ (i.e., without the restart mechanism), where the population size is $N_{DFO}=150$. Furthermore, given PSO’s structural similarity to DFO (as outlined in Section 2 and belonging to swarm intelligence family), standard PSO algorithm in two neighbourhood structures, global PSO (GPSO) and local PSO (LPSO), are also used where the population size, $N_{PSO}=30$, ω=0.729844, c=1.49618, and the initial v=0 [31]. Furthermore, DE (DE/best/1) is also used in the experiments, where the population size, $N_{DE}=30$ with F, CR =0.5 [32]. Each algorithm is run 50 times on each test function, and the termination criterion is set to reaching 150,000 function evaluations. The problems’ dimensionality is constant in all trials and is set to D=30.

The metrics used to evaluate the results are *error*: best function value and proximity to known optimal values; and population’s terminal *diversity*: mean distance between individuals and centroid (in PSO, the memory or personal best vectors are used, as opposed to DFOs and DE, where particles positions are used). This measure illustrates the variants’ impact on the population’s diversity to investigate its presence among the population in order to facilitate exploration without hindering the population’s ability to exploit potential optimal solutions. In other words, diversity, alongside the error metric, provides an insight into the inner dynamics of the algorithms.

In total, 33,600 trials (8 algorithms × 84 test functions × 50 runs) are analysed by grouping them in terms of functions and function properties. To analyse the performance of the algorithms over the test functions, Wilcoxon [33] non-parametric tests of significance (p<0.05) is used.

Additionally, the algorithms are applied to tomographic reconstruction, which is an important inverse problem in medical and industrial imaging [34]. One of the purposes of applying the proposed variants to this particular problem is to investigate the performance of the algorithms over problems with increasing dimensionality. The termination criterion is again set to reaching 150,000 function evaluations. In this problem, downsampled standard test images, the Shepp-Logan image phantoms [35], are reconstructed by using two projections. The images have the following dimensions: 25D (5×5), 100D (10×10), 255D (15×15), 400D (20×20), and 625D (25×25).

### Results

Table 1a summarises the performance of the algorithms on 84 test functions, where ‘win’ and ‘loss’ of uDFO${}_{\Delta_{\mathrm{dynamic}}=1/1000p}$ against other algorithms are considered when there is a recorded statistically significant outperformance in terms of the error values. The results demonstrate uDFO${}_{\Delta_{\mathrm{dynamic}}=1/1000p}$’s outperformance in 62%, 75%, 66%, 55%, and 64% of the cases with statistically significant difference, when compared against DFO, DFO${}_{\Delta =0}$ and GPSO, LPSO, and DE, respectively. The details of the algorithms’ performance over each of one of the benchmark are presented in the appendix in Table A2, Table A3, Table A4, Table A5, Table A6, Table A7, Table A8 and Table A9. The tables provide the numerical values for the minimum, maximum, median, mean, and standard deviation associated with each algorithm over each benchmark function. In terms of diversity, and as shown in Table 1b, the statistically significant similarity between the algorithm and standard DFO is evident by observing the number of ties (i.e., 78 out of 84 cases, or 93%). This is expected as per the original intention to take into account the previously reported restart threshold (see Figure 2).

The results of uDFO${}_{\Delta_{\mathrm{dynamic}}=1/1500p}$ against other algorithms are reported in Table 2a. The algorithm’s outperformance in 68%, 78%, 73%, 61%, and 68% of the cases with statistically significant difference are reported. While uDFO${}_{\Delta_{\mathrm{dynamic}}=1/1500p}$ presents higher termination diversity against DFO${}_{\Delta =0}$, GPSO and DE, as shown in Table 2b, the contrary can be observed with DFO and LPSO. The rationale is the consistent value of the restart threshold in standard DFO throughout the optimisation (given Δ=0.001) and the well understood higher diversity of local neighbourhood population in LPSO [36]. In other words, as shown in Figure 2, the reduced rate of the restart mechanism at the tail end of *p* manifests itself in the reduced terminal diversity, as illustrated in the first row of Table 2b.

Table 3 presents the performance comparison of uDFO${}_{z5}$ with other algorithms, including uDFO${}_{\Delta_{\mathrm{dynamic}}=1/1500p}$, which exhibits better performance in terms of error than uDFO${}_{\Delta_{\mathrm{dynamic}}=1/1000p}$. As expected, in terms of error, the winning rates of uDFO${}_{z5}$ and uDFO${}_{\Delta_{\mathrm{dynamic}}=1/1500p}$ are similar when compared against other algorithms, although the latter offers better overall performance. The last rows in Table 3a,b compare uDFO${}_{z5}$ and uDFO${}_{\Delta_{\mathrm{dynamic}}=1/1500p}$, demonstrating the largest number of ties (see the underlined values) as indicators of similarities, which are likely to be influenced by the coverage similarity of holistic and zone-based restarts. However, as expected and explained earlier, uDFO${}_{\Delta_{\mathrm{dynamic}}=1/1500p}$ exhibits higher diversity than uDFO${}_{z5}$.

In order to analyse the error-related strengths and weaknesses of uDFO${}_{\Delta_{\mathrm{dynamic}}=1/1500p}$ and uDFO${}_{z5}$, each of the algorithm pairs are broken down in Table 4 based on fourteen function properties. The total number of function properties (shared by the test functions) is 233. The results demonstrate an overall outperformance of uDFO${}_{\Delta_{\mathrm{dynamic}}=1/1500p}$ and uDFO${}_{z5}$, where the most visible contribution of the unified exploitation approaches can be seen for functions with the following properties {U, S, NS, SD}, while being competitive in {M, NV, A}, and less effective for {N, C}. Among the suitable function properties is non-separable, or NS, where variables interact, making it challenging to decompose the problem into sub-problems; this property is amongst the more demanding in the benchmark and in real-world fitness functions. Further analysis is required to better understand the function properties in the context of the algorithms performance.

Finally, the proposed approaches are trialled on tomographic construction, taking into account problems with larger dimensionality (Table 5 and Table 6). Each algorithm is run 50 times for each problem; therefore, a total of 1500 trials are conducted (6 algorithms × 5 problems × 50 runs). Barring the lowest dimensional problem (25D), the results illustrate the overall competitiveness of uDFO in 94% (15 out of 16), and uDFO${}_{z5}$ in 100% (12 out 12), of the algorithm-problem pairs in high-dimensional problems (see Table 5a,b, respectively).

In summary, while the performance of uDFO${}_{\Delta_{\mathrm{dynamic}}=1/1500p}$ and uDFO${}_{z5}$ are similar on the lower dimensional problem, uDFO${}_{z5}$ demonstrates better performance in all higher-dimensional problems (i.e., 100D, 255D, 400D, 625D), with wider performance gaps as the dimensionality grows (see Table 6). Further experiments are needed to verify the extendibility of performance in other high-dimensional problems.

Among the challenges of the approach is the need for a-priori knowledge of the bounds to feasible solutions. Whilst setting indicative bounds in many real-world problems is practically possible, further investigation is needed in this area. Additionally, although the main computational expense is associated with function evaluation, the impact of calculating exploitation probability, *p*, on the computational cost is a topic for an ongoing research. Furthermore, having tested the approaches on a comprehensive set of test functions, as well as identifying a number of suitable function properties, one of the next steps is applying the methods to other complex real-world problems with known function properties.

## 5. Conclusions

This work presents a framework for analysing the exploitation probabilities in a vector-stripped swarm technique, which is a minimalist numerical optimiser over continuous search spaces. The algorithm’s vector-stripped nature stems from its update equation’s sole reliance on particles’ position vectors, as well as having (other than population size) one tunable parameter, Δ, controlling the component-wise restart of the particles. This work provides an iteration-based zone analysis of particle’s movements in order to establish their exploration and exploitation behaviour. In addition to better understanding the particles’ behaviour, the work focuses on providing a strategy to control the population’s interaction in the search space. This is attempted through a unified exploitation probability, *p*, through (1) uDFO (with $\Delta_{\mathrm{dynamic}}=1/1000p\;\mathrm{and}\;1/1500p$) using a holistic restart, and (2) uDFO${}_{z5}$, which is trialled for the purpose of examining zone-relocation restart mechanism. Both methods allow adaptable dimensional control of the particles.

The proposed approaches are then examined over 84 test functions with a combined 233 function properties, where uDFO${}_{\Delta_{\mathrm{dynamic}}=1/1000p}$ performs better in 62%, 75%, 66%, 55%, and 64% of cases with statistically significant difference when compared against DFO, DFO${}_{\Delta =0}$, GPSO, LPSO, and DE, respectively; and uDFO${}_{\Delta_{\mathrm{dynamic}}=1/1500p}$ in 68%, 78%, 73%, 61%, and 68%; and uDFO${}_{z5}$ in 64%, 78%, 72%, 61%, and 65% of the significant cases.

The performance is then investigated on the high-dimensional tomographic reconstruction problems, where uDFO${}_{\Delta_{\mathrm{dynamic}}=1/1500p}$ and uDFO${}_{z5}$ exhibited better performance in 94% and 100% of the high-dimensional D = {100,255,400,635} algorithm-problem pairs, respectively.

Using minimalist algorithms facilitates analysis in order to better understand the complex underlying behaviour of the particles, such as: particles oscillation around the constantly changing centres, particles’ influence on one another, and understanding the trajectory of particles [11,22,37]. The paper aimed at investigating the exploitation- and exploration-derived zones to inform the behaviour of the population.

Future work includes investigating the exploitation and zone analyses to other swarm optimisers, as well as exploring approaches, to deal with unbounded problems. Furthermore, studying the performance of the presented approaches on dynamically changing environments and studying the combinations of function properties, which benefit from the analysis, are topics for ongoing research.

## Figures and Tables

**Table 1:** Summary of the results for uDFO with $\Delta_{\mathrm{dynamic}}=1/1000p$. The scores indicate uDFO’s wins and losses when compared against other algorithms. uDFO${}_{\Delta_{\mathrm{dynamic}}=1/1000p}$ exhibits outperformance for the error metric in the majority of significant cases (see bold type).

(a) Error					
**Algorithms**	**Win**	**Loss**	**Tie**	**Win Rate**	**Win Rate (Significant Cases)**
uDFO${}_{\Delta_{\mathrm{dynamic}}=1/1000p}$ (vs. DFO)	**8**	5	71	10%	62%
uDFO${}_{\Delta_{\mathrm{dynamic}}=1/1000p}$ (vs. DFO${}_{\Delta =0}$)	**43**	14	27	51%	75%
uDFO${}_{\Delta_{\mathrm{dynamic}}=1/1000p}$ (vs. GPSO)	**43**	22	19	51%	66%
uDFO${}_{\Delta_{\mathrm{dynamic}}=1/1000p}$ (vs. LPSO)	**42**	34	8	50%	55%
uDFO${}_{\Delta_{\mathrm{dynamic}}=1/1000p}$ (vs. DE)	**46**	26	12	55%	64%
**(b) Diversity**					
**Algorithms**	**Win**	**Loss**	**Tie**	**Win Rate**	**Win Rate (Significant Cases)**
uDFO${}_{\Delta_{\mathrm{dynamic}}=1/1000p}$ (vs. DFO)	**4**	2	78	5%	67%
uDFO${}_{\Delta_{\mathrm{dynamic}}=1/1000p}$ (vs. DFO${}_{\Delta =0}$)	**84**	0	0	100%	100%
uDFO${}_{\Delta_{\mathrm{dynamic}}=1/1000p}$ (vs. GPSO)	**62**	19	3	74%	77%
uDFO${}_{\Delta_{\mathrm{dynamic}}=1/1000p}$ (vs. LPSO)	25	**57**	2	30%	30%
uDFO${}_{\Delta_{\mathrm{dynamic}}=1/1000p}$ (vs. DE)	**67**	15	2	80%	82%

**Table 2:** Summary of the results for uDFO with $\Delta_{\mathrm{dynamic}}=1/1500p$. The scores indicate uDFO’s wins and losses when compared against other algorithms. uDFO${}_{\Delta_{\mathrm{dynamic}}=1/1500p}$ exhibits outperformance for the error metric in the majority of significant cases (see bold type).

(a) Error					
**Algorithms**	**Win**	**Loss**	**Tie**	**Win Rate**	**Win Rate (Significant Cases)**
uDFO${}_{\Delta_{\mathrm{dynamic}}=1/1500p}$ (vs. DFO)	**21**	10	53	25%	68%
uDFO${}_{\Delta_{\mathrm{dynamic}}=1/1500p}$ (vs. DFO${}_{\Delta =0}$)	**40**	11	33	48%	78%
uDFO${}_{\Delta_{\mathrm{dynamic}}=1/1500p}$ (vs. GPSO)	**47**	17	20	56%	73%
uDFO${}_{\Delta_{\mathrm{dynamic}}=1/1500p}$ (vs. LPSO)	**45**	29	10	54%	61%
uDFO${}_{\Delta_{\mathrm{dynamic}}=1/1500p}$ (vs. DE)	**47**	22	15	56%	68%
**(b) Diversity**					
**Algorithms**	**Win**	**Loss**	**Tie**	**Win Rate**	**Win Rate (Significant Cases)**
uDFO${}_{\Delta_{\mathrm{dynamic}}=1/1500p}$ (vs. DFO)	0	**84**	0	0%	0%
uDFO${}_{\Delta_{\mathrm{dynamic}}=1/1500p}$ (vs. DFO${}_{\Delta =0}$)	**84**	0	0	100%	100%
uDFO${}_{\Delta_{\mathrm{dynamic}}=1/1500p}$ (vs. GPSO)	**60**	21	3	71%	74%
uDFO${}_{\Delta_{\mathrm{dynamic}}=1/1500p}$ (vs. LPSO)	22	**59**	3	26%	27%
uDFO${}_{\Delta_{\mathrm{dynamic}}=1/1500p}$ (vs. DE)	**67**	16	1	80%	81%

**Table 3:** Summary of the results for uDFO${}_{z5}$. The scores indicate algorithm’s wins and losses when compared against other methods. uDFO${}_{z5}$ exhibits outperformance for the error metric in the majority of significant cases (see bold type), except for uDFO${}_{\Delta_{\mathrm{dynamic}}=1/1500p}$, albeit with the majority of cases in tie states, as underlined.

(a) Error					
**Algorithms**	**Win**	**Loss**	**Tie**	**Win Rate**	**Win Rate (Significant Cases)**
uDFO${}_{z5}$ (vs. DFO)	**25**	14	45	30%	64%
uDFO${}_{z5}$ (vs. DFO${}_{\Delta =0}$)	**39**	11	34	46%	78%
uDFO${}_{z5}$ (vs. GPSO)	**43**	17	24	51%	72%
uDFO${}_{z5}$ (vs. LPSO)	**45**	29	10	54%	61%
uDFO${}_{z5}$ (vs. DE)	**47**	25	12	56%	65%
uDFO${}_{z5}$ (vs. uDFO${}_{\Delta_{\mathrm{dynamic}}=1/1500p}$)	4	**13**	67	5%	24%
**(b) Diversity**					
**Algorithms**	**Win**	**Loss**	**Tie**	**Win Rate**	**Win Rate (Significant Cases)**
uDFO${}_{z5}$ (vs. DFO)	0	**84**	0	0%	0%
uDFO${}_{z5}$ (vs. DFO${}_{\Delta =0}$)	**84**	0	0	100%	100%
uDFO${}_{z5}$ (vs. GPSO)	**57**	21	6	68%	73%
uDFO${}_{z5}$ (vs. LPSO)	22	**60**	2	26%	27%
uDFO${}_{z5}$ (vs. DE)	**67**	16	1	80%	81%
uDFO${}_{z5}$ (vs. uDFO${}_{\Delta_{\mathrm{dynamic}}=1/1500p}$)	0	**45**	39	0%	0%

**Table 4:** Performance comparison by function properties. Bold type indicates significantly lower error by the algorithm for greater number of function instances with a given property.

(a) uDFO${}_{\Delta_{\mathrm{dynamic}}=1/1500p}$											
f **Property**	**Total**	**uDFO**	**DFO**	**uDFO**	**DFO** ${}_{\Delta =0}$	**uDFO**	**GPSO**	**uDFO**	**LPSO**	**uDFO**	**DE**
U: Unimodal	22	**14**	0	8	8	**14**	6	**17**	3	**12**	6
M: Multimodal	62	7	**10**	**32**	3	**33**	11	**28**	26	**35**	16
S: Separable	18	**8**	1	**13**	5	**10**	5	**11**	3	**8**	6
NS: Non-separable	66	**13**	9	**27**	6	**37**	12	**34**	26	**39**	16
N: Noisy	3	0	0	**3**	0	0	**1**	1	**2**	**2**	0
B: $x^{*}$ on bounds	4	**2**	0	1	1	**3**	0	2	2	1	1
NV: $x^{*}$ in narrow val	3	0	**1**	0	0	**2**	0	**2**	1	**2**	1
UB: $x^{*}$ out init vol	2	0	**1**	**2**	0	1	1	1	1	1	1
F: Neutrality	8	0	**2**	**6**	0	**2**	1	1	**7**	1	**4**
HC: High condition	2	0	**1**	0	0	1	1	1	1	1	1
SD: Sensitivity	2	**2**	0	**2**	0	**2**	0	**2**	0	**1**	0
A: Asymmetric	20	**4**	1	**7**	0	**9**	4	7	7	**11**	9
D: Deceptive	2	0	**1**	**1**	0	**1**	0	**1**	0	**2**	0
C: Composition	19	2	**3**	**9**	0	**5**	4	2	**13**	5	**9**
∑	233	**52**	30	**111**	23	**120**	46	**110**	92	**121**	70
%		**63%**	37%	**83%**	17%	**72%**	28%	**54%**	46%	**63**%	37%
**(b) uDFO** ${}_{z5}$											
f **Property**	**Total**	**uDFO** ${}_{z5}$	**DFO**	**uDFO** ${}_{z5}$	**DFO** ${}_{\Delta =0}$	**uDFO** ${}_{z5}$	**GPSO**	**uDFO** ${}_{z5}$	**LPSO**	**uDFO** ${}_{z5}$	**DE**
U: Unimodal	22	**16**	1	7	**8**	**13**	6	**18**	3	**12**	6
M: Multimodal	62	9	**13**	**32**	3	**30**	11	**27**	26	**35**	19
S: Separable	18	**9**	5	**12**	5	**9**	5	**12**	4	**7**	6
NS: Non-separable	66	**16**	9	**27**	6	**34**	12	**33**	25	**40**	19
N: Noisy	3	0	**1**	**2**	0	0	**1**	1	**2**	**1**	0
B: $x^{*}$ on bounds	4	**2**	0	1	**2**	**2**	1	2	2	1	**2**
NV: $x^{*}$ in narrow val	3	**1**	0	**1**	0	**2**	0	**2**	1	**2**	1
UB: $x^{*}$ out init vol	2	1	1	**1**	0	1	1	1	1	1	1
F: Neutrality	8	0	1	**6**	0	1	1	1	**7**	1	**5**
HC: High condition	2	0	0	0	0	1	1	1	1	1	1
SD: Sensitivity	2	**2**	0	**2**	0	**2**	0	**2**	0	**1**	0
A: Asymmetric	20	3	**4**	**7**	0	**7**	3	7	7	**11**	9
D: Deceptive	2	0	**1**	**1**	0	**1**	0	**1**	0	**2**	0
C: Composition	19	2	2	**9**	1	3	**4**	2	**13**	5	**11**
∑	233	**61**	38	**108**	25	**106**	46	**110**	92	**120**	80
%		**62%**	38%	**81%**	19%	**70%**	30%	**54%**	46%	**60%**	40%

**Table 5:** Tomographic Reconstruction: Performance comparison.

(a) uDFO with $\Delta_{\mathrm{dynamic}}=1/1500p$					
**Algorithms**	**D = 25**	**D = 100**	**D = 225**	**D = 400**	**D = 625**
uDFO vs. DFO	--	uDFO	uDFO	DFO	uDFO
uDFO vs. GPSO	--	uDFO	uDFO	uDFO	uDFO
uDFO vs. LPSO	uDFO	uDFO	uDFO *	uDFO *	uDFO *
uDFO vs. DE	uDFO	uDFO	uDFO	uDFO	uDFO
**(b) uDFO** ${}_{z5}$					
**Algorithms**	**D = 25**	**D = 100**	**D = 225**	**D = 400**	**D = 625**
uDFO${}_{z5}$ vs. DFO	--	uDFO${}_{z5}$	uDFO${}_{z5}$	uDFO${}_{z5}$	uDFO${}_{z5}$
uDFO${}_{z5}$ vs. GPSO	--	uDFO${}_{z5}$	uDFO${}_{z5}$	uDFO${}_{z5}$	uDFO${}_{z5}$
uDFO${}_{z5}$ vs. LPSO	uDFO${}_{z5}$	uDFO${}_{z5}$	uDFO${}_{z5}$ *	uDFO${}_{z5}$ *	uDFO${}_{z5}$ *
uDFO${}_{z5}$ vs. DE	uDFO${}_{z5}$	uDFO${}_{z5}$	uDFO${}_{z5}$	uDFO${}_{z5}$	uDFO${}_{z5}$
uDFO${}_{z5}$ vs. uDFO	--	uDFO${}_{z5}$	uDFO${}_{z5}$	uDFO${}_{z5}$	uDFO${}_{z5}$

*: LPSO does not compute solutions for D = {255,400,625}. This is due to a large number of particles components’ off-shooting out of bounds.

**Table 6:** Tomographic Reconstruction: Error values. Bold type indicates outperforming algorithm(s) for each dimension.

	Algorithm	Min	Max	Median	Mean	StdDev
D = 25	**uDFO**	0.00	0.00	0.00	0.00	0.00
	**uDFO${}_{z5}$**	0.00	0.00	0.00	0.00	0.00
	**DFO**	0.00	0.00	0.00	0.00	0.00
	**GPSO**	0.00	0.00	0.00	0.00	0.00
	LPSO	0.00	$5.24\times 10^{-32}$	$3.08\times 10^{-33}$	$7.36\times 10^{-33}$	$1.14\times 10^{-32}$
	DE	$4.24\times 10^{-23}$	$5.94\times 10^{-8}$	$6.08\times 10^{-14}$	$1.42\times 10^{-9}$	$8.40\times 10^{-9}$
D = 100	uDFO	**$1.6806\times 10^{-14}$**	$1.6884\times 10^{-14}$	$1.6810\times 10^{-14}$	$1.6818\times 10^{-14}$	$1.6127\times 10^{-17}$
	**uDFO${}_{z5}$**	**$1.6806\times 10^{-14}$**	**$1.6808\times 10^{-14}$**	**$1.6806\times 10^{-14}$**	**$1.6806\times 10^{-14}$**	**$4.2304\times 10^{-19}$**
	DFO	$1.98\times 10^{-14}$	$1.55\times 10^{-11}$	$5.65\times 10^{-14}$	$4.53\times 10^{-13}$	$2.18\times 10^{-12}$
	GPSO	$9.00\times 10^{1}$	$2.48\times 10^{2}$	$2.05\times 10^{2}$	$1.94\times 10^{2}$	$3.99\times 10^{1}$
	LPSO	$1.17\times 10^{2}$	$2.23\times 10^{2}$	$1.67\times 10^{2}$	$1.67\times 10^{2}$	$2.53\times 10^{1}$
	DE	$1.79\times 10^{-14}$	$2.21\times 10^{-3}$	$9.79\times 10^{-9}$	$7.11\times 10^{-5}$	$3.34\times 10^{-4}$
D = 225	uDFO	$6.02\times 10^{-10}$	$1.98\times 10^{-8}$	$4.49\times 10^{-9}$	$5.77\times 10^{-9}$	$4.34\times 10^{-9}$
	**uDFO${}_{z5}$**	$2.09\times 10^{-11}$	$1.38\times 10^{-9}$	$1.94\times 10^{-10}$	$2.53\times 10^{-10}$	$2.40\times 10^{-10}$
	DFO	$1.45\times 10^{-7}$	$1.49\times 10^{-6}$	$4.15\times 10^{-7}$	$4.75\times 10^{-7}$	$2.48\times 10^{-7}$
	GPSO	$5.54\times 10^{2}$	$7.08\times 10^{2}$	$6.39\times 10^{2}$	$6.42\times 10^{2}$	$3.41\times 10^{1}$
	LPSO	NA	NA	NA	NA	NA
	DE	$8.64\times 10^{-2}$	$6.42\times 10^{}$	$1.74\times 10^{}$	$2.14\times 10^{}$	$1.40\times 10^{}$
D = 400	uDFO	$1.71\times 10^{-5}$	$4.93\times 10^{-5}$	$2.77\times 10^{-5}$	$2.92\times 10^{-5}$	$7.14\times 10^{-6}$
	**uDFO${}_{z5}$**	$1.60\times 10^{-7}$	$2.47\times 10^{-6}$	$6.14\times 10^{-7}$	$7.21\times 10^{-7}$	$4.41\times 10^{-7}$
	DFO	$1.32\times 10^{-5}$	$5.18\times 10^{-5}$	$2.59\times 10^{-5}$	$2.64\times 10^{-5}$	$7.72\times 10^{-6}$
	GPSO	$1.60\times 10^{3}$	$1.86\times 10^{3}$	$1.77\times 10^{3}$	$1.76\times 10^{3}$	$6.46\times 10^{1}$
	LPSO	NA	NA	NA	NA	NA
	DE	$4.24\times 10^{1}$	$1.33\times 10^{2}$	$7.94\times 10^{1}$	$7.91\times 10^{1}$	$1.89\times 10^{1}$
D = 625	uDFO	$1.89\times 10^{-3}$	$4.03\times 10^{-3}$	$2.75\times 10^{-3}$	$2.73\times 10^{-3}$	$4.65\times 10^{-4}$
	**uDFO${}_{z5}$**	$1.33\times 10^{-5}$	$6.11\times 10^{-5}$	$2.97\times 10^{-5}$	$3.13\times 10^{-5}$	$1.03\times 10^{-5}$
	DFO	$1.01\times 10^{-2}$	$2.33\times 10^{-2}$	$1.73\times 10^{-2}$	$1.72\times 10^{-2}$	$2.67\times 10^{-3}$
	GPSO	$3.89\times 10^{3}$	$4.41\times 10^{3}$	$4.15\times 10^{3}$	$4.15\times 10^{3}$	$1.09\times 10^{2}$
	LPSO	NA	NA	NA	NA	NA
	DE	$5.74\times 10^{2}$	$8.02\times 10^{2}$	$6.73\times 10^{2}$	$6.77\times 10^{2}$	$5.17\times 10^{1}$

**Figure A1:**
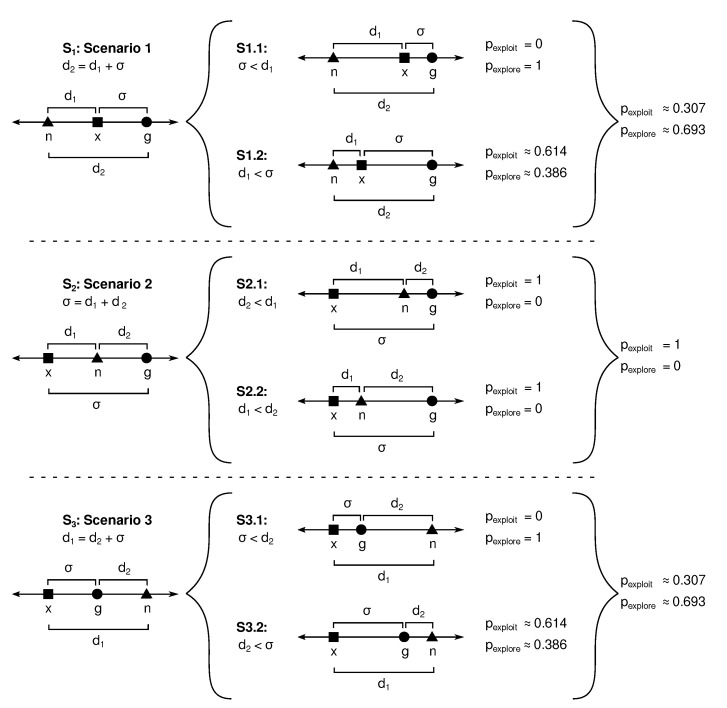
Three scenarios for x≤g, with $d_{1}=\left|n-x\right|$, $d_{2}=\left|g-n\right|$. The analysis also holds for the mirrored scenarios where g≤x.

**Figure A3:**
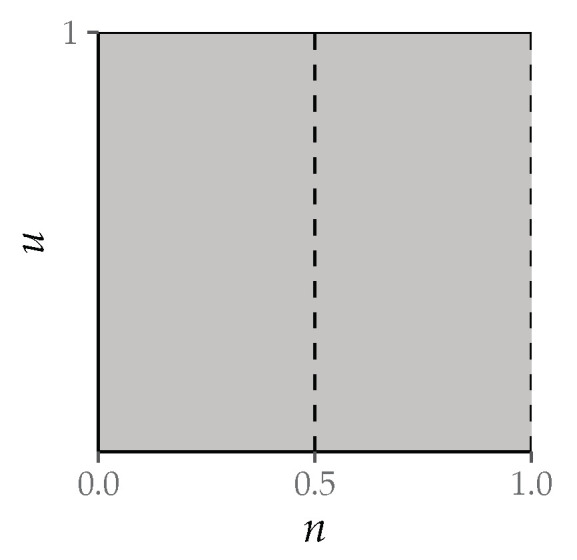
Exploitation probability in scenario 2 (for both x≤n≤g and g≤n≤x).

**Figure A4:**
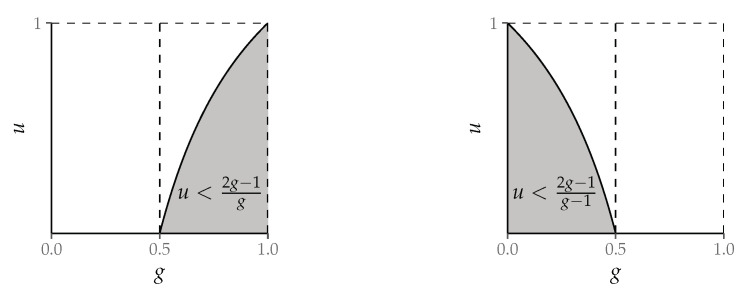
Exploitation probability in scenario 3. (**Left**) x≤g≤n; (**Right**) n≤g≤x.

## Data Availability

Not applicable.

## Appendix A. Iteration-Based Exploration and Exploitation Analysis

For, the iteration-based exploration and exploitation analysis, when x≤g, where *g* is the same for all *x* in the population for each iteration, the following three scenarios can be analysed:

- S${}_{1}$: n≤x≤g S${}_{1.1}$: $\sigma \leq d_{1}$; S${}_{1.2}$: $d_{1}<\sigma$,
- S${}_{2}$: x≤n≤g S${}_{2.1}$: $d_{2}\leq d_{1}$; S${}_{2.2}$: $d_{1}<d_{2}$,
- S${}_{3}$: x≤g≤n S${}_{3.1}$: $\sigma \leq d_{2}$; S${}_{3.2}$: $d_{2}<\sigma$.

The analysis are first presented from an initial state, where the particles are initialised in the search space. Using the small-view exploitation and exploration concepts, the analysis in this section focuses on each scenario (and their corresponding sub-scenarios) and, by extension, the overall impact on each iteration.

### Appendix A.1. Scenario 1: n ≤ x ≤ g

In this scenario, the difference between |n−x| and |g−x|, as well as the value of *u* in the update equation, determine whether $x^{\prime }$ approaches *g*. Given $d_{1}=\left|n-x\right|$ and $d_{2}=\left|g-n\right|$, in the first scenario, $d_{2}=d_{1}+\sigma$ (see Figure A1, top).

Depending on the proximity of *x* to either its best neighbour or the best particle in the swarm, two distinct cases need to be explored. Considering $\sigma \leq d_{1}$ (S1.1 in Figure A1), the exploitation probability, $p_{\mathrm{exploit}}=0$, and the exploration probability is $p_{\mathrm{explore}}=1$, where *x* moves away from *g*. On the other hand, in S1.2, in Figure A1, when $d_{1}<\sigma$, $p_{\mathrm{exploit}}$ and $p_{\mathrm{explore}}$ depend on the proximity of the *x* to *n*, as well as the randomly generated value of *u*.

Based on this and to analyse the probability of exploitation in this scenario, the space is scaled with *n* at the origin and *g* at 1 and *x* is uniformly distributed in [0,1]. Therefore,

$$\begin{array}{cc} n & =0\\ g & =1\\ x^{\prime } & =0+u(1-x)\\ P(\mathrm{exploit}) & =P(|g-x^{\prime }|<|g-x|)\\ \; & =P(1-u(1-x)<1-x)\\ \; & =P\left(u>\frac{x}{1-x}\right)\\ \; & =0.5-\int_{0}^{0.5}\frac{x}{1-x}dx\\ \; & =0.5-({\left[-x-\log\left(1-x\right)\right]}_{0}^{0.5})\\ \; & \approx 0.307. \end{array}$$

This analysis holds for the mirrored case of scenario 1 (i.e., g≤x≤n). The plots in Figure A2 illustrate the exploitation probabilities as shaded areas in S${}_{1}$, and mirrored S${}_{1}$.

### Appendix A.2. Scenario 2: x ≤ n ≤ g

Scaling the space, we have x=0 and g=1. Therefore, there are two possible outcome cases for $x^{\prime }$:

1. $x\leq n\leq g\leq x^{\prime }$,
2. $x\leq n\leq x^{\prime }\leq g$.

Therefore, givenn∈[0,1],u∼U(0,1):

$$\begin{array}{cc} x^{\prime } & =n+u,\\ P(\mathrm{exploit}) & =P(|x^{\prime }-g|<|x-g|)\\ \; & =P(|x^{\prime }-1|<1)\\ \; & =P(|n+u-1|<1)=1\;\mathrm{Always}\;\mathrm{holds},\\ P(\mathrm{exploit}) & =P(n+u-1<1)\;\;\mathrm{for}\;\mathrm{case}\;\left(1\right)\\ \; & =P(n+u<2)=1,\\ P(\mathrm{exploit}) & =P(1-(n+u)<1)\;\;\mathrm{for}\;\mathrm{case}\;\left(2\right)\\ \; & =P(-(n+u)<0)=1. \end{array}$$

The mirrored of this scenario (i.e., g≤n≤x) also holds with exploitation probability equal to 1 (see Figure A3).

### Appendix A.3. Scenario 3: x ≤ g ≤ n

Scaling the space, we have x=0 and n=1. Therefore,

$$\begin{array}{cc} x & =0\\ n & =1\\ P(\mathrm{exploit}) & =P(ug+(1-g)<g)\\ \; & =P(u<\frac{2g-1}{g})\\ \; & =P(u<2-\frac{1}{g})\\ \; & =\int_{0.5}^{1}\left(2-\frac{1}{g}\right)dg\\ \; & {=\left(2g-\log\left(g\right)\right)|}_{0.5}^{1}\\ \; & \approx 0.307. \end{array}$$

The analysis for the mirrored cases in scenario 3 holds (i.e., n≤g≤x). The plots in Figure A4 illustrate the exploitation probabilities in S${}_{3}$, and mirrored S${}_{3}$.

## Appendix B. Benchmark Functions and Algorithms Error Values

This section presents the Benchmark functions in Table A1, which are the combined benchmark of CEC05/13 and ENG13 functions. Based on these functions, the numbering is denoted as $f_{2},f_{4},\ldots$ Function properties, as claimed in the original publications, are: U: unimodal, M: multimodal, S: separable, NS: non-separable, N: noisy, B: $x^{*}$ on bounds, NV: $x^{*}$ in narrow valley, UB: $x^{*}$ outside initialisation volume, F: neutrality (has flat areas), HC: high conditioning, SD: sensitivity (*f* has one or more sensitive directions), A: asymmetric, D: deceptive ($x^{*}$ is far from next local optimum), C: composition.

Furthermore, the error values for algorithms over all the benchmarks are reported in Table A2, Table A3, Table A4, Table A5, Table A6, Table A7, Table A8 and Table A9.

**Table A1:** Benchmark functions.

F No.	Label/Number	Name	Properties	F No.	Label/Number	Name	Properties
**2**	CEC05 F02	Shifted Schwefel 1.2	U, NS	**52**	CEC13 F22	Composition Function 2 (n = 3, Unrotated)	M, S, A, C
**4**	CEC05 F04	Shifted Schwefel 1.2 with noise	U, NS, N	**53**	CEC13 F23	Composition Function 3 (n = 3, Rotated)	M, NS, A, C
**5**	CEC05 F05	Schwefel 2.6, $x^{*}$ on bounds	U, NS, B	**54**	CEC13 F24	Composition Function 4 (n = 3, Rotated)	M, NS, A, C
**6**	CEC05 F06	Shifted Rosenbrock	M, NS, NV	**55**	CEC13 F25	Composition Function 5 (n = 3, Rotated)	M, NS, A, C
**7**	CEC05 F07	Shifted Rotated Griewank without bounds	M, NS, UB	**56**	CEC13 F26	Composition Function 6 (n = 5, Rotated)	M, NS, A, C
**8**	CEC05 F08	Shifted Rotated Ackley, $x^{*}$ on bounds	M, NS, B	**57**	CEC13 F27	Composition Function 7 (n = 5, Rotated)	M, NS, A, C
**11**	CEC05 F11	Shifted Rotated Weierstrass	M, NS	**58**	CEC13 F28	Composition Function 8 (n = 5, Rotated)	M, NS, A, C
**12**	CEC05 F12	Schwefel 2.13	M, NS	**61**	ENG13 F01	Absolute value	U, S
**13**	CEC05 F13	Expanded Extended Griewank + Rosenbrock	M, NS	**62**	ENG13 F02	Ackley	M, NS
**14**	CEC05 F14	Shifted Rotated Expanded Scaffer F6	M, NS	**63**	ENG13 F02 Sh	Shifted Ackley	M, NS
**15**	CEC05 F15	Hybrid Composition	M, NS, F, C	**64**	ENG13 F02 R	Rotated Ackley	M, NS
**16**	CEC05 F16	Rotated Hybrid Composition	M, NS, F, C	**65**	ENG13 F03	Alpine	M, S
**17**	CEC05 F17	Rotated Hybrid Composition with noise	M, NS, N, F, C	**66**	ENG13 F04	Egg holder	M, NS
**18**	CEC05 F18	Rotated Hybrid Composition	M, NS, F, C	**67**	ENG13 F05	Elliptic	U, S
**19**	CEC05 F19	Rotated Hybrid Composition, $x^{*}$ in narrow basin	M, NS, NV, F, C	**68**	ENG13 F05 Sh	Shifted Elliptic	U, S
**20**	CEC05 F20	Rotated Hybrid Composition, $x^{*}$ on bounds	M, NS, B, F, C	**69**	ENG13 F06	Griewank	M, NS
**21**	CEC05 F21	Rotated Hybrid Composition	M, NS, C	**70**	ENG13 F06 Sh	Shifted Griewank	M, NS
**22**	CEC05 F22	Rotated Hybrid Composition, highly conditioned	M, NS, HC, C	**71**	ENG13 F06 R	Rotated Griewank	M, NS
**23**	CEC05 F23	Non-Continuous Rotated Hybrid Composition	M, NS, B, C	**72**	ENG13 F07	Hyperellipsoid	U, S
**24**	CEC05 F24	Rotated Hybrid Composition	M, NS, F, C	**73**	(ENG13 F07 ShR)	Shifted Rotated Hyperellipsoid	U, NS
**25**	CEC05 F25	Rotated Hybrid Composition without Bounds	M, NS, UB, F, C	**74**	ENG13 F08	Michalewicz	M, S
**31**	CEC13 F01	Sphere	U, S	**75**	ENG13 F09	Norwegian	M, NS
**32**	CEC13 F02	Rotated High Conditioned Elliptic	U, NS, HC	**76**	ENG13 F10	Quadric	U, NS
**33**	CEC13 F03	Rotated Bent Cigar	U, NS	**77**	ENG13 F11	Quartic	U, S
**34**	CEC13 F04	Rotated Discus	U, NS, SD, A	**78**	ENG13 F11 N	Quartic/Jong’s F4	U, S, N
**35**	CEC13 F05	Different Powers	U, S, SD	**79**	ENG13 F12	Rastrigin	M, S
**36**	CEC13 F06	Rotated Rosenbrock	M, NS, NV	**80**	ENG13 F12 R	Rotated Rastrigin	M, NS
**37**	CEC13 F07	Rotated Schaffer’s F7	M, NS, A	**81**	ENG13 F13 R	Rosenbrock	M, NS
**38**	CEC13 F08	Rotated Ackley	M, NS, A	**82**	ENG13 F13 R	Rotated Rosenbrock	M, NS
**39**	CEC13 F09	Rotated Weierstrass	M, NS, A	**83**	ENG13 F14	Saloman	M, NS
**40**	CEC13 F10	Rotated Griewank	M, NS	**84**	ENG13 F15	Schaffer 6	M, NS
**41**	CEC13 F11	Rastrigin	M, S, A	**85**	ENG13 F16	Schwefel 1.2	U, NS
**42**	CEC13 F12	Rotated Rastrigin	M, NS, A	**86**	ENG13 F16 R	Rotated Schwefel 1.2	U, NS
**43**	CEC13 F13	Non-Continuous Rotated Rastrigin	M, NS, A	**87**	ENG13 F17	Schwefel 2.6	U, NS
**44**	CEC13 F14	Schwefel	M, NS, A, D	**88**	ENG13 F18	Schwefel 2.13	M, NS
**45**	CEC13 F15	Rotated Schwefel	M, NS, A, D	**89**	ENG13 F19	Schwefel 2.21	U, S
**46**	CEC13 F16	Rotated Katsuura	M, NS, A	**90**	ENG13 F20	Schwefel 2.22	U, S
**47**	CEC13 F17	Lunacek Bi Rastrigin	M, NS	**91**	(ENG13 F20 ShR)	Shifted Rotated Schwefel 2.22	U, NS
**48**	CEC13 F18	Rotated Lunacek Bi Rastrigin	M, NS, A	**92**	ENG13 F21	Shubert	M, NS
**49**	CEC13 F19	Expanded Griewank + Rosenbrock	M, NS	**93**	ENG13 F23	Step	M, S
**50**	CEC13 F20	Expanded Scaffer’s F6	M, NS, A	**94**	ENG13 F24	Vincent	M, S
**51**	CEC13 F21	Composition Function 1 (n = 5, Rotated)	M, NS, A, C	**95**	ENG13 F25	Weierstrass	M, S

**Table A2:** Error values for DFO.

F No.	Min	Max	Median	Mean	StdDev
2	$5.68\times 10^{-14}$	$1.93\times 10^{-12}$	$2.27\times 10^{-13}$	$3.56\times 10^{-13}$	$3.41\times 10^{-13}$
4	$1.31\times 10^{2}$	$8.27\times 10^{3}$	$1.35\times 10^{3}$	$2.24\times 10^{3}$	$2.10\times 10^{3}$
5	$2.61\times 10^{3}$	$6.84\times 10^{3}$	$4.40\times 10^{3}$	$4.55\times 10^{3}$	$1.00\times 10^{3}$
6	$2.35\times 10^{-6}$	7.91	$2.58\times 10^{-3}$	$1.92\times 10^{-1}$	1.12
7	$4.70\times 10^{3}$	$4.70\times 10^{3}$	$4.70\times 10^{3}$	$4.70\times 10^{3}$	$9.09\times 10^{-13}$
8	$2.00\times 10^{1}$	$2.00\times 10^{1}$	$2.00\times 10^{1}$	$2.00\times 10^{1}$	$1.11\times 10^{-2}$
11	$1.56\times 10^{1}$	$3.89\times 10^{1}$	$2.80\times 10^{1}$	$2.87\times 10^{1}$	5.00
12	4.51	$1.85\times 10^{4}$	$1.39\times 10^{3}$	$2.64\times 10^{3}$	$3.55\times 10^{3}$
13	$8.27\times 10^{-1}$	2.32	1.59	1.59	$3.68\times 10^{-1}$
14	$1.24\times 10^{1}$	$1.40\times 10^{1}$	$1.35\times 10^{1}$	$1.35\times 10^{1}$	$3.99\times 10^{-1}$
15	0.00	$5.03\times 10^{2}$	$3.00\times 10^{2}$	$3.02\times 10^{2}$	$1.41\times 10^{2}$
16	$1.57\times 10^{2}$	$5.15\times 10^{2}$	$3.99\times 10^{2}$	$3.54\times 10^{2}$	$9.93\times 10^{1}$
17	$1.71\times 10^{2}$	$9.05\times 10^{2}$	$3.71\times 10^{2}$	$3.75\times 10^{2}$	$1.22\times 10^{2}$
18	$9.24\times 10^{2}$	$1.15\times 10^{3}$	$9.77\times 10^{2}$	$9.93\times 10^{2}$	$5.29\times 10^{1}$
19	$9.28\times 10^{2}$	$1.18\times 10^{3}$	$9.70\times 10^{2}$	$9.87\times 10^{2}$	$5.47\times 10^{1}$
20	$8.00\times 10^{2}$	$1.12\times 10^{3}$	$9.76\times 10^{2}$	$9.88\times 10^{2}$	$5.86\times 10^{1}$
21	$5.00\times 10^{2}$	$1.23\times 10^{3}$	$1.03\times 10^{3}$	$8.71\times 10^{2}$	$3.31\times 10^{2}$
22	$9.65\times 10^{2}$	$1.30\times 10^{3}$	$1.13\times 10^{3}$	$1.13\times 10^{3}$	$8.12\times 10^{1}$
23	$5.00\times 10^{2}$	$1.24\times 10^{3}$	$8.07\times 10^{2}$	$8.36\times 10^{2}$	$3.32\times 10^{2}$
24	$2.00\times 10^{2}$	$1.33\times 10^{3}$	$2.00\times 10^{2}$	$6.24\times 10^{2}$	$5.25\times 10^{2}$
25	$1.67\times 10^{3}$	$1.79\times 10^{3}$	$1.72\times 10^{3}$	$1.72\times 10^{3}$	$2.62\times 10^{1}$
31	0.00	$2.27\times 10^{-13}$	0.00	$2.73\times 10^{-14}$	$7.46\times 10^{-14}$
32	$4.53\times 10^{4}$	$3.04\times 10^{5}$	$1.49\times 10^{5}$	$1.58\times 10^{5}$	$6.54\times 10^{4}$
33	$8.66\times 10^{5}$	$1.52\times 10^{9}$	$6.55\times 10^{7}$	$1.78\times 10^{8}$	$2.96\times 10^{8}$
34	7.51	$1.07\times 10^{3}$	$8.74\times 10^{1}$	$1.52\times 10^{2}$	$1.85\times 10^{2}$
35	0.00	$1.14\times 10^{-13}$	$1.14\times 10^{-13}$	$1.11\times 10^{-13}$	$1.61\times 10^{-14}$
36	$3.75\times 10^{-2}$	$7.11\times 10^{1}$	$1.04\times 10^{1}$	$2.09\times 10^{1}$	$2.30\times 10^{1}$
37	$5.41\times 10^{1}$	$2.16\times 10^{2}$	$1.45\times 10^{2}$	$1.42\times 10^{2}$	$4.17\times 10^{1}$
38	$2.08\times 10^{1}$	$2.10\times 10^{1}$	$2.10\times 10^{1}$	$2.10\times 10^{1}$	$5.72\times 10^{-2}$
39	$2.28\times 10^{1}$	$4.32\times 10^{1}$	$3.05\times 10^{1}$	$3.09\times 10^{1}$	4.06
40	$7.40\times 10^{-3}$	$2.02\times 10^{-1}$	$6.04\times 10^{-2}$	$6.67\times 10^{-2}$	$4.02\times 10^{-2}$
41	0.00	$6.82\times 10^{-13}$	0.00	$3.41\times 10^{-14}$	$9.81\times 10^{-14}$
42	$9.65\times 10^{1}$	$4.15\times 10^{2}$	$2.51\times 10^{2}$	$2.58\times 10^{2}$	$7.41\times 10^{1}$
43	$2.01\times 10^{2}$	$5.86\times 10^{2}$	$3.50\times 10^{2}$	$3.50\times 10^{2}$	$6.53\times 10^{1}$
44	$1.87\times 10^{-1}$	$1.36\times 10^{2}$	9.75	$1.52\times 10^{1}$	$2.50\times 10^{1}$
45	$2.67\times 10^{3}$	$6.05\times 10^{3}$	$4.38\times 10^{3}$	$4.40\times 10^{3}$	$5.78\times 10^{2}$
46	$4.02\times 10^{-1}$	2.75	1.13	1.21	$5.23\times 10^{-1}$
47	$3.04\times 10^{1}$	$3.15\times 10^{1}$	$3.07\times 10^{1}$	$3.07\times 10^{1}$	$2.17\times 10^{-1}$
48	$3.00\times 10^{1}$	$3.00\times 10^{1}$	$3.00\times 10^{1}$	$3.00\times 10^{1}$	$3.94\times 10^{-10}$
49	$9.11\times 10^{-1}$	2.89	1.63	1.67	$4.22\times 10^{-1}$
50	$1.05\times 10^{1}$	$1.34\times 10^{1}$	$1.26\times 10^{1}$	$1.25\times 10^{1}$	$5.72\times 10^{-1}$
51	$1.00\times 10^{2}$	$4.00\times 10^{2}$	$3.00\times 10^{2}$	$3.14\times 10^{2}$	$7.83\times 10^{1}$
52	$1.58\times 10^{1}$	$2.65\times 10^{2}$	$1.27\times 10^{2}$	$1.41\times 10^{2}$	$7.27\times 10^{1}$
53	$4.13\times 10^{3}$	$7.75\times 10^{3}$	$5.30\times 10^{3}$	$5.59\times 10^{3}$	$9.19\times 10^{2}$
54	$2.74\times 10^{2}$	$3.29\times 10^{2}$	$3.01\times 10^{2}$	$3.03\times 10^{2}$	$1.26\times 10^{1}$
55	$2.97\times 10^{2}$	$3.69\times 10^{2}$	$3.26\times 10^{2}$	$3.28\times 10^{2}$	$1.41\times 10^{1}$
56	$2.00\times 10^{2}$	$4.05\times 10^{2}$	$3.76\times 10^{2}$	$3.38\times 10^{2}$	$7.89\times 10^{1}$
57	$9.79\times 10^{2}$	$1.43\times 10^{3}$	$1.22\times 10^{3}$	$1.19\times 10^{3}$	$1.07\times 10^{2}$
58	$1.00\times 10^{2}$	$3.76\times 10^{3}$	$3.00\times 10^{2}$	$8.14\times 10^{2}$	$9.32\times 10^{2}$
61	$3.21\times 10^{-17}$	$8.01\times 10^{-15}$	$4.98\times 10^{-16}$	$1.12\times 10^{-15}$	$1.47\times 10^{-15}$
62	$7.55\times 10^{-15}$	$3.24\times 10^{-14}$	$1.47\times 10^{-14}$	$1.77\times 10^{-14}$	$5.79\times 10^{-15}$
63	$-1.40\times 10^{2}$	$-1.40\;\times \;10^{2}$	$-1.40\;\times \;10^{2}$	$-1.40\;\times \;10^{2}$	$2.15\;\times \;10^{-14}$
64	2.01	7.88	3.49	3.69	1.08
65	$-1.71\times 10^{13}$	$-5.03\;\times \;10^{11}$	$-2.28\;\times \;10^{12}$	$-2.82\;\times \;10^{12}$	$2.78\;\times \;10^{12}$
66	$-2.44\times 10^{4}$	$-1.88\;\times \;10^{4}$	$-2.05\;\times \;10^{4}$	$-2.07\;\times \;10^{4}$	$9.66\;\times \;10^{2}$
67	$1.22\times 10^{-31}$	$2.06\;\times \;10^{-27}$	$1.85\;\times \;10^{-29}$	$1.60\;\times \;10^{-28}$	$4.09\;\times \;10^{-28}$
68	$-4.50\times 10^{2}$	$-4.50\;\times \;10^{2}$	$-4.50\;\times \;10^{2}$	$-4.50\;\times \;10^{2}$	$3.63\;\times \;10^{-14}$
69	0.00	$8.77\;\times \;10^{-2}$	$1.35\;\times \;10^{-2}$	$2.33\;\times \;10^{-2}$	$2.38\;\times \;10^{-2}$
70	$-1.80\times 10^{2}$	$-1.80\;\times \;10^{2}$	$-1.80\;\times \;10^{2}$	$-1.80\;\times \;10^{2}$	$2.09\;\times \;10^{-2}$
71	0.00	$4.65\;\times \;10^{-2}$	$9.86\;\times \;10^{-3}$	$1.12\;\times \;10^{-2}$	$9.71\;\times \;10^{-3}$
72	$4.88\times 10^{-36}$	$1.88\;\times \;10^{-31}$	$5.39\;\times \;10^{-34}$	$6.19\;\times \;10^{-33}$	$2.69\;\times \;10^{-32}$
73	$-4.50\times 10^{2}$	$-4.50\;\times \;10^{2}$	$-4.50\;\times \;10^{2}$	$-4.50\;\times \;10^{2}$	$7.03\;\times \;10^{-14}$
74	$-2.96\times 10^{1}$	$-2.94\;\times \;10^{1}$	$-2.96\;\times \;10^{1}$	$-2.96\;\times \;10^{1}$	$3.85\;\times \;10^{-2}$
75	$-8.39\times 10^{-1}$	$-7.69\;\times \;10^{-1}$	$-8.10\;\times \;10^{-1}$	$-8.07\;\times \;10^{-1}$	$1.61\;\times \;10^{-2}$
76	$9.55\times 10^{-15}$	$3.07\;\times \;10^{-12}$	$8.71\;\times \;10^{-14}$	$2.52\;\times \;10^{-13}$	$5.13\;\times \;10^{-13}$
77	$5.52\times 10^{-69}$	$5.12\;\times \;10^{-63}$	$9.81\;\times \;10^{-66}$	$3.10\;\times \;10^{-64}$	$9.22\;\times \;10^{-64}$
78	−4.06	$-4.69\;\times \;10^{-1}$	−2.33	−2.34	$7.45\;\times \;10^{-1}$
79	0.00	$9.55\;\times \;10^{-10}$	0.00	$2.09\;\times \;10^{-11}$	$1.35\;\times \;10^{-10}$
80	$6.17\times 10^{1}$	$2.22\;\times \;10^{2}$	$1.15\;\times \;10^{2}$	$1.19\;\times \;10^{2}$	$3.15\;\times \;10^{1}$
81	$2.83\times 10^{-8}$	1.40	$2.15\;\times \;10^{-3}$	$1.09\;\times \;10^{-1}$	$3.54\;\times \;10^{-1}$
82	6.14	$1.29\;\times \;10^{4}$	$2.00\;\times \;10^{1}$	$6.24\;\times \;10^{2}$	$2.16\;\times \;10^{3}$
83	$1.73\times 10^{-1}$	$2.45\;\times \;10^{-1}$	$2.00\;\times \;10^{-1}$	$1.99\;\times \;10^{-1}$	$1.85\;\times \;10^{-2}$
84	$5.83\times 10^{-2}$	1.57	$4.89\;\times \;10^{-1}$	$5.81\;\times \;10^{-1}$	$3.36\;\times \;10^{-1}$
85	$4.76\times 10^{-15}$	$1.95\;\times \;10^{-12}$	$7.92\;\times \;10^{-14}$	$1.88\;\times \;10^{-13}$	$3.33\;\times \;10^{-13}$
86	$9.73\times 10^{-15}$	$1.78\;\times \;10^{-10}$	$7.35\;\times \;10^{-13}$	$8.45\;\times \;10^{-12}$	$2.78\;\times \;10^{-11}$
87	$2.27\times 10^{3}$	$7.29\;\times \;10^{3}$	$4.66\;\times \;10^{3}$	$4.53\;\times \;10^{3}$	$1.18\;\times \;10^{3}$
88	6.20	$2.46\;\times \;10^{4}$	$2.53\;\times \;10^{3}$	$3.74\;\times \;10^{3}$	$3.99\;\times \;10^{3}$
89	$1.50\times 10^{-5}$	$1.13\;\times \;10^{-3}$	$1.25\;\times \;10^{-4}$	$2.28\;\times \;10^{-4}$	$2.48\;\times \;10^{-4}$
90	$1.14\times 10^{-13}$	$1.71\;\times \;10^{-13}$	$1.14\;\times \;10^{-13}$	$1.26\;\times \;10^{-13}$	$2.38\;\times \;10^{-14}$
91	$-4.50\times 10^{2}$	$-4.33\;\times \;10^{2}$	$-4.48\;\times \;10^{2}$	$-4.45\;\times \;10^{2}$	4.47
92	$-3.87\times 10^{34}$	$-9.21\;\times \;10^{33}$	$-1.89\;\times \;10^{34}$	$-2.10\;\times \;10^{34}$	$6.63\;\times \;10^{33}$
93	0.00	0.00	0.00	0.00	0.00
94	0.00	0.00	0.00	0.00	0.00
95	0.00	0.00	0.00	0.00	0.00

**Table A3:** Error values for DFO${}_{\Delta =0}$.

F No.	Min	Max	Median	Mean	StdDev
2	$5.68\times 10^{-14}$	$1.88\;\times \;10^{-12}$	$5.68\;\times \;10^{-14}$	$1.51\;\times \;10^{-13}$	$3.03\;\times \;10^{-13}$
4	$4.84\times 10^{3}$	$7.30\;\times \;10^{4}$	$2.93\;\times \;10^{4}$	$3.14\;\times \;10^{4}$	$1.40\;\times \;10^{4}$
5	$2.36\times 10^{3}$	$7.22\;\times \;10^{3}$	$3.88\;\times \;10^{3}$	$4.08\;\times \;10^{3}$	$1.05\;\times \;10^{3}$
6	$3.54\times 10^{-8}$	4.01	$4.06\;\times \;10^{-4}$	1.36	1.91
7	$4.70\times 10^{3}$	$4.70\;\times \;10^{3}$	$4.70\;\times \;10^{3}$	$4.70\;\times \;10^{3}$	$8.69\;\times \;10^{-1}$
8	$2.00\times 10^{1}$	$2.00\;\times \;10^{1}$	$2.00\;\times \;10^{1}$	$2.00\;\times \;10^{1}$	$5.17\;\times \;10^{-3}$
11	$1.94\times 10^{1}$	$3.89\;\times \;10^{1}$	$2.99\;\times \;10^{1}$	$2.95\;\times \;10^{1}$	4.70
12	$1.51\times 10^{2}$	$8.36\;\times \;10^{4}$	$1.09\;\times \;10^{4}$	$1.97\;\times \;10^{4}$	$2.24\;\times \;10^{4}$
13	4.35	$2.33\;\times \;10^{1}$	9.50	$1.01\;\times \;10^{1}$	4.44
14	$1.24\times 10^{1}$	$1.41\;\times \;10^{1}$	$1.36\;\times \;10^{1}$	$1.36\;\times \;10^{1}$	$3.87\;\times \;10^{-1}$
15	$4.00\times 10^{2}$	$9.00\;\times \;10^{2}$	$5.55\;\times \;10^{2}$	$5.64\;\times \;10^{2}$	$1.24\;\times \;10^{2}$
16	$1.97\times 10^{2}$	$5.05\;\times \;10^{2}$	$3.44\;\times \;10^{2}$	$3.51\;\times \;10^{2}$	$9.54\;\times \;10^{1}$
17	$2.52\times 10^{2}$	$7.62\;\times \;10^{2}$	$4.85\;\times \;10^{2}$	$4.90\;\times \;10^{2}$	$1.30\;\times \;10^{2}$
18	$9.28\times 10^{2}$	$1.24\;\times \;10^{3}$	$1.02\;\times \;10^{3}$	$1.03\;\times \;10^{3}$	$6.71\;\times \;10^{1}$
19	$9.41\times 10^{2}$	$1.15\;\times \;10^{3}$	$1.01\;\times \;10^{3}$	$1.01\;\times \;10^{3}$	$4.66\;\times \;10^{1}$
20	$9.29\times 10^{2}$	$1.19\;\times \;10^{3}$	$1.01\;\times \;10^{3}$	$1.02\;\times \;10^{3}$	$5.83\;\times \;10^{1}$
21	$5.00\times 10^{2}$	$1.23\;\times \;10^{3}$	$1.16\;\times \;10^{3}$	$8.99\;\times \;10^{2}$	$3.38\;\times \;10^{2}$
22	$9.78\times 10^{2}$	$1.34\;\times \;10^{3}$	$1.14\;\times \;10^{3}$	$1.16\;\times \;10^{3}$	$8.49\;\times \;10^{1}$
23	$5.00\times 10^{2}$	$1.22\;\times \;10^{3}$	$5.00\;\times \;10^{2}$	$7.82\;\times \;10^{2}$	$3.32\;\times \;10^{2}$
24	$2.00\times 10^{2}$	$1.36\;\times \;10^{3}$	$1.24\;\times \;10^{3}$	$1.05\;\times \;10^{3}$	$4.12\;\times \;10^{2}$
25	$1.69\times 10^{3}$	$1.82\;\times \;10^{3}$	$1.77\;\times \;10^{3}$	$1.77\;\times \;10^{3}$	$2.87\;\times \;10^{1}$
31	0.00	$4.55\;\times \;10^{-13}$	$2.27\;\times \;10^{-13}$	$2.50\;\times \;10^{-13}$	$8.28\;\times \;10^{-14}$
32	$4.23\times 10^{4}$	$3.72\;\times \;10^{5}$	$1.43\;\times \;10^{5}$	$1.57\;\times \;10^{5}$	$7.59\;\times \;10^{4}$
33	$2.87\times 10^{6}$	$1.16\;\times \;10^{9}$	$6.59\;\times \;10^{7}$	$1.69\;\times \;10^{8}$	$2.65\;\times \;10^{8}$
34	$3.25\times 10^{1}$	$1.14\;\times \;10^{4}$	$4.72\;\times \;10^{2}$	$1.42\;\times \;10^{3}$	$2.44\;\times \;10^{3}$
35	$1.14\times 10^{-13}$	$4.55\;\times \;10^{-13}$	$2.27\;\times \;10^{-13}$	$2.21\;\times \;10^{-13}$	$8.09\;\times \;10^{-14}$
36	$1.05\times 10^{-2}$	$6.81\;\times \;10^{1}$	9.72	$1.44\;\times \;10^{1}$	$1.56\;\times \;10^{1}$
37	$5.57\times 10^{1}$	$5.15\;\times \;10^{2}$	$1.41\;\times \;10^{2}$	$1.45\;\times \;10^{2}$	$6.89\;\times \;10^{1}$
38	$2.07\times 10^{1}$	$2.11\;\times \;10^{1}$	$2.10\;\times \;10^{1}$	$2.10\;\times \;10^{1}$	$9.73\;\times \;10^{-2}$
39	$2.09\times 10^{1}$	$3.88\;\times \;10^{1}$	$3.18\;\times \;10^{1}$	$3.14\;\times \;10^{1}$	4.01
40	$9.86\times 10^{-3}$	$3.81\;\times \;10^{-1}$	$7.61\;\times \;10^{-2}$	$8.00\;\times \;10^{-2}$	$5.74\;\times \;10^{-2}$
41	$1.14\times 10^{2}$	$3.53\;\times \;10^{2}$	$2.29\;\times \;10^{2}$	$2.27\;\times \;10^{2}$	$5.80\;\times \;10^{1}$
42	$1.45\times 10^{2}$	$4.25\;\times \;10^{2}$	$2.59\;\times \;10^{2}$	$2.58\;\times \;10^{2}$	$6.50\;\times \;10^{1}$
43	$2.00\times 10^{2}$	$6.14\;\times \;10^{2}$	$3.55\;\times \;10^{2}$	$3.68\;\times \;10^{2}$	$9.33\;\times \;10^{1}$
44	$2.42\times 10^{3}$	$5.32\;\times \;10^{3}$	$3.64\;\times \;10^{3}$	$3.67\;\times \;10^{3}$	$5.68\;\times \;10^{2}$
45	$3.00\times 10^{3}$	$6.35\;\times \;10^{3}$	$4.63\;\times \;10^{3}$	$4.61\;\times \;10^{3}$	$6.80\;\times \;10^{2}$
46	$1.57\times 10^{-1}$	2.30	$9.64\;\times \;10^{-1}$	1.06	$4.67\;\times \;10^{-1}$
47	$1.38\times 10^{2}$	$4.18\;\times \;10^{2}$	$2.61\;\times \;10^{2}$	$2.65\;\times \;10^{2}$	$6.88\;\times \;10^{1}$
48	$1.57\times 10^{2}$	$5.38\;\times \;10^{2}$	$2.69\;\times \;10^{2}$	$2.86\;\times \;10^{2}$	$8.49\;\times \;10^{1}$
49	7.93	$3.93\;\times \;10^{1}$	$1.62\;\times \;10^{1}$	$1.89\;\times \;10^{1}$	8.37
50	$1.01\times 10^{1}$	$1.37\;\times \;10^{1}$	$1.27\;\times \;10^{1}$	$1.26\;\times \;10^{1}$	$7.54\;\times \;10^{-1}$
51	$1.00\times 10^{2}$	$4.00\;\times \;10^{2}$	$3.00\;\times \;10^{2}$	$3.06\;\times \;10^{2}$	$7.40\;\times \;10^{1}$
52	$2.65\times 10^{3}$	$7.24\;\times \;10^{3}$	$5.01\;\times \;10^{3}$	$4.85\;\times \;10^{3}$	$1.10\;\times \;10^{3}$
53	$3.61\times 10^{3}$	$7.66\;\times \;10^{3}$	$5.64\;\times \;10^{3}$	$5.66\;\times \;10^{3}$	$9.98\;\times \;10^{2}$
54	$2.72\times 10^{2}$	$3.43\;\times \;10^{2}$	$3.01\;\times \;10^{2}$	$3.02\;\times \;10^{2}$	$1.60\;\times \;10^{1}$
55	$3.08\times 10^{2}$	$3.69\;\times \;10^{2}$	$3.36\;\times \;10^{2}$	$3.36\;\times \;10^{2}$	$1.46\;\times \;10^{1}$
56	$2.00\times 10^{2}$	$4.01\;\times \;10^{2}$	$3.77\;\times \;10^{2}$	$3.47\;\times \;10^{2}$	$7.04\;\times \;10^{1}$
57	$8.86\times 10^{2}$	$1.49\;\times \;10^{3}$	$1.18\;\times \;10^{3}$	$1.19\;\times \;10^{3}$	$1.12\;\times \;10^{2}$
58	$1.00\times 10^{2}$	$3.18\;\times \;10^{3}$	$3.00\;\times \;10^{2}$	$9.83\;\times \;10^{2}$	$9.83\;\times \;10^{2}$
61	$5.05\times 10^{-29}$	$2.78\;\times \;10^{-13}$	$2.47\;\times \;10^{-22}$	$5.66\;\times \;10^{-15}$	$3.93\;\times \;10^{-14}$
62	1.34	$1.35\;\times \;10^{1}$	3.49	4.04	2.21
63	$-1.38\times 10^{2}$	$-1.21\;\times \;10^{2}$	$-1.36\;\times \;10^{2}$	$-1.34\;\times \;10^{2}$	4.52
64	1.90	$1.31\;\times \;10^{1}$	3.93	4.70	2.50
65	$-3.01\times 10^{11}$	$-4.11\;\times \;10^{8}$	$-1.41\;\times \;10^{10}$	$-4.14\;\times \;10^{10}$	$6.88\;\times \;10^{10}$
66	$-1.56\times 10^{4}$	$-9.98\;\times \;10^{3}$	$-1.32\;\times \;10^{4}$	$-1.29\;\times \;10^{4}$	$1.38\;\times \;10^{3}$
67	$1.04\times 10^{-77}$	$5.12\;\times \;10^{-68}$	$6.54\;\times \;10^{-73}$	$1.52\;\times \;10^{-69}$	$7.52\;\times \;10^{-69}$
68	$-4.50\times 10^{2}$	$-4.50\;\times \;10^{2}$	$-4.50\;\times \;10^{2}$	$-4.50\;\times \;10^{2}$	$1.71\;\times \;10^{-13}$
69	0.00	$3.02\;\times \;10^{-1}$	$1.85\;\times \;10^{-2}$	$3.15\;\times \;10^{-2}$	$4.85\;\times \;10^{-2}$
70	$-1.80\times 10^{2}$	$-1.80\;\times \;10^{2}$	$-1.80\;\times \;10^{2}$	$-1.80\;\times \;10^{2}$	$4.05\;\times \;10^{-2}$
71	0.00	$6.87\;\times \;10^{-2}$	$1.23\;\times \;10^{-2}$	$1.45\;\times \;10^{-2}$	$1.27\;\times \;10^{-2}$
72	$2.40\times 10^{-81}$	$1.66\;\times \;10^{-74}$	$3.48\;\times \;10^{-78}$	$1.11\;\times \;10^{-75}$	$3.23\;\times \;10^{-75}$
73	$-4.50\times 10^{2}$	$-4.50\;\times \;10^{2}$	$-4.50\;\times \;10^{2}$	$-4.50\;\times \;10^{2}$	$1.07\;\times \;10^{-13}$
74	$-2.59\times 10^{1}$	$-2.04\;\times \;10^{1}$	$-2.35\;\times \;10^{1}$	$-2.34\;\times \;10^{1}$	1.33
75	$-8.09\times 10^{-1}$	$-7.31\;\times \;10^{-1}$	$-7.69\;\times \;10^{-1}$	$-7.71\;\times \;10^{-1}$	$1.78\;\times \;10^{-2}$
76	$5.91\times 10^{-20}$	$3.26\;\times \;10^{-11}$	$1.61\;\times \;10^{-16}$	$7.19\;\times \;10^{-13}$	$4.62\;\times \;10^{-12}$
77	$9.78\times 10^{-150}$	$3.63\;\times \;10^{-139}$	$4.36\;\times \;10^{-143}$	$1.54\;\times \;10^{-140}$	$6.41\;\times \;10^{-140}$
78	−3.69	2.57	−1.88	−1.56	1.41
79	$5.07\times 10^{1}$	$2.33\;\times \;10^{2}$	$1.12\;\times \;10^{2}$	$1.13\;\times \;10^{2}$	$3.29\;\times \;10^{1}$
80	$5.57\times 10^{1}$	$2.26\;\times \;10^{2}$	$1.13\;\times \;10^{2}$	$1.17\;\times \;10^{2}$	$3.30\;\times \;10^{1}$
81	$4.56\times 10^{-9}$	$1.18\;\times \;10^{1}$	$2.11\;\times \;10^{-4}$	1.43	2.37
82	4.85	$7.52\;\times \;10^{3}$	$1.65\;\times \;10^{1}$	$2.18\;\times \;10^{2}$	$1.06\;\times \;10^{3}$
83	$3.16\times 10^{-1}$	4.21	$5.52\;\times \;10^{-1}$	$7.45\;\times \;10^{-1}$	$5.94\;\times \;10^{-1}$
84	$1.00\times 10^{1}$	$1.35\;\times \;10^{1}$	$1.22\;\times \;10^{1}$	$1.20\;\times \;10^{1}$	$9.44\;\times \;10^{-1}$
85	$3.97\times 10^{-20}$	$1.23\;\times \;10^{-11}$	$1.13\;\times \;10^{-16}$	$3.61\;\times \;10^{-13}$	$1.86\;\times \;10^{-12}$
86	$2.34\times 10^{-18}$	$9.19\;\times \;10^{-10}$	$2.19\;\times \;10^{-14}$	$4.99\;\times \;10^{-11}$	$1.60\;\times \;10^{-10}$
87	$1.93\times 10^{3}$	$6.19\;\times \;10^{3}$	$3.85\;\times \;10^{3}$	$3.93\;\times \;10^{3}$	$1.07\;\times \;10^{3}$
88	$2.58\times 10^{1}$	$1.62\;\times \;10^{5}$	$1.65\;\times \;10^{4}$	$2.74\;\times \;10^{4}$	$3.52\;\times \;10^{4}$
89	$1.09\times 10^{-7}$	$3.63\;\times \;10^{-5}$	$3.13\;\times \;10^{-6}$	$5.27\;\times \;10^{-6}$	$6.40\;\times \;10^{-6}$
90	$5.68\times 10^{-14}$	$1.14\;\times \;10^{-8}$	$1.71\;\times \;10^{-13}$	$3.28\;\times \;10^{-10}$	$1.73\;\times \;10^{-9}$
91	$-4.50\times 10^{2}$	$-3.25\;\times \;10^{2}$	$-4.48\;\times \;10^{2}$	$-4.38\;\times \;10^{2}$	$3.20\;\times \;10^{1}$
92	$-9.89\times 10^{28}$	$-5.06\;\times \;10^{23}$	$-1.11\;\times \;10^{26}$	$-4.52\;\times \;10^{27}$	$1.51\;\times \;10^{28}$
93	0.00	$3.08\;\times \;10^{2}$	9.00	$3.00\;\times \;10^{1}$	$6.45\;\times \;10^{1}$
94	0.00	$2.84\;\times \;10^{-14}$	0.00	$2.13\;\times \;10^{-15}$	$5.42\;\times \;10^{-15}$
95	8.96	$2.58\;\times \;10^{1}$	$1.61\;\times \;10^{1}$	$1.58\;\times \;10^{1}$	3.23

**Table A4:** Error values for uDFO${}_{\Delta_{\mathrm{dynmaic}}=1/1000p}$.

F No.	Min	Max	Median	Mean	StdDev
2	$5.68\times 10^{-14}$	$1.02\;\times \;10^{-12}$	$2.27\;\times \;10^{-13}$	$3.09\;\times \;10^{-13}$	$1.82\;\times \;10^{-13}$
4	$2.13\times 10^{2}$	$8.68\;\times \;10^{3}$	$2.07\;\times \;10^{3}$	$2.55\;\times \;10^{3}$	$2.11\;\times \;10^{3}$
5	$2.77\times 10^{3}$	$7.28\;\times \;10^{3}$	$4.54\;\times \;10^{3}$	$4.72\;\times \;10^{3}$	$1.10\;\times \;10^{3}$
6	$2.91\times 10^{-6}$	$8.78\;\times \;10^{1}$	$8.17\;\times \;10^{-3}$	5.06	$1.72\;\times \;10^{1}$
7	$4.70\times 10^{3}$	$4.70\;\times \;10^{3}$	$4.70\;\times \;10^{3}$	$4.70\;\times \;10^{3}$	$1.20\;\times \;10^{-12}$
8	$2.00\times 10^{1}$	$2.00\;\times \;10^{1}$	$2.00\;\times \;10^{1}$	$2.00\;\times \;10^{1}$	$9.12\;\times \;10^{-3}$
11	$2.17\times 10^{1}$	$4.12\;\times \;10^{1}$	$2.84\;\times \;10^{1}$	$2.90\;\times \;10^{1}$	4.67
12	$3.31\times 10^{1}$	$2.48\;\times \;10^{4}$	$2.70\;\times \;10^{3}$	$4.25\;\times \;10^{3}$	$4.80\;\times \;10^{3}$
13	$7.97\times 10^{-1}$	2.50	1.59	1.62	$3.50\;\times \;10^{-1}$
14	$1.14\times 10^{1}$	$1.40\;\times \;10^{1}$	$1.35\;\times \;10^{1}$	$1.35\;\times \;10^{1}$	$4.80\;\times \;10^{-1}$
15	0.00	$5.11\;\times \;10^{2}$	$3.00\;\times \;10^{2}$	$2.87\;\times \;10^{2}$	$1.46\;\times \;10^{2}$
16	$9.94\times 10^{1}$	$5.52\;\times \;10^{2}$	$3.22\;\times \;10^{2}$	$3.29\;\times \;10^{2}$	$1.17\;\times \;10^{2}$
17	$1.62\times 10^{2}$	$8.03\;\times \;10^{2}$	$4.22\;\times \;10^{2}$	$3.97\;\times \;10^{2}$	$1.23\;\times \;10^{2}$
18	$9.19\times 10^{2}$	$1.15\;\times \;10^{3}$	$9.62\;\times \;10^{2}$	$9.81\;\times \;10^{2}$	$5.42\;\times \;10^{1}$
19	$9.23\times 10^{2}$	$1.16\;\times \;10^{3}$	$9.69\;\times \;10^{2}$	$9.86\;\times \;10^{2}$	$5.48\;\times \;10^{1}$
20	$9.25\times 10^{2}$	$1.14\;\times \;10^{3}$	$9.76\;\times \;10^{2}$	$9.91\;\times \;10^{2}$	$5.54\;\times \;10^{1}$
21	$5.00\times 10^{2}$	$1.25\;\times \;10^{3}$	$1.18\;\times \;10^{3}$	$9.38\;\times \;10^{2}$	$3.14\;\times \;10^{2}$
22	$9.83\times 10^{2}$	$1.33\;\times \;10^{3}$	$1.13\;\times \;10^{3}$	$1.14\;\times \;10^{3}$	$7.75\;\times \;10^{1}$
23	$5.00\times 10^{2}$	$1.24\;\times \;10^{3}$	$8.00\;\times \;10^{2}$	$8.07\;\times \;10^{2}$	$3.20\;\times \;10^{2}$
24	$2.00\times 10^{2}$	$1.36\;\times \;10^{3}$	$6.29\;\times \;10^{2}$	$7.31\;\times \;10^{2}$	$5.39\;\times \;10^{2}$
25	$1.68\times 10^{3}$	$1.79\;\times \;10^{3}$	$1.73\;\times \;10^{3}$	$1.73\;\times \;10^{3}$	$2.52\;\times \;10^{1}$
31	0.00	$2.27\;\times \;10^{-13}$	0.00	$2.27\;\times \;10^{-14}$	$6.89\;\times \;10^{-14}$
32	$5.27\times 10^{4}$	$3.33\;\times \;10^{5}$	$1.65\;\times \;10^{5}$	$1.76\;\times \;10^{5}$	$6.65\;\times \;10^{4}$
33	$2.25\times 10^{6}$	$2.13\;\times \;10^{9}$	$7.73\;\times \;10^{7}$	$1.72\;\times \;10^{8}$	$3.21\;\times \;10^{8}$
34	$1.79\times 10^{1}$	$5.06\;\times \;10^{2}$	$9.30\;\times \;10^{1}$	$1.28\;\times \;10^{2}$	$1.18\;\times \;10^{2}$
35	0.00	$1.14\;\times \;10^{-13}$	$1.14\;\times \;10^{-13}$	$1.11\;\times \;10^{-13}$	$1.61\;\times \;10^{-14}$
36	$5.23\times 10^{-3}$	$7.47\;\times \;10^{1}$	$1.02\;\times \;10^{1}$	$1.75\;\times \;10^{1}$	$1.94\;\times \;10^{1}$
37	$6.51\times 10^{1}$	$2.57\;\times \;10^{2}$	$1.31\;\times \;10^{2}$	$1.34\;\times \;10^{2}$	$3.49\;\times \;10^{1}$
38	$2.08\times 10^{1}$	$2.10\;\times \;10^{1}$	$2.09\;\times \;10^{1}$	$2.09\;\times \;10^{1}$	$5.85\;\times \;10^{-2}$
39	$2.22\times 10^{1}$	$3.95\;\times \;10^{1}$	$3.19\;\times \;10^{1}$	$3.12\;\times \;10^{1}$	4.23
40	$2.22\times 10^{-2}$	$3.10\;\times \;10^{-1}$	$5.79\;\times \;10^{-2}$	$7.75\;\times \;10^{-2}$	$5.63\;\times \;10^{-2}$
41	0.00	$1.14\;\times \;10^{-13}$	0.00	$1.93\;\times \;10^{-14}$	$2.95\;\times \;10^{-14}$
42	$1.15\times 10^{2}$	$3.98\;\times \;10^{2}$	$2.21\;\times \;10^{2}$	$2.31\;\times \;10^{2}$	$6.69\;\times \;10^{1}$
43	$2.11\times 10^{2}$	$5.82\;\times \;10^{2}$	$3.45\;\times \;10^{2}$	$3.51\;\times \;10^{2}$	$7.90\;\times \;10^{1}$
44	1.24	$3.52\;\times \;10^{1}$	6.35	7.93	5.58
45	$2.91\times 10^{3}$	$5.60\;\times \;10^{3}$	$4.17\;\times \;10^{3}$	$4.14\;\times \;10^{3}$	$5.57\;\times \;10^{2}$
46	$3.31\times 10^{-1}$	3.03	1.31	1.29	$5.53\;\times \;10^{-1}$
47	$3.05\times 10^{1}$	$3.11\;\times \;10^{1}$	$3.06\;\times \;10^{1}$	$3.07\;\times \;10^{1}$	$1.56\;\times \;10^{-1}$
48	$3.00\times 10^{1}$	$3.09\;\times \;10^{1}$	$3.00\;\times \;10^{1}$	$3.00\;\times \;10^{1}$	$1.20\;\times \;10^{-1}$
49	$8.11\times 10^{-1}$	3.46	1.66	1.70	$5.37\;\times \;10^{-1}$
50	$1.14\times 10^{1}$	$1.36\;\times \;10^{1}$	$1.27\;\times \;10^{1}$	$1.26\;\times \;10^{1}$	$4.95\;\times \;10^{-1}$
51	$1.00\times 10^{2}$	$4.00\;\times \;10^{2}$	$3.00\;\times \;10^{2}$	$3.10\;\times \;10^{2}$	$7.35\;\times \;10^{1}$
52	$1.01\times 10^{1}$	$2.43\;\times \;10^{2}$	$1.23\;\times \;10^{2}$	$1.14\;\times \;10^{2}$	$7.17\;\times \;10^{1}$
53	$3.26\times 10^{3}$	$6.95\;\times \;10^{3}$	$5.50\;\times \;10^{3}$	$5.45\;\times \;10^{3}$	$8.83\;\times \;10^{2}$
54	$2.75\times 10^{2}$	$3.30\;\times \;10^{2}$	$3.01\;\times \;10^{2}$	$3.02\;\times \;10^{2}$	$1.13\;\times \;10^{1}$
55	$3.13\times 10^{2}$	$3.71\;\times \;10^{2}$	$3.34\;\times \;10^{2}$	$3.33\;\times \;10^{2}$	$1.31\;\times \;10^{1}$
56	$2.00\times 10^{2}$	$4.11\;\times \;10^{2}$	$3.78\;\times \;10^{2}$	$3.35\;\times \;10^{2}$	$8.13\;\times \;10^{1}$
57	$1.02\times 10^{3}$	$1.45\;\times \;10^{3}$	$1.18\;\times \;10^{3}$	$1.19\;\times \;10^{3}$	$8.72\;\times \;10^{1}$
58	$1.00\times 10^{2}$	$3.20\;\times \;10^{3}$	$3.00\;\times \;10^{2}$	$7.84\;\times \;10^{2}$	$8.74\;\times \;10^{2}$
61	$3.50\times 10^{-17}$	$1.68\;\times \;10^{-14}$	$6.39\;\times \;10^{-16}$	$1.39\;\times \;10^{-15}$	$2.69\;\times \;10^{-15}$
62	$7.55\times 10^{-15}$	$3.60\;\times \;10^{-14}$	$1.47\;\times \;10^{-14}$	$1.66\;\times \;10^{-14}$	$5.61\;\times \;10^{-15}$
63	$-1.40\times 10^{2}$	$-1.40\;\times \;10^{2}$	$-1.40\;\times \;10^{2}$	$-1.40\;\times \;10^{2}$	$2.84\;\times \;10^{-14}$
64	2.12	6.94	3.49	3.62	$9.60\;\times \;10^{-1}$
65	$-1.03\times 10^{13}$	$-5.03\;\times \;10^{11}$	$-2.28\;\times \;10^{12}$	$-2.82\;\times \;10^{12}$	$1.89\;\times \;10^{12}$
66	$-2.27\times 10^{4}$	$-1.76\;\times \;10^{4}$	$-2.04\;\times \;10^{4}$	$-2.04\;\times \;10^{4}$	$9.52\;\times \;10^{2}$
67	$2.25\times 10^{-31}$	$1.31\;\times \;10^{-27}$	$1.77\;\times \;10^{-29}$	$1.37\;\times \;10^{-28}$	$2.71\;\times \;10^{-28}$
68	$-4.50\times 10^{2}$	$-4.50\;\times \;10^{2}$	$-4.50\;\times \;10^{2}$	$-4.50\;\times \;10^{2}$	$3.81\;\times \;10^{-14}$
69	0.00	$1.35\;\times \;10^{-1}$	$9.86\;\times \;10^{-3}$	$1.78\;\times \;10^{-2}$	$2.56\;\times \;10^{-2}$
70	$-1.80\times 10^{2}$	$-1.80\;\times \;10^{2}$	$-1.80\;\times \;10^{2}$	$-1.80\;\times \;10^{2}$	$1.65\;\times \;10^{-2}$
71	0.00	$5.90\;\times \;10^{-2}$	$9.86\;\times \;10^{-3}$	$1.30\;\times \;10^{-2}$	$1.26\;\times \;10^{-2}$
72	$4.24\times 10^{-35}$	$4.38\;\times \;10^{-32}$	$9.65\;\times \;10^{-34}$	$4.78\;\times \;10^{-33}$	$8.80\;\times \;10^{-33}$
73	$-4.50\times 10^{2}$	$-4.50\;\times \;10^{2}$	$-4.50\;\times \;10^{2}$	$-4.50\;\times \;10^{2}$	$5.39\;\times \;10^{-14}$
74	$-2.96\times 10^{1}$	$-2.95\;\times \;10^{1}$	$-2.96\;\times \;10^{1}$	$-2.96\;\times \;10^{1}$	$4.26\;\times \;10^{-2}$
75	$-8.53\times 10^{-1}$	$-7.82\;\times \;10^{-1}$	$-8.10\;\times \;10^{-1}$	$-8.09\;\times \;10^{-1}$	$1.72\;\times \;10^{-2}$
76	$9.00\times 10^{-15}$	$2.32\;\times \;10^{-12}$	$8.44\;\times \;10^{-14}$	$2.62\;\times \;10^{-13}$	$4.83\;\times \;10^{-13}$
77	$2.84\times 10^{-69}$	$3.44\;\times \;10^{-62}$	$3.78\;\times \;10^{-66}$	$1.03\;\times \;10^{-63}$	$4.96\;\times \;10^{-63}$
78	−3.49	$-3.52\;\times \;10^{-1}$	−2.42	−2.29	$7.50\;\times \;10^{-1}$
79	0.00	0.00	0.00	0.00	0.00
80	$6.57\times 10^{1}$	$2.05\;\times \;10^{2}$	$1.18\;\times \;10^{2}$	$1.21\;\times \;10^{2}$	$3.36\;\times \;10^{1}$
81	$1.57\times 10^{-6}$	7.44	$3.33\;\times \;10^{-3}$	$1.86\;\times \;10^{-1}$	1.06
82	9.01	$1.12\;\times \;10^{4}$	$1.92\;\times \;10^{1}$	$4.18\;\times \;10^{2}$	$1.71\;\times \;10^{3}$
83	$1.73\times 10^{-1}$	$2.45\;\times \;10^{-1}$	$2.00\;\times \;10^{-1}$	$2.03\;\times \;10^{-1}$	$2.12\;\times \;10^{-2}$
84	$2.72\times 10^{-1}$	1.63	$4.75\;\times \;10^{-1}$	$6.15\;\times \;10^{-1}$	$3.47\;\times \;10^{-1}$
85	$1.89\times 10^{-15}$	$6.65\;\times \;10^{-12}$	$9.23\;\times \;10^{-14}$	$3.85\;\times \;10^{-13}$	$1.09\;\times \;10^{-12}$
86	$7.54\times 10^{-15}$	$2.32\;\times \;10^{-11}$	$1.92\;\times \;10^{-12}$	$4.66\;\times \;10^{-12}$	$6.39\;\times \;10^{-12}$
87	$2.36\times 10^{3}$	$6.78\;\times \;10^{3}$	$4.04\;\times \;10^{3}$	$4.21\;\times \;10^{3}$	$1.16\;\times \;10^{3}$
88	$4.21\times 10^{-3}$	$1.44\;\times \;10^{4}$	$1.41\;\times \;10^{3}$	$2.93\;\times \;10^{3}$	$3.44\;\times \;10^{3}$
89	$2.61\times 10^{-5}$	$1.23\;\times \;10^{-3}$	$1.31\;\times \;10^{-4}$	$1.94\;\times \;10^{-4}$	$2.03\;\times \;10^{-4}$
90	$1.14\times 10^{-13}$	$1.71\;\times \;10^{-13}$	$1.14\;\times \;10^{-13}$	$1.32\;\times \;10^{-13}$	$2.68\;\times \;10^{-14}$
91	$-4.50\times 10^{2}$	$-3.24\;\times \;10^{2}$	$-4.48\;\times \;10^{2}$	$-4.45\;\times \;10^{2}$	$1.78\;\times \;10^{1}$
92	$-3.87\times 10^{34}$	$-1.17\;\times \;10^{34}$	$-1.89\;\times \;10^{34}$	$-2.15\;\times \;10^{34}$	$5.82\;\times \;10^{33}$
93	0.00	0.00	0.00	0.00	0.00
94	0.00	0.00	0.00	0.00	0.00
95	0.00	$1.42\;\times \;10^{-14}$	0.00	$2.84\;\times \;10^{-16}$	$2.01\;\times \;10^{-15}$

**Table A5:** Error values for uDFO${}_{\Delta_{\mathrm{dynmaic}}=1/1500p}$.

F No.	Min	Max	Median	Mean	StdDev
2	$5.68\times 10^{-14}$	$3.41\;\times \;10^{-13}$	$1.14\;\times \;10^{-13}$	$1.23\;\times \;10^{-13}$	$6.63\;\times \;10^{-14}$
4	$1.97\times 10^{2}$	$6.93\;\times \;10^{3}$	$2.24\;\times \;10^{3}$	$2.66\;\times \;10^{3}$	$1.86\;\times \;10^{3}$
5	$2.22\times 10^{3}$	$6.57\;\times \;10^{3}$	$4.12\;\times \;10^{3}$	$4.16\;\times \;10^{3}$	$1.11\;\times \;10^{3}$
6	$3.07\times 10^{-9}$	8.06	$7.11\;\times \;10^{-4}$	$3.73\;\times \;10^{-1}$	1.39
7	$4.70\times 10^{3}$	$4.70\;\times \;10^{3}$	$4.70\;\times \;10^{3}$	$4.70\;\times \;10^{3}$	$1.28\;\times \;10^{-12}$
8	$2.00\times 10^{1}$	$2.01\;\times \;10^{1}$	$2.00\;\times \;10^{1}$	$2.00\;\times \;10^{1}$	$1.27\;\times \;10^{-2}$
11	$1.97\times 10^{1}$	$3.99\;\times \;10^{1}$	$2.86\;\times \;10^{1}$	$2.95\;\times \;10^{1}$	4.04
12	$1.14\times 10^{1}$	$2.57\;\times \;10^{4}$	$2.72\;\times \;10^{3}$	$4.29\;\times \;10^{3}$	$5.35\;\times \;10^{3}$
13	1.12	2.82	1.73	1.77	$4.11\;\times \;10^{-1}$
14	$1.22\times 10^{1}$	$1.40\;\times \;10^{1}$	$1.35\;\times \;10^{1}$	$1.35\;\times \;10^{1}$	$3.88\;\times \;10^{-1}$
15	$2.00\times 10^{2}$	$5.04\;\times \;10^{2}$	$3.00\;\times \;10^{2}$	$3.19\;\times \;10^{2}$	$1.05\;\times \;10^{2}$
16	$1.77\times 10^{2}$	$8.00\;\times \;10^{2}$	$3.39\;\times \;10^{2}$	$3.41\;\times \;10^{2}$	$1.14\;\times \;10^{2}$
17	$1.75\times 10^{2}$	$8.05\;\times \;10^{2}$	$4.06\;\times \;10^{2}$	$3.96\;\times \;10^{2}$	$1.12\;\times \;10^{2}$
18	$9.29\times 10^{2}$	$1.22\;\times \;10^{3}$	$9.76\;\times \;10^{2}$	$9.98\;\times \;10^{2}$	$6.80\;\times \;10^{1}$
19	$9.20\times 10^{2}$	$1.23\;\times \;10^{3}$	$1.00\;\times \;10^{3}$	$1.02\;\times \;10^{3}$	$7.67\;\times \;10^{1}$
20	$8.00\times 10^{2}$	$1.10\;\times \;10^{3}$	$9.73\;\times \;10^{2}$	$9.83\;\times \;10^{2}$	$5.48\;\times \;10^{1}$
21	$5.00\times 10^{2}$	$1.23\;\times \;10^{3}$	$8.07\;\times \;10^{2}$	$8.30\;\times \;10^{2}$	$3.34\;\times \;10^{2}$
22	$9.92\times 10^{2}$	$1.37\;\times \;10^{3}$	$1.15\;\times \;10^{3}$	$1.17\;\times \;10^{3}$	$9.07\;\times \;10^{1}$
23	$5.00\times 10^{2}$	$1.23\;\times \;10^{3}$	$5.00\;\times \;10^{2}$	$7.91\;\times \;10^{2}$	$3.31\;\times \;10^{2}$
24	$2.00\times 10^{2}$	$1.36\;\times \;10^{3}$	$1.08\;\times \;10^{3}$	$7.41\;\times \;10^{2}$	$5.28\;\times \;10^{2}$
25	$1.67\times 10^{3}$	$1.80\;\times \;10^{3}$	$1.74\;\times \;10^{3}$	$1.74\;\times \;10^{3}$	$2.82\;\times \;10^{1}$
31	0.00	$2.27\;\times \;10^{-13}$	0.00	$1.36\;\times \;10^{-14}$	$5.45\;\times \;10^{-14}$
32	$3.03\times 10^{4}$	$3.65\;\times \;10^{5}$	$1.47\;\times \;10^{5}$	$1.65\;\times \;10^{5}$	$7.88\;\times \;10^{4}$
33	$4.30\times 10^{5}$	$1.33\;\times \;10^{9}$	$1.04\;\times \;10^{8}$	$1.60\;\times \;10^{8}$	$2.35\;\times \;10^{8}$
34	5.09	$5.06\;\times \;10^{2}$	$5.67\;\times \;10^{1}$	$8.67\;\times \;10^{1}$	$9.30\;\times \;10^{1}$
35	0.00	$1.14\;\times \;10^{-13}$	$1.14\;\times \;10^{-13}$	$6.14\;\times \;10^{-14}$	$5.72\;\times \;10^{-14}$
36	$3.19\times 10^{-2}$	$8.47\;\times \;10^{1}$	9.89	$1.71\;\times \;10^{1}$	$2.00\;\times \;10^{1}$
37	$5.06\times 10^{1}$	$2.14\;\times \;10^{2}$	$1.34\;\times \;10^{2}$	$1.36\;\times \;10^{2}$	$3.85\;\times \;10^{1}$
38	$2.07\times 10^{1}$	$2.11\;\times \;10^{1}$	$2.09\;\times \;10^{1}$	$2.09\;\times \;10^{1}$	$6.77\;\times \;10^{-2}$
39	$2.28\times 10^{1}$	$4.01\;\times \;10^{1}$	$3.10\;\times \;10^{1}$	$3.10\;\times \;10^{1}$	4.32
40	$1.48\times 10^{-2}$	$4.36\;\times \;10^{-1}$	$5.79\;\times \;10^{-2}$	$7.45\;\times \;10^{-2}$	$6.52\;\times \;10^{-2}$
41	0.00	$9.95\;\times \;10^{-1}$	0.00	$5.97\;\times \;10^{-2}$	$2.39\;\times \;10^{-1}$
42	$1.16\times 10^{2}$	$4.40\;\times \;10^{2}$	$2.42\;\times \;10^{2}$	$2.51\;\times \;10^{2}$	$8.30\;\times \;10^{1}$
43	$2.02\times 10^{2}$	$5.96\;\times \;10^{2}$	$3.42\;\times \;10^{2}$	$3.46\;\times \;10^{2}$	$8.61\;\times \;10^{1}$
44	1.44	$1.33\;\times \;10^{2}$	$1.27\;\times \;10^{1}$	$1.86\;\times \;10^{1}$	$2.43\;\times \;10^{1}$
45	$3.07\times 10^{3}$	$5.95\;\times \;10^{3}$	$4.52\;\times \;10^{3}$	$4.47\;\times \;10^{3}$	$7.06\;\times \;10^{2}$
46	$2.44\times 10^{-1}$	2.56	1.06	1.21	$6.08\;\times \;10^{-1}$
47	$3.05\times 10^{1}$	$3.19\;\times \;10^{1}$	$3.09\;\times \;10^{1}$	$3.09\;\times \;10^{1}$	$2.90\;\times \;10^{-1}$
48	$3.00\times 10^{1}$	$3.09\;\times \;10^{1}$	$3.00\;\times \;10^{1}$	$3.01\;\times \;10^{1}$	$2.04\;\times \;10^{-1}$
49	$9.90\times 10^{-1}$	3.37	2.04	2.06	$5.29\;\times \;10^{-1}$
50	$1.13\times 10^{1}$	$1.36\;\times \;10^{1}$	$1.27\;\times \;10^{1}$	$1.27\;\times \;10^{1}$	$5.74\;\times \;10^{-1}$
51	$1.00\times 10^{2}$	$4.00\;\times \;10^{2}$	$3.00\;\times \;10^{2}$	$3.04\;\times \;10^{2}$	$6.69\;\times \;10^{1}$
52	$1.64\times 10^{1}$	$2.57\;\times \;10^{2}$	$1.33\;\times \;10^{2}$	$1.47\;\times \;10^{2}$	$7.41\;\times \;10^{1}$
53	$3.23\times 10^{3}$	$7.82\;\times \;10^{3}$	$5.72\;\times \;10^{3}$	$5.58\;\times \;10^{3}$	$9.93\;\times \;10^{2}$
54	$2.71\times 10^{2}$	$3.33\;\times \;10^{2}$	$3.01\;\times \;10^{2}$	$3.03\;\times \;10^{2}$	$1.33\;\times \;10^{1}$
55	$2.92\times 10^{2}$	$3.60\;\times \;10^{2}$	$3.24\;\times \;10^{2}$	$3.25\;\times \;10^{2}$	$1.44\;\times \;10^{1}$
56	$2.00\times 10^{2}$	$4.05\;\times \;10^{2}$	$3.80\;\times \;10^{2}$	$3.42\;\times \;10^{2}$	$7.67\;\times \;10^{1}$
57	$9.24\times 10^{2}$	$1.41\;\times \;10^{3}$	$1.17\;\times \;10^{3}$	$1.17\;\times \;10^{3}$	$1.06\;\times \;10^{2}$
58	$1.00\times 10^{2}$	$3.22\;\times \;10^{3}$	$3.00\;\times \;10^{2}$	$8.97\;\times \;10^{2}$	$9.47\;\times \;10^{2}$
61	$1.05\times 10^{-20}$	$1.73\;\times \;10^{-17}$	$2.50\;\times \;10^{-19}$	$1.09\;\times \;10^{-18}$	$2.73\;\times \;10^{-18}$
62	$4.00\times 10^{-15}$	$1.47\;\times \;10^{-14}$	$7.55\;\times \;10^{-15}$	$7.76\;\times \;10^{-15}$	$1.51\;\times \;10^{-15}$
63	$-1.40\times 10^{2}$	$-1.40\;\times \;10^{2}$	$-1.40\;\times \;10^{2}$	$-1.40\;\times \;10^{2}$	$1.46\;\times \;10^{-14}$
64	1.34	6.23	3.60	3.68	1.08
65	$-6.25\times 10^{12}$	$-5.03\;\times \;10^{11}$	$-1.38\;\times \;10^{12}$	$-1.95\;\times \;10^{12}$	$1.26\;\times \;10^{12}$
66	$-2.23\times 10^{4}$	$-1.86\;\times \;10^{4}$	$-2.05\;\times \;10^{4}$	$-2.05\;\times \;10^{4}$	$8.91\;\times \;10^{2}$
67	$2.83\times 10^{-41}$	$6.96\;\times \;10^{-36}$	$5.64\;\times \;10^{-38}$	$4.97\;\times \;10^{-37}$	$1.20\;\times \;10^{-36}$
68	$-4.50\times 10^{2}$	$-4.50\;\times \;10^{2}$	$-4.50\;\times \;10^{2}$	$-4.50\;\times \;10^{2}$	$1.15\;\times \;10^{-14}$
69	0.00	$1.05\;\times \;10^{-1}$	$1.23\;\times \;10^{-2}$	$2.21\;\times \;10^{-2}$	$2.78\;\times \;10^{-2}$
70	$-1.80\times 10^{2}$	$-1.80\;\times \;10^{2}$	$-1.80\;\times \;10^{2}$	$-1.80\;\times \;10^{2}$	$1.73\;\times \;10^{-2}$
71	0.00	$5.13\;\times \;10^{-2}$	$9.86\;\times \;10^{-3}$	$1.21\;\times \;10^{-2}$	$1.26\;\times \;10^{-2}$
72	$9.77\times 10^{-45}$	$1.86\;\times \;10^{-38}$	$4.45\;\times \;10^{-42}$	$7.39\;\times \;10^{-40}$	$3.48\;\times \;10^{-39}$
73	$-4.50\times 10^{2}$	$-4.50\;\times \;10^{2}$	$-4.50\;\times \;10^{2}$	$-4.50\;\times \;10^{2}$	$5.86\;\times \;10^{-14}$
74	$-2.96\times 10^{1}$	$-2.93\;\times \;10^{1}$	$-2.95\;\times \;10^{1}$	$-2.95\;\times \;10^{1}$	$6.20\;\times \;10^{-2}$
75	$-8.68\times 10^{-1}$	$-7.69\;\times \;10^{-1}$	$-7.98\;\times \;10^{-1}$	$-8.06\;\times \;10^{-1}$	$1.94\;\times \;10^{-2}$
76	$5.28\times 10^{-17}$	$1.48\;\times \;10^{-13}$	$4.55\;\times \;10^{-15}$	$1.70\;\times \;10^{-14}$	$3.20\;\times \;10^{-14}$
77	$3.57\times 10^{-86}$	$3.00\;\times \;10^{-76}$	$9.68\;\times \;10^{-82}$	$6.71\;\times \;10^{-78}$	$4.24\;\times \;10^{-77}$
78	−4.00	$-1.79\;\times \;10^{-1}$	−2.25	−2.28	$8.43\;\times \;10^{-1}$
79	0.00	$9.95\;\times \;10^{-1}$	0.00	$3.98\;\times \;10^{-2}$	$1.97\;\times \;10^{-1}$
80	$6.87\times 10^{1}$	$2.45\;\times \;10^{2}$	$1.21\;\times \;10^{2}$	$1.29\;\times \;10^{2}$	$3.72\;\times \;10^{1}$
81	$8.61\times 10^{-8}$	4.18	$6.39\;\times \;10^{-4}$	$1.80\;\times \;10^{-1}$	$8.11\;\times \;10^{-1}$
82	$4.69\times 10^{-1}$	$9.58\;\times \;10^{3}$	$1.84\;\times \;10^{1}$	$6.54\;\times \;10^{2}$	$1.77\;\times \;10^{3}$
83	$1.41\times 10^{-1}$	$2.45\;\times \;10^{-1}$	$2.12\;\times \;10^{-1}$	$2.09\;\times \;10^{-1}$	$2.48\;\times \;10^{-2}$
84	$1.36\times 10^{-1}$	1.46	$7.04\;\times \;10^{-1}$	$6.44\;\times \;10^{-1}$	$3.56\;\times \;10^{-1}$
85	$1.14\times 10^{-17}$	$2.44\;\times \;10^{-11}$	$5.61\;\times \;10^{-15}$	$5.00\;\times \;10^{-13}$	$3.44\;\times \;10^{-12}$
86	$1.49\times 10^{-15}$	$6.70\;\times \;10^{-12}$	$8.00\;\times \;10^{-14}$	$4.23\;\times \;10^{-13}$	$1.06\;\times \;10^{-12}$
87	$1.72\times 10^{3}$	$8.27\;\times \;10^{3}$	$4.30\;\times \;10^{3}$	$4.28\;\times \;10^{3}$	$1.23\;\times \;10^{3}$
88	$8.26\times 10^{1}$	$2.52\;\times \;10^{4}$	$2.96\;\times \;10^{3}$	$3.96\;\times \;10^{3}$	$4.40\;\times \;10^{3}$
89	$5.91\times 10^{-6}$	$5.54\;\times \;10^{-4}$	$4.73\;\times \;10^{-5}$	$9.84\;\times \;10^{-5}$	$1.31\;\times \;10^{-4}$
90	$5.68\times 10^{-14}$	$1.14\;\times \;10^{-13}$	$8.53\;\times \;10^{-14}$	$8.53\;\times \;10^{-14}$	$2.87\;\times \;10^{-14}$
91	$-4.50\times 10^{2}$	$-3.08\;\times \;10^{2}$	$-4.48\;\times \;10^{2}$	$-4.42\;\times \;10^{2}$	$2.56\;\times \;10^{1}$
92	$-3.05\times 10^{34}$	$-1.17\;\times \;10^{34}$	$-1.89\;\times \;10^{34}$	$-1.92\;\times \;10^{34}$	$4.75\;\times \;10^{33}$
93	0.00	0.00	0.00	0.00	0.00
94	0.00	0.00	0.00	0.00	0.00
95	0.00	0.00	0.00	0.00	0.00

**Table A6:** Error values for uDFO${}_{z5}$.

F No.	Min	Max	Median	Mean	StdDev
2	$5.68\times 10^{-14}$	$1.08\;\times \;10^{-12}$	$1.14\;\times \;10^{-13}$	$1.44\;\times \;10^{-13}$	$1.81\;\times \;10^{-13}$
4	$1.90\times 10^{2}$	$1.58\;\times \;10^{4}$	$2.21\;\times \;10^{3}$	$3.23\;\times \;10^{3}$	$3.07\;\times \;10^{3}$
5	$2.27\times 10^{3}$	$5.92\;\times \;10^{3}$	$4.27\;\times \;10^{3}$	$4.15\;\times \;10^{3}$	$8.70\;\times \;10^{2}$
6	$2.45\times 10^{-7}$	$1.77\;\times \;10^{1}$	$1.03\;\times \;10^{-3}$	$5.83\;\times \;10^{-1}$	2.61
7	$4.70\times 10^{3}$	$4.70\;\times \;10^{3}$	$4.70\;\times \;10^{3}$	$4.70\;\times \;10^{3}$	$1.26\;\times \;10^{-12}$
8	$2.00\times 10^{1}$	$2.00\;\times \;10^{1}$	$2.00\;\times \;10^{1}$	$2.00\;\times \;10^{1}$	$8.67\;\times \;10^{-3}$
11	$2.21\times 10^{1}$	$3.72\;\times \;10^{1}$	$2.98\;\times \;10^{1}$	$2.93\;\times \;10^{1}$	3.78
12	9.30	$2.99\;\times \;10^{4}$	$3.31\;\times \;10^{3}$	$6.59\;\times \;10^{3}$	$7.56\;\times \;10^{3}$
13	1.11	2.67	1.72	1.77	$3.88\;\times \;10^{-1}$
14	$1.22\times 10^{1}$	$1.40\;\times \;10^{1}$	$1.36\;\times \;10^{1}$	$1.35\;\times \;10^{1}$	$4.03\;\times \;10^{-1}$
15	6.29	$5.13\;\times \;10^{2}$	$3.00\;\times \;10^{2}$	$3.08\;\times \;10^{2}$	$1.43\;\times \;10^{2}$
16	$1.67\times 10^{2}$	$6.16\;\times \;10^{2}$	$3.81\;\times \;10^{2}$	$3.69\;\times \;10^{2}$	$1.07\;\times \;10^{2}$
17	$1.67\times 10^{2}$	$9.08\;\times \;10^{2}$	$3.85\;\times \;10^{2}$	$4.06\;\times \;10^{2}$	$1.23\;\times \;10^{2}$
18	$9.29\times 10^{2}$	$1.26\;\times \;10^{3}$	$9.65\;\times \;10^{2}$	$9.87\;\times \;10^{2}$	$6.76\;\times \;10^{1}$
19	$9.23\times 10^{2}$	$1.17\;\times \;10^{3}$	$9.76\;\times \;10^{2}$	$9.93\;\times \;10^{2}$	$6.05\;\times \;10^{1}$
20	$9.31\times 10^{2}$	$1.15\;\times \;10^{3}$	$9.85\;\times \;10^{2}$	$1.00\;\times \;10^{3}$	$5.70\;\times \;10^{1}$
21	$5.00\times 10^{2}$	$1.25\;\times \;10^{3}$	$8.07\;\times \;10^{2}$	$8.38\;\times \;10^{2}$	$3.39\;\times \;10^{2}$
22	$9.75\times 10^{2}$	$1.34\;\times \;10^{3}$	$1.14\;\times \;10^{3}$	$1.14\;\times \;10^{3}$	$7.94\;\times \;10^{1}$
23	$5.00\times 10^{2}$	$1.24\;\times \;10^{3}$	$8.16\;\times \;10^{2}$	$8.48\;\times \;10^{2}$	$3.40\;\times \;10^{2}$
24	$2.00\times 10^{2}$	$1.35\;\times \;10^{3}$	$2.00\;\times \;10^{2}$	$6.66\;\times \;10^{2}$	$5.22\;\times \;10^{2}$
25	$1.70\times 10^{3}$	$1.89\;\times \;10^{3}$	$1.76\;\times \;10^{3}$	$1.77\;\times \;10^{3}$	$3.88\;\times \;10^{1}$
31	0.00	0.00	0.00	0.00	0.00
32	$5.35\times 10^{4}$	$2.91\;\times \;10^{5}$	$1.51\;\times \;10^{5}$	$1.54\;\times \;10^{5}$	$5.35\;\times \;10^{4}$
33	$5.72\times 10^{5}$	$1.36\;\times \;10^{9}$	$7.98\;\times \;10^{7}$	$1.73\;\times \;10^{8}$	$2.42\;\times \;10^{8}$
34	9.83	$7.60\;\times \;10^{2}$	$4.07\;\times \;10^{1}$	$7.83\;\times \;10^{1}$	$1.18\;\times \;10^{2}$
35	0.00	$1.14\;\times \;10^{-13}$	$1.14\;\times \;10^{-13}$	$8.19\;\times \;10^{-14}$	$5.16\;\times \;10^{-14}$
36	$1.94\times 10^{-3}$	$8.47\;\times \;10^{1}$	9.99	$1.39\;\times \;10^{1}$	$1.87\;\times \;10^{1}$
37	$5.87\times 10^{1}$	$2.47\;\times \;10^{2}$	$1.35\;\times \;10^{2}$	$1.43\;\times \;10^{2}$	$4.41\;\times \;10^{1}$
38	$2.08\times 10^{1}$	$2.10\;\times \;10^{1}$	$2.09\;\times \;10^{1}$	$2.09\;\times \;10^{1}$	$6.06\;\times \;10^{-2}$
39	$2.09\times 10^{1}$	$4.27\;\times \;10^{1}$	$3.30\;\times \;10^{1}$	$3.26\;\times \;10^{1}$	5.21
40	$1.23\times 10^{-2}$	$2.63\;\times \;10^{-1}$	$5.42\;\times \;10^{-2}$	$7.05\;\times \;10^{-2}$	$5.04\;\times \;10^{-2}$
41	0.00	3.98	$5.68\;\times \;10^{-14}$	$3.38\;\times \;10^{-1}$	$7.14\;\times \;10^{-1}$
42	$9.45\times 10^{1}$	$5.20\;\times \;10^{2}$	$2.32\;\times \;10^{2}$	$2.41\;\times \;10^{2}$	$8.09\;\times \;10^{1}$
43	$2.62\times 10^{2}$	$6.12\;\times \;10^{2}$	$3.51\;\times \;10^{2}$	$3.67\;\times \;10^{2}$	$7.98\;\times \;10^{1}$
44	$1.25\times 10^{1}$	$4.74\;\times \;10^{2}$	$7.34\;\times \;10^{1}$	$1.09\;\times \;10^{2}$	$1.03\;\times \;10^{2}$
45	$2.72\times 10^{3}$	$6.22\;\times \;10^{3}$	$4.48\;\times \;10^{3}$	$4.47\;\times \;10^{3}$	$7.96\;\times \;10^{2}$
46	$3.15\times 10^{-1}$	2.94	$9.91\;\times \;10^{-1}$	1.16	$5.58\;\times \;10^{-1}$
47	$3.05\times 10^{1}$	$3.14\;\times \;10^{1}$	$3.08\;\times \;10^{1}$	$3.08\;\times \;10^{1}$	$2.38\;\times \;10^{-1}$
48	$3.00\times 10^{1}$	$3.09\;\times \;10^{1}$	$3.00\;\times \;10^{1}$	$3.01\;\times \;10^{1}$	$2.70\;\times \;10^{-1}$
49	$4.15\times 10^{-1}$	3.27	1.66	1.72	$5.68\;\times \;10^{-1}$
50	$1.15\times 10^{1}$	$1.39\;\times \;10^{1}$	$1.27\;\times \;10^{1}$	$1.27\;\times \;10^{1}$	$5.88\;\times \;10^{-1}$
51	$2.00\times 10^{2}$	$4.00\;\times \;10^{2}$	$3.00\;\times \;10^{2}$	$3.08\;\times \;10^{2}$	$6.65\;\times \;10^{1}$
52	$3.10\times 10^{1}$	$6.27\;\times \;10^{2}$	$1.81\;\times \;10^{2}$	$2.22\;\times \;10^{2}$	$1.17\;\times \;10^{2}$
53	$4.05\times 10^{3}$	$7.84\;\times \;10^{3}$	$5.43\;\times \;10^{3}$	$5.56\;\times \;10^{3}$	$9.55\;\times \;10^{2}$
54	$2.70\times 10^{2}$	$3.31\;\times \;10^{2}$	$3.03\;\times \;10^{2}$	$2.99\;\times \;10^{2}$	$1.58\;\times \;10^{1}$
55	$3.00\times 10^{2}$	$3.57\;\times \;10^{2}$	$3.28\;\times \;10^{2}$	$3.30\;\times \;10^{2}$	$1.40\;\times \;10^{1}$
56	$2.00\times 10^{2}$	$3.98\;\times \;10^{2}$	$3.76\;\times \;10^{2}$	$3.27\;\times \;10^{2}$	$8.46\;\times \;10^{1}$
57	$9.81\times 10^{2}$	$1.41\;\times \;10^{3}$	$1.18\;\times \;10^{3}$	$1.19\;\times \;10^{3}$	$1.05\;\times \;10^{2}$
58	$1.00\times 10^{2}$	$3.49\;\times \;10^{3}$	$3.00\;\times \;10^{2}$	$8.17\;\times \;10^{2}$	$9.09\;\times \;10^{2}$
61	$4.44\times 10^{-21}$	$1.34\;\times \;10^{-17}$	$1.93\;\times \;10^{-19}$	$6.36\;\times \;10^{-19}$	$1.95\;\times \;10^{-18}$
62	$4.00\times 10^{-15}$	$7.55\;\times \;10^{-15}$	$7.55\;\times \;10^{-15}$	$7.27\;\times \;10^{-15}$	$9.74\;\times \;10^{-16}$
63	$-1.40\times 10^{2}$	$-1.40\;\times \;10^{2}$	$-1.40\;\times \;10^{2}$	$-1.40\;\times \;10^{2}$	$1.57\;\times \;10^{-14}$
64	1.90	8.79	3.73	4.08	1.47
65	$-1.03\times 10^{13}$	$-3.04\;\times \;10^{11}$	$-1.38\;\times \;10^{12}$	$-2.06\;\times \;10^{12}$	$1.81\;\times \;10^{12}$
66	$-2.31\times 10^{4}$	$-1.84\;\times \;10^{4}$	$-2.05\;\times \;10^{4}$	$-2.05\;\times \;10^{4}$	$9.85\;\times \;10^{2}$
67	$3.76\times 10^{-40}$	$2.34\;\times \;10^{-35}$	$1.48\;\times \;10^{-37}$	$9.51\;\times \;10^{-37}$	$3.50\;\times \;10^{-36}$
68	$-4.50\times 10^{2}$	$-4.50\;\times \;10^{2}$	$-4.50\;\times \;10^{2}$	$-4.50\;\times \;10^{2}$	$1.41\;\times \;10^{-14}$
69	0.00	$6.61\;\times \;10^{-2}$	$7.40\;\times \;10^{-3}$	$1.45\;\times \;10^{-2}$	$1.95\;\times \;10^{-2}$
70	$-1.80\times 10^{2}$	$-1.80\;\times \;10^{2}$	$-1.80\;\times \;10^{2}$	$-1.80\;\times \;10^{2}$	$2.37\;\times \;10^{-2}$
71	0.00	$5.16\;\times \;10^{-2}$	$9.86\;\times \;10^{-3}$	$1.37\;\times \;10^{-2}$	$1.40\;\times \;10^{-2}$
72	$1.98\times 10^{-45}$	$3.87\;\times \;10^{-40}$	$2.05\;\times \;10^{-42}$	$2.57\;\times \;10^{-41}$	$6.42\;\times \;10^{-41}$
73	$-4.50\times 10^{2}$	$-4.50\;\times \;10^{2}$	$-4.50\;\times \;10^{2}$	$-4.50\;\times \;10^{2}$	$5.63\;\times \;10^{-14}$
74	$-2.96\times 10^{1}$	$-2.80\;\times \;10^{1}$	$-2.95\;\times \;10^{1}$	$-2.92\;\times \;10^{1}$	$4.44\;\times \;10^{-1}$
75	$-8.53\times 10^{-1}$	$-7.61\;\times \;10^{-1}$	$-8.07\;\times \;10^{-1}$	$-8.08\;\times \;10^{-1}$	$2.03\;\times \;10^{-2}$
76	$1.33\times 10^{-16}$	$6.31\;\times \;10^{-14}$	$3.77\;\times \;10^{-15}$	$8.03\;\times \;10^{-15}$	$1.12\;\times \;10^{-14}$
77	$5.35\times 10^{-85}$	$7.15\;\times \;10^{-77}$	$8.41\;\times \;10^{-82}$	$2.48\;\times \;10^{-78}$	$1.07\;\times \;10^{-77}$
78	−3.38	$5.43\;\times \;10^{-1}$	−2.20	−2.03	$9.37\;\times \;10^{-1}$
79	0.00	$2.49\;\times \;10^{1}$	0.00	1.49	5.97
80	$5.47\times 10^{1}$	$2.70\;\times \;10^{2}$	$1.24\;\times \;10^{2}$	$1.28\;\times \;10^{2}$	$4.34\;\times \;10^{1}$
81	$4.70\times 10^{-8}$	$2.31\;\times \;10^{-1}$	$3.91\;\times \;10^{-4}$	$8.22\;\times \;10^{-3}$	$3.35\;\times \;10^{-2}$
82	7.26	$1.27\;\times \;10^{4}$	$2.00\;\times \;10^{1}$	$6.46\;\times \;10^{2}$	$2.27\;\times \;10^{3}$
83	$1.73\times 10^{-1}$	$2.65\;\times \;10^{-1}$	$2.24\;\times \;10^{-1}$	$2.14\;\times \;10^{-1}$	$2.11\;\times \;10^{-2}$
84	$2.05\times 10^{-1}$	2.09	$3.57\;\times \;10^{-1}$	$6.36\;\times \;10^{-1}$	$4.89\;\times \;10^{-1}$
85	$1.11\times 10^{-16}$	$1.43\;\times \;10^{-13}$	$3.81\;\times \;10^{-15}$	$1.64\;\times \;10^{-14}$	$3.09\;\times \;10^{-14}$
86	$3.54\times 10^{-16}$	$5.60\;\times \;10^{-12}$	$7.21\;\times \;10^{-14}$	$3.85\;\times \;10^{-13}$	$9.08\;\times \;10^{-13}$
87	$1.58\times 10^{3}$	$6.18\;\times \;10^{3}$	$3.85\;\times \;10^{3}$	$3.90\;\times \;10^{3}$	$1.03\;\times \;10^{3}$
88	$2.40\times 10^{1}$	$4.19\;\times \;10^{4}$	$8.12\;\times \;10^{3}$	$9.33\;\times \;10^{3}$	$8.24\;\times \;10^{3}$
89	$6.00\times 10^{-6}$	$2.68\;\times \;10^{-4}$	$5.20\;\times \;10^{-5}$	$7.35\;\times \;10^{-5}$	$6.33\;\times \;10^{-5}$
90	$5.68\times 10^{-14}$	$1.14\;\times \;10^{-13}$	$1.14\;\times \;10^{-13}$	$9.21\;\times \;10^{-14}$	$2.79\;\times \;10^{-14}$
91	$-4.50\times 10^{2}$	$-3.28\;\times \;10^{2}$	$-4.48\;\times \;10^{2}$	$-4.44\;\times \;10^{2}$	$1.73\;\times \;10^{1}$
92	$-3.87\times 10^{34}$	$-9.21\;\times \;10^{33}$	$-1.89\;\times \;10^{34}$	$-2.04\;\times \;10^{34}$	$6.84\;\times \;10^{33}$
93	0.00	0.00	0.00	0.00	0.00
94	0.00	0.00	0.00	0.00	0.00
95	0.00	3.00	$9.59\;\times \;10^{-14}$	$3.30\;\times \;10^{-1}$	$7.60\;\times \;10^{-1}$

**Table A7:** Error values for GPSO.

F No.	Min	Max	Median	Mean	StdDev
2	$2.35\times 10^{-11}$	$2.12\;\times \;10^{-8}$	$9.42\;\times \;10^{-10}$	$2.26\;\times \;10^{-9}$	$4.00\;\times \;10^{-9}$
4	$5.55\times 10^{1}$	$1.03\;\times \;10^{4}$	$1.66\;\times \;10^{3}$	$2.22\;\times \;10^{3}$	$2.04\;\times \;10^{3}$
5	$3.63\times 10^{3}$	$9.85\;\times \;10^{3}$	$5.42\;\times \;10^{3}$	$5.52\;\times \;10^{3}$	$1.33\;\times \;10^{3}$
6	$3.41\times 10^{-2}$	$1.95\;\times \;10^{2}$	$1.09\;\times \;10^{1}$	$2.18\;\times \;10^{1}$	$4.11\;\times \;10^{1}$
7	$4.70\times 10^{3}$	$4.70\;\times \;10^{3}$	$4.70\;\times \;10^{3}$	$4.70\;\times \;10^{3}$	$7.33\;\times \;10^{-5}$
8	$2.07\times 10^{1}$	$2.10\;\times \;10^{1}$	$2.09\;\times \;10^{1}$	$2.09\;\times \;10^{1}$	$6.75\;\times \;10^{-2}$
11	$2.44\times 10^{1}$	$3.67\;\times \;10^{1}$	$3.13\;\times \;10^{1}$	$3.12\;\times \;10^{1}$	3.07
12	$2.10\times 10^{2}$	$1.33\;\times \;10^{5}$	$1.06\;\times \;10^{4}$	$1.83\;\times \;10^{4}$	$2.30\;\times \;10^{4}$
13	2.64	$1.29\;\times \;10^{1}$	5.39	5.72	2.08
14	$1.15\times 10^{1}$	$1.38\;\times \;10^{1}$	$1.28\;\times \;10^{1}$	$1.28\;\times \;10^{1}$	$4.85\;\times \;10^{-1}$
15	$2.30\times 10^{2}$	$5.86\;\times \;10^{2}$	$4.56\;\times \;10^{2}$	$4.46\;\times \;10^{2}$	$8.57\;\times \;10^{1}$
16	$1.73\times 10^{2}$	$9.00\;\times \;10^{2}$	$3.84\;\times \;10^{2}$	$3.59\;\times \;10^{2}$	$1.25\;\times \;10^{2}$
17	$1.55\times 10^{2}$	$9.13\;\times \;10^{2}$	$4.21\;\times \;10^{2}$	$3.89\;\times \;10^{2}$	$1.62\;\times \;10^{2}$
18	$9.23\times 10^{2}$	$1.18\;\times \;10^{3}$	$9.76\;\times \;10^{2}$	$9.87\;\times \;10^{2}$	$5.43\;\times \;10^{1}$
19	$9.34\times 10^{2}$	$1.08\;\times \;10^{3}$	$9.91\;\times \;10^{2}$	$9.94\;\times \;10^{2}$	$4.25\;\times \;10^{1}$
20	$9.27\times 10^{2}$	$1.10\;\times \;10^{3}$	$9.91\;\times \;10^{2}$	$1.00\;\times \;10^{3}$	$4.40\;\times \;10^{1}$
21	$5.00\times 10^{2}$	$1.24\;\times \;10^{3}$	$6.50\;\times \;10^{2}$	$8.16\;\times \;10^{2}$	$3.33\;\times \;10^{2}$
22	$9.80\times 10^{2}$	$1.27\;\times \;10^{3}$	$1.06\;\times \;10^{3}$	$1.07\;\times \;10^{3}$	$6.48\;\times \;10^{1}$
23	$5.00\times 10^{2}$	$1.21\;\times \;10^{3}$	$8.59\;\times \;10^{2}$	$8.41\;\times \;10^{2}$	$3.38\;\times \;10^{2}$
24	$2.00\times 10^{2}$	$1.33\;\times \;10^{3}$	$2.00\;\times \;10^{2}$	$4.71\;\times \;10^{2}$	$4.62\;\times \;10^{2}$
25	$1.68\times 10^{3}$	$1.76\;\times \;10^{3}$	$1.71\;\times \;10^{3}$	$1.71\;\times \;10^{3}$	$1.95\;\times \;10^{1}$
31	0.00	$2.27\;\times \;10^{-13}$	$2.27\;\times \;10^{-13}$	$1.82\;\times \;10^{-13}$	$9.19\;\times \;10^{-14}$
32	$2.70\times 10^{5}$	$1.70\;\times \;10^{6}$	$7.05\;\times \;10^{5}$	$7.57\;\times \;10^{5}$	$3.15\;\times \;10^{5}$
33	$1.18\times 10^{7}$	$2.41\;\times \;10^{9}$	$2.42\;\times \;10^{8}$	$4.06\;\times \;10^{8}$	$4.79\;\times \;10^{8}$
34	$1.33\times 10^{3}$	$1.61\;\times \;10^{4}$	$4.48\;\times \;10^{3}$	$5.31\;\times \;10^{3}$	$3.13\;\times \;10^{3}$
35	0.00	$1.14\;\times \;10^{-13}$	$1.14\;\times \;10^{-13}$	$1.09\;\times \;10^{-13}$	$2.25\;\times \;10^{-14}$
36	$5.45\times 10^{-2}$	$7.41\;\times \;10^{1}$	$1.34\;\times \;10^{1}$	$2.29\;\times \;10^{1}$	$2.26\;\times \;10^{1}$
37	$5.47\times 10^{1}$	$3.30\;\times \;10^{2}$	$1.36\;\times \;10^{2}$	$1.31\;\times \;10^{2}$	$4.91\;\times \;10^{1}$
38	$2.08\times 10^{1}$	$2.10\;\times \;10^{1}$	$2.09\;\times \;10^{1}$	$2.09\;\times \;10^{1}$	$6.18\;\times \;10^{-2}$
39	$2.28\times 10^{1}$	$4.07\;\times \;10^{1}$	$3.29\;\times \;10^{1}$	$3.26\;\times \;10^{1}$	3.60
40	$1.23\times 10^{-2}$	$3.54\;\times \;10^{-1}$	$1.03\;\times \;10^{-1}$	$1.16\;\times \;10^{-1}$	$6.51\;\times \;10^{-2}$
41	$4.58\times 10^{1}$	$1.78\;\times \;10^{2}$	$1.00\;\times \;10^{2}$	$1.02\;\times \;10^{2}$	$3.28\;\times \;10^{1}$
42	$9.15\times 10^{1}$	$5.57\;\times \;10^{2}$	$1.90\;\times \;10^{2}$	$2.01\;\times \;10^{2}$	$8.66\;\times \;10^{1}$
43	$1.71\times 10^{2}$	$3.59\;\times \;10^{2}$	$2.58\;\times \;10^{2}$	$2.53\;\times \;10^{2}$	$4.30\;\times \;10^{1}$
44	$1.48\times 10^{3}$	$3.37\;\times \;10^{3}$	$2.36\;\times \;10^{3}$	$2.34\;\times \;10^{3}$	$4.93\;\times \;10^{2}$
45	$3.29\times 10^{3}$	$5.99\;\times \;10^{3}$	$4.36\;\times \;10^{3}$	$4.45\;\times \;10^{3}$	$5.78\;\times \;10^{2}$
46	$5.79\times 10^{-1}$	2.97	1.51	1.51	$5.03\;\times \;10^{-1}$
47	$7.72\times 10^{1}$	$1.77\;\times \;10^{2}$	$1.26\;\times \;10^{2}$	$1.27\;\times \;10^{2}$	$2.78\;\times \;10^{1}$
48	$5.24\times 10^{1}$	$2.39\;\times \;10^{2}$	$1.32\;\times \;10^{2}$	$1.38\;\times \;10^{2}$	$4.00\;\times \;10^{1}$
49	2.65	$1.66\;\times \;10^{1}$	7.76	8.21	3.02
50	$1.07\times 10^{1}$	$1.32\;\times \;10^{1}$	$1.20\;\times \;10^{1}$	$1.20\;\times \;10^{1}$	$6.17\;\times \;10^{-1}$
51	$1.00\times 10^{2}$	$4.00\;\times \;10^{2}$	$3.00\;\times \;10^{2}$	$3.10\;\times \;10^{2}$	$8.63\;\times \;10^{1}$
52	$1.37\times 10^{3}$	$3.78\;\times \;10^{3}$	$2.75\;\times \;10^{3}$	$2.68\;\times \;10^{3}$	$5.59\;\times \;10^{2}$
53	$2.85\times 10^{3}$	$7.83\;\times \;10^{3}$	$5.52\;\times \;10^{3}$	$5.63\;\times \;10^{3}$	$9.36\;\times \;10^{2}$
54	$2.71\times 10^{2}$	$3.24\;\times \;10^{2}$	$2.99\;\times \;10^{2}$	$2.97\;\times \;10^{2}$	$1.37\;\times \;10^{1}$
55	$2.91\times 10^{2}$	$3.59\;\times \;10^{2}$	$3.26\;\times \;10^{2}$	$3.26\;\times \;10^{2}$	$1.23\;\times \;10^{1}$
56	$2.00\times 10^{2}$	$4.00\;\times \;10^{2}$	$3.85\;\times \;10^{2}$	$3.63\;\times \;10^{2}$	$6.14\;\times \;10^{1}$
57	$1.01\times 10^{3}$	$1.38\;\times \;10^{3}$	$1.21\;\times \;10^{3}$	$1.20\;\times \;10^{3}$	$8.72\;\times \;10^{1}$
58	$1.00\times 10^{2}$	$2.97\;\times \;10^{3}$	$3.00\;\times \;10^{2}$	$7.81\;\times \;10^{2}$	$8.85\;\times \;10^{2}$
61	$1.78\times 10^{-39}$	$7.00\;\times \;10^{-28}$	$6.72\;\times \;10^{-34}$	$2.99\;\times \;10^{-29}$	$1.27\;\times \;10^{-28}$
62	$7.55\times 10^{-15}$	5.41	1.50	1.65	1.13
63	$-1.40\times 10^{2}$	$-1.35\;\times \;10^{2}$	$-1.38\;\times \;10^{2}$	$-1.38\;\times \;10^{2}$	$8.64\;\times \;10^{-1}$
64	$7.55\times 10^{-15}$	5.13	2.41	2.56	1.08
65	$-1.38\times 10^{12}$	$-8.50\;\times \;10^{9}$	$-1.39\;\times \;10^{11}$	$-2.46\;\times \;10^{11}$	$2.87\;\times \;10^{11}$
66	$-1.56\times 10^{4}$	$-1.06\;\times \;10^{4}$	$-1.31\;\times \;10^{4}$	$-1.32\;\times \;10^{4}$	$1.04\;\times \;10^{3}$
67	$6.71\times 10^{-91}$	$3.52\;\times \;10^{-80}$	$1.56\;\times \;10^{-86}$	$7.73\;\times \;10^{-82}$	$4.99\;\times \;10^{-81}$
68	$-4.50\times 10^{2}$	$-4.50\;\times \;10^{2}$	$-4.50\;\times \;10^{2}$	$-4.50\;\times \;10^{2}$	$2.69\;\times \;10^{-14}$
69	0.00	$8.06\;\times \;10^{-2}$	$7.40\;\times \;10^{-3}$	$1.61\;\times \;10^{-2}$	$2.03\;\times \;10^{-2}$
70	$-1.80\times 10^{2}$	$-1.80\;\times \;10^{2}$	$-1.80\;\times \;10^{2}$	$-1.80\;\times \;10^{2}$	$2.91\;\times \;10^{-2}$
71	0.00	$6.39\;\times \;10^{-2}$	$8.63\;\times \;10^{-3}$	$1.20\;\times \;10^{-2}$	$1.37\;\times \;10^{-2}$
72	$7.65\times 10^{-97}$	$1.49\;\times \;10^{-86}$	$6.08\;\times \;10^{-92}$	$3.90\;\times \;10^{-88}$	$2.16\;\times \;10^{-87}$
73	$-4.50\times 10^{2}$	$-4.50\;\times \;10^{2}$	$-4.50\;\times \;10^{2}$	$-4.50\;\times \;10^{2}$	$8.12\;\times \;10^{-15}$
74	$-2.78\times 10^{1}$	$-2.18\;\times \;10^{1}$	$-2.44\;\times \;10^{1}$	$-2.44\;\times \;10^{1}$	1.10
75	$-8.25\times 10^{-1}$	$-7.69\;\times \;10^{-1}$	$-7.85\;\times \;10^{-1}$	$-7.86\;\times \;10^{-1}$	$1.15\;\times \;10^{-2}$
76	$7.85\times 10^{-11}$	$3.29\;\times \;10^{-8}$	$1.37\;\times \;10^{-9}$	$3.59\;\times \;10^{-9}$	$6.47\;\times \;10^{-9}$
77	$5.14\times 10^{-152}$	$3.58\;\times \;10^{-132}$	$7.62\;\times \;10^{-145}$	$7.17\;\times \;10^{-134}$	$5.07\;\times \;10^{-133}$
78	−4.53	−1.62	−3.26	−3.23	$5.53\;\times \;10^{-1}$
79	$4.18\times 10^{1}$	$1.09\;\times \;10^{2}$	$7.01\;\times \;10^{1}$	$7.08\;\times \;10^{1}$	$1.50\;\times \;10^{1}$
80	$5.57\times 10^{1}$	$1.72\;\times \;10^{2}$	$9.50\;\times \;10^{1}$	$9.93\;\times \;10^{1}$	$2.95\;\times \;10^{1}$
81	$2.49\times 10^{-3}$	$1.76\;\times \;10^{2}$	$1.03\;\times \;10^{1}$	$2.23\;\times \;10^{1}$	$4.59\;\times \;10^{1}$
82	$1.06\times 10^{1}$	$9.07\;\times \;10^{3}$	$2.40\;\times \;10^{1}$	$5.56\;\times \;10^{2}$	$1.57\;\times \;10^{3}$
83	$1.00\times 10^{-1}$	$4.00\;\times \;10^{-1}$	$1.73\;\times \;10^{-1}$	$1.75\;\times \;10^{-1}$	$5.22\;\times \;10^{-2}$
84	5.31	$1.08\;\times \;10^{1}$	8.76	8.55	1.17
85	$4.55\times 10^{-11}$	$2.94\;\times \;10^{-8}$	$1.41\;\times \;10^{-9}$	$2.42\;\times \;10^{-9}$	$4.46\;\times \;10^{-9}$
86	$3.49\times 10^{-10}$	$1.61\;\times \;10^{-6}$	$9.79\;\times \;10^{-9}$	$1.24\;\times \;10^{-7}$	$3.25\;\times \;10^{-7}$
87	$3.72\times 10^{3}$	$7.66\;\times \;10^{3}$	$5.54\;\times \;10^{3}$	$5.63\;\times \;10^{3}$	$1.08\;\times \;10^{3}$
88	$3.66\times 10^{2}$	$8.10\;\times \;10^{4}$	$1.71\;\times \;10^{4}$	$2.08\;\times \;10^{4}$	$1.91\;\times \;10^{4}$
89	$1.29\times 10^{-6}$	$3.50\;\times \;10^{-3}$	$3.36\;\times \;10^{-5}$	$1.71\;\times \;10^{-4}$	$5.18\;\times \;10^{-4}$
90	$5.68\times 10^{-14}$	$1.14\;\times \;10^{-13}$	$1.14\;\times \;10^{-13}$	$9.89\;\times \;10^{-14}$	$2.52\;\times \;10^{-14}$
91	$-4.50\times 10^{2}$	$-4.36\;\times \;10^{2}$	$-4.45\;\times \;10^{2}$	$-4.44\;\times \;10^{2}$	4.00
92	$-2.12\times 10^{33}$	$-7.13\;\times \;10^{29}$	$-6.51\;\times \;10^{31}$	$-2.64\;\times \;10^{32}$	$4.63\;\times \;10^{32}$
93	0.00	0.00	0.00	0.00	0.00
94	0.00	0.00	0.00	0.00	0.00
95	1.45	$1.19\;\times \;10^{1}$	5.57	5.73	2.41

**Table A8:** Error values for LPSO.

F No.	Min	Max	Median	Mean	StdDev
2	$7.35\times 10^{-2}$	5.25	1.03	1.32	1.10
4	$3.43\times 10^{3}$	$2.74\;\times \;10^{4}$	$1.49\;\times \;10^{4}$	$1.43\;\times \;10^{4}$	$4.77\;\times \;10^{3}$
5	$4.20\times 10^{3}$	$1.17\;\times \;10^{4}$	$5.93\;\times \;10^{3}$	$6.28\;\times \;10^{3}$	$1.42\;\times \;10^{3}$
6	$6.36\times 10^{-1}$	$3.30\;\times \;10^{2}$	$2.22\;\times \;10^{1}$	$5.16\;\times \;10^{1}$	$6.70\;\times \;10^{1}$
7	$4.70\times 10^{3}$	$4.70\;\times \;10^{3}$	$4.70\;\times \;10^{3}$	$4.70\;\times \;10^{3}$	$4.00\;\times \;10^{-5}$
8	$2.07\times 10^{1}$	$2.10\;\times \;10^{1}$	$2.09\;\times \;10^{1}$	$2.09\;\times \;10^{1}$	$9.01\;\times \;10^{-2}$
11	$2.50\times 10^{1}$	$3.76\;\times \;10^{1}$	$3.09\;\times \;10^{1}$	$3.08\;\times \;10^{1}$	2.40
12	$8.61\times 10^{2}$	$3.56\;\times \;10^{4}$	$1.00\;\times \;10^{4}$	$1.11\;\times \;10^{4}$	$7.44\;\times \;10^{3}$
13	3.50	$1.03\;\times \;10^{1}$	5.92	5.98	1.50
14	$1.16\times 10^{1}$	$1.32\;\times \;10^{1}$	$1.28\;\times \;10^{1}$	$1.27\;\times \;10^{1}$	$3.35\;\times \;10^{-1}$
15	$2.27\times 10^{2}$	$5.14\;\times \;10^{2}$	$3.84\;\times \;10^{2}$	$3.70\;\times \;10^{2}$	$6.77\;\times \;10^{1}$
16	$1.18\times 10^{2}$	$5.00\;\times \;10^{2}$	$2.56\;\times \;10^{2}$	$2.74\;\times \;10^{2}$	$9.87\;\times \;10^{1}$
17	$1.55\times 10^{2}$	$5.43\;\times \;10^{2}$	$2.84\;\times \;10^{2}$	$3.11\;\times \;10^{2}$	$9.38\;\times \;10^{1}$
18	$9.12\times 10^{2}$	$1.04\;\times \;10^{3}$	$9.35\;\times \;10^{2}$	$9.40\;\times \;10^{2}$	$2.07\;\times \;10^{1}$
19	$8.00\times 10^{2}$	$1.03\;\times \;10^{3}$	$9.33\;\times \;10^{2}$	$9.36\;\times \;10^{2}$	$2.94\;\times \;10^{1}$
20	$8.00\times 10^{2}$	$9.71\;\times \;10^{2}$	$9.36\;\times \;10^{2}$	$9.35\;\times \;10^{2}$	$2.38\;\times \;10^{1}$
21	$5.00\times 10^{2}$	$1.17\;\times \;10^{3}$	$5.00\;\times \;10^{2}$	$5.72\;\times \;10^{2}$	$1.80\;\times \;10^{2}$
22	$9.69\times 10^{2}$	$1.15\;\times \;10^{3}$	$1.06\;\times \;10^{3}$	$1.06\;\times \;10^{3}$	$5.51\;\times \;10^{1}$
23	$5.00\times 10^{2}$	$1.18\;\times \;10^{3}$	$5.00\;\times \;10^{2}$	$5.70\;\times \;10^{2}$	$1.78\;\times \;10^{2}$
24	$2.00\times 10^{2}$	$2.00\;\times \;10^{2}$	$2.00\;\times \;10^{2}$	$2.00\;\times \;10^{2}$	$1.44\;\times \;10^{-12}$
25	$1.66\times 10^{3}$	$1.71\;\times \;10^{3}$	$1.68\;\times \;10^{3}$	$1.68\;\times \;10^{3}$	9.70
31	0.00	$2.27\;\times \;10^{-13}$	0.00	$2.73\;\times \;10^{-14}$	$7.46\;\times \;10^{-14}$
32	$3.36\times 10^{5}$	$7.09\;\times \;10^{6}$	$2.49\;\times \;10^{6}$	$2.54\;\times \;10^{6}$	$1.21\;\times \;10^{6}$
33	$1.75\times 10^{7}$	$4.01\;\times \;10^{9}$	$3.28\;\times \;10^{8}$	$8.00\;\times \;10^{8}$	$1.00\;\times \;10^{9}$
34	$2.25\times 10^{4}$	$9.06\;\times \;10^{4}$	$4.35\;\times \;10^{4}$	$4.46\;\times \;10^{4}$	$1.28\;\times \;10^{4}$
35	$1.14\times 10^{-13}$	$1.14\;\times \;10^{-13}$	$1.14\;\times \;10^{-13}$	$1.14\;\times \;10^{-13}$	0.00
36	$5.32\times 10^{-1}$	$7.76\;\times \;10^{1}$	$1.63\;\times \;10^{1}$	$2.38\;\times \;10^{1}$	$1.95\;\times \;10^{1}$
37	$5.70\times 10^{1}$	$1.75\;\times \;10^{2}$	$1.09\;\times \;10^{2}$	$1.11\;\times \;10^{2}$	$2.51\;\times \;10^{1}$
38	$2.08\times 10^{1}$	$2.11\;\times \;10^{1}$	$2.10\;\times \;10^{1}$	$2.10\;\times \;10^{1}$	$4.56\;\times \;10^{-2}$
39	$2.51\times 10^{1}$	$3.67\;\times \;10^{1}$	$3.21\;\times \;10^{1}$	$3.20\;\times \;10^{1}$	2.50
40	$2.46\times 10^{-2}$	$3.23\;\times \;10^{-1}$	$1.28\;\times \;10^{-1}$	$1.34\;\times \;10^{-1}$	$5.92\;\times \;10^{-2}$
41	$4.08\times 10^{1}$	$1.41\;\times \;10^{2}$	$7.01\;\times \;10^{1}$	$7.36\;\times \;10^{1}$	$1.79\;\times \;10^{1}$
42	$5.87\times 10^{1}$	$1.79\;\times \;10^{2}$	$1.12\;\times \;10^{2}$	$1.15\;\times \;10^{2}$	$3.15\;\times \;10^{1}$
43	$9.63\times 10^{1}$	$2.32\;\times \;10^{2}$	$1.59\;\times \;10^{2}$	$1.67\;\times \;10^{2}$	$3.63\;\times \;10^{1}$
44	$1.71\times 10^{3}$	$3.65\;\times \;10^{3}$	$2.87\;\times \;10^{3}$	$2.84\;\times \;10^{3}$	$4.36\;\times \;10^{2}$
45	$2.67\times 10^{3}$	$5.50\;\times \;10^{3}$	$4.19\;\times \;10^{3}$	$4.26\;\times \;10^{3}$	$6.16\;\times \;10^{2}$
46	$6.70\times 10^{-1}$	2.48	1.49	1.47	$3.48\;\times \;10^{-1}$
47	$7.86\times 10^{1}$	$1.51\;\times \;10^{2}$	$1.05\;\times \;10^{2}$	$1.08\;\times \;10^{2}$	$1.69\;\times \;10^{1}$
48	$6.57\times 10^{1}$	$1.56\;\times \;10^{2}$	$1.04\;\times \;10^{2}$	$1.06\;\times \;10^{2}$	$1.95\;\times \;10^{1}$
49	3.97	$1.20\;\times \;10^{1}$	6.86	7.22	2.00
50	$1.03\times 10^{1}$	$1.24\;\times \;10^{1}$	$1.16\;\times \;10^{1}$	$1.16\;\times \;10^{1}$	$4.57\;\times \;10^{-1}$
51	$1.00\times 10^{2}$	$4.00\;\times \;10^{2}$	$3.00\;\times \;10^{2}$	$2.59\;\times \;10^{2}$	$7.42\;\times \;10^{1}$
52	$1.81\times 10^{3}$	$4.90\;\times \;10^{3}$	$3.51\;\times \;10^{3}$	$3.49\;\times \;10^{3}$	$6.68\;\times \;10^{2}$
53	$3.72\times 10^{3}$	$7.16\;\times \;10^{3}$	$5.35\;\times \;10^{3}$	$5.39\;\times \;10^{3}$	$8.47\;\times \;10^{2}$
54	$2.46\times 10^{2}$	$3.09\;\times \;10^{2}$	$2.86\;\times \;10^{2}$	$2.86\;\times \;10^{2}$	$1.23\;\times \;10^{1}$
55	$2.96\times 10^{2}$	$3.36\;\times \;10^{2}$	$3.20\;\times \;10^{2}$	$3.20\;\times \;10^{2}$	8.24
56	$2.00\times 10^{2}$	$3.89\;\times \;10^{2}$	$2.00\;\times \;10^{2}$	$2.39\;\times \;10^{2}$	$7.31\;\times \;10^{1}$
57	$8.95\times 10^{2}$	$1.33\;\times \;10^{3}$	$1.17\;\times \;10^{3}$	$1.16\;\times \;10^{3}$	$9.78\;\times \;10^{1}$
58	$1.00\times 10^{2}$	$1.72\;\times \;10^{3}$	$3.00\;\times \;10^{2}$	$3.20\;\times \;10^{2}$	$2.06\;\times \;10^{2}$
61	$6.41\times 10^{-24}$	$3.21\;\times \;10^{-22}$	$6.81\;\times \;10^{-23}$	$9.09\;\times \;10^{-23}$	$6.77\;\times \;10^{-23}$
62	$4.00\times 10^{-15}$	$7.55\;\times \;10^{-15}$	$7.55\;\times \;10^{-15}$	$6.91\;\times \;10^{-15}$	$1.38\;\times \;10^{-15}$
63	$-1.40\times 10^{2}$	$-1.40\;\times \;10^{2}$	$-1.40\;\times \;10^{2}$	$-1.40\;\times \;10^{2}$	$1.82\;\times \;10^{-14}$
64	$4.00\times 10^{-15}$	2.96	1.34	1.11	$8.42\;\times \;10^{-1}$
65	$-1.06\times 10^{12}$	$-1.10\;\times \;10^{10}$	$-1.39\;\times \;10^{11}$	$-2.40\;\times \;10^{11}$	$2.43\;\times \;10^{11}$
66	$-1.45\times 10^{4}$	$-1.11\;\times \;10^{4}$	$-1.28\;\times \;10^{4}$	$-1.27\;\times \;10^{4}$	$8.79\;\times \;10^{2}$
67	$2.93\times 10^{-37}$	$4.48\;\times \;10^{-35}$	$4.07\;\times \;10^{-36}$	$7.06\;\times \;10^{-36}$	$8.94\;\times \;10^{-36}$
68	$-4.50\times 10^{2}$	$-4.50\;\times \;10^{2}$	$-4.50\;\times \;10^{2}$	$-4.50\;\times \;10^{2}$	$1.99\;\times \;10^{-14}$
69	0.00	$1.97\;\times \;10^{-2}$	0.00	$1.84\;\times \;10^{-3}$	$4.16\;\times \;10^{-3}$
70	$-1.80\times 10^{2}$	$-1.80\;\times \;10^{2}$	$-1.80\;\times \;10^{2}$	$-1.80\;\times \;10^{2}$	$4.71\;\times \;10^{-3}$
71	0.00	$1.48\;\times \;10^{-2}$	0.00	$2.07\;\times \;10^{-3}$	$4.12\;\times \;10^{-3}$
72	$2.37\times 10^{-42}$	$5.44\;\times \;10^{-39}$	$1.71\;\times \;10^{-40}$	$5.24\;\times \;10^{-40}$	$1.00\;\times \;10^{-39}$
73	$-4.50\times 10^{2}$	$-4.50\;\times \;10^{2}$	$-4.50\;\times \;10^{2}$	$-4.50\;\times \;10^{2}$	$8.39\;\times \;10^{-7}$
74	$-2.67\times 10^{1}$	$-2.23\;\times \;10^{1}$	$-2.46\;\times \;10^{1}$	$-2.45\;\times \;10^{1}$	$8.05\;\times \;10^{-1}$
75	$-8.12\times 10^{-1}$	$-7.85\;\times \;10^{-1}$	$-7.85\;\times \;10^{-1}$	$-7.91\;\times \;10^{-1}$	$6.97\;\times \;10^{-3}$
76	$3.41\times 10^{-2}$	8.93	$4.68\;\times \;10^{-1}$	$8.03\;\times \;10^{-1}$	1.30
77	$1.75\times 10^{-63}$	$4.40\;\times \;10^{-59}$	$1.60\;\times \;10^{-61}$	$2.34\;\times \;10^{-60}$	$6.64\;\times \;10^{-60}$
78	−4.40	−2.79	−3.46	−3.46	$3.17\;\times \;10^{-1}$
79	$4.18\times 10^{1}$	$1.10\;\times \;10^{2}$	$7.36\;\times \;10^{1}$	$7.19\;\times \;10^{1}$	$1.47\;\times \;10^{1}$
80	$5.87\times 10^{1}$	$1.36\;\times \;10^{2}$	$8.81\;\times \;10^{1}$	$9.03\;\times \;10^{1}$	$1.94\;\times \;10^{1}$
81	$2.30\times 10^{-1}$	$1.49\;\times \;10^{2}$	$2.24\;\times \;10^{1}$	$3.54\;\times \;10^{1}$	$3.79\;\times \;10^{1}$
82	$2.15\times 10^{1}$	$5.65\;\times \;10^{3}$	$2.61\;\times \;10^{1}$	$1.93\;\times \;10^{2}$	$8.06\;\times \;10^{2}$
83	$1.00\times 10^{-1}$	$1.41\;\times \;10^{-1}$	$1.00\;\times \;10^{-1}$	$1.20\;\times \;10^{-1}$	$2.09\;\times \;10^{-2}$
84	8.16	$1.13\;\times \;10^{1}$	$1.01\;\times \;10^{1}$	9.94	$7.53\;\times \;10^{-1}$
85	$9.40\times 10^{-2}$	3.27	$5.55\;\times \;10^{-1}$	$6.52\;\times \;10^{-1}$	$5.99\;\times \;10^{-1}$
86	$2.68\times 10^{-1}$	$1.98\;\times \;10^{1}$	1.30	2.43	3.33
87	$3.31\times 10^{3}$	$1.01\;\times \;10^{4}$	$6.24\;\times \;10^{3}$	$6.31\;\times \;10^{3}$	$1.41\;\times \;10^{3}$
88	$8.95\times 10^{2}$	$3.21\;\times \;10^{4}$	$9.01\;\times \;10^{3}$	$9.81\;\times \;10^{3}$	$6.99\;\times \;10^{3}$
89	$2.82\times 10^{-2}$	$4.83\;\times \;10^{-1}$	$1.29\;\times \;10^{-1}$	$1.70\;\times \;10^{-1}$	$1.22\;\times \;10^{-1}$
90	$5.68\times 10^{-14}$	$1.14\;\times \;10^{-13}$	$5.68\;\times \;10^{-14}$	$5.91\;\times \;10^{-14}$	$1.13\;\times \;10^{-14}$
91	$-4.50\times 10^{2}$	$-4.36\;\times \;10^{2}$	$-4.46\;\times \;10^{2}$	$-4.45\;\times \;10^{2}$	3.31
92	$-2.57\times 10^{31}$	$-9.33\;\times \;10^{26}$	$-1.01\;\times \;10^{29}$	$-2.83\;\times \;10^{30}$	$6.05\;\times \;10^{30}$
93	0.00	0.00	0.00	0.00	0.00
94	0.00	$2.49\;\times \;10^{-14}$	0.00	$1.28\;\times \;10^{-15}$	$3.98\;\times \;10^{-15}$
95	0.00	$2.31\;\times \;10^{-1}$	0.00	$4.62\;\times \;10^{-3}$	$3.27\;\times \;10^{-2}$

**Table A9:** Error values for DE.

F No.	Min	Max	Median	Mean	StdDev
2	$2.12\times 10^{-5}$	1.16	$1.56\;\times \;10^{-3}$	$1.91\;\times \;10^{-2}$	$8.94\;\times \;10^{-2}$
4	$4.27\times 10^{3}$	$6.27\;\times \;10^{4}$	$2.38\;\times \;10^{4}$	$2.49\;\times \;10^{4}$	$1.12\;\times \;10^{4}$
5	$2.42\times 10^{3}$	$8.98\;\times \;10^{3}$	$4.13\;\times \;10^{3}$	$4.20\;\times \;10^{3}$	$1.04\;\times \;10^{3}$
6	$7.29\times 10^{-5}$	$1.90\;\times \;10^{2}$	$1.03\;\times \;10^{1}$	$2.24\;\times \;10^{1}$	$3.15\;\times \;10^{1}$
7	$4.70\times 10^{3}$	$4.70\;\times \;10^{3}$	$4.70\;\times \;10^{3}$	$4.70\;\times \;10^{3}$	$7.73\;\times \;10^{-8}$
8	$2.09\times 10^{1}$	$2.11\;\times \;10^{1}$	$2.10\;\times \;10^{1}$	$2.10\;\times \;10^{1}$	$6.48\;\times \;10^{-2}$
11	$1.15\times 10^{1}$	$4.06\;\times \;10^{1}$	$1.73\;\times \;10^{1}$	$1.77\;\times \;10^{1}$	5.19
12	$1.58\times 10^{2}$	$9.24\;\times \;10^{4}$	$1.41\;\times \;10^{4}$	$1.84\;\times \;10^{4}$	$1.58\;\times \;10^{4}$
13	1.97	$1.12\;\times \;10^{1}$	3.82	4.21	1.96
14	$1.25\times 10^{1}$	$1.35\;\times \;10^{1}$	$1.29\;\times \;10^{1}$	$1.29\;\times \;10^{1}$	$2.77\;\times \;10^{-1}$
15	$2.04\times 10^{2}$	$5.62\;\times \;10^{2}$	$4.12\;\times \;10^{2}$	$4.05\;\times \;10^{2}$	$8.02\;\times \;10^{1}$
16	$7.74\times 10^{1}$	$5.02\;\times \;10^{2}$	$2.19\;\times \;10^{2}$	$2.87\;\times \;10^{2}$	$1.66\;\times \;10^{2}$
17	$2.25\times 10^{2}$	$1.02\;\times \;10^{3}$	$3.98\;\times \;10^{2}$	$4.39\;\times \;10^{2}$	$1.65\;\times \;10^{2}$
18	$8.00\times 10^{2}$	$1.01\;\times \;10^{3}$	$9.42\;\times \;10^{2}$	$9.48\;\times \;10^{2}$	$3.06\;\times \;10^{1}$
19	$9.22\times 10^{2}$	$9.91\;\times \;10^{2}$	$9.46\;\times \;10^{2}$	$9.48\;\times \;10^{2}$	$1.62\;\times \;10^{1}$
20	$8.00\times 10^{2}$	$1.02\;\times \;10^{3}$	$9.42\;\times \;10^{2}$	$9.42\;\times \;10^{2}$	$4.21\;\times \;10^{1}$
21	$5.00\times 10^{2}$	$1.21\;\times \;10^{3}$	$5.00\;\times \;10^{2}$	$8.00\;\times \;10^{2}$	$3.32\;\times \;10^{2}$
22	$9.60\times 10^{2}$	$1.19\;\times \;10^{3}$	$1.02\;\times \;10^{3}$	$1.03\;\times \;10^{3}$	$4.88\;\times \;10^{1}$
23	$5.00\times 10^{2}$	$1.19\;\times \;10^{3}$	$5.00\;\times \;10^{2}$	$7.68\;\times \;10^{2}$	$3.16\;\times \;10^{2}$
24	$2.00\times 10^{2}$	$1.28\;\times \;10^{3}$	$2.55\;\times \;10^{2}$	$6.21\;\times \;10^{2}$	$4.78\;\times \;10^{2}$
25	$1.67\times 10^{3}$	$1.76\;\times \;10^{3}$	$1.70\;\times \;10^{3}$	$1.70\;\times \;10^{3}$	$1.92\;\times \;10^{1}$
31	0.00	0.00	0.00	0.00	0.00
32	$7.67\times 10^{5}$	$1.10\;\times \;10^{7}$	$3.78\;\times \;10^{6}$	$4.04\;\times \;10^{6}$	$2.29\;\times \;10^{6}$
33	$4.51\times 10^{6}$	$6.89\;\times \;10^{9}$	$2.74\;\times \;10^{8}$	$5.94\;\times \;10^{8}$	$1.10\;\times \;10^{9}$
34	$6.67\times 10^{3}$	$3.77\;\times \;10^{4}$	$2.11\;\times \;10^{4}$	$2.08\;\times \;10^{4}$	$7.05\;\times \;10^{3}$
35	0.00	$1.14\;\times \;10^{-13}$	$1.14\;\times \;10^{-13}$	$7.50\;\times \;10^{-14}$	$5.44\;\times \;10^{-14}$
36	7.21	$8.02\;\times \;10^{1}$	$4.11\;\times \;10^{1}$	$4.70\;\times \;10^{1}$	$2.70\;\times \;10^{1}$
37	$1.87\times 10^{1}$	$1.77\;\times \;10^{2}$	$8.83\;\times \;10^{1}$	$9.25\;\times \;10^{1}$	$3.26\;\times \;10^{1}$
38	$2.08\times 10^{1}$	$2.11\;\times \;10^{1}$	$2.10\;\times \;10^{1}$	$2.10\;\times \;10^{1}$	$6.33\;\times \;10^{-2}$
39	$1.37\times 10^{1}$	$2.46\;\times \;10^{1}$	$1.91\;\times \;10^{1}$	$1.92\;\times \;10^{1}$	2.91
40	$4.20\times 10^{-2}$	$1.35\;\times \;10^{1}$	1.44	1.64	2.02
41	$3.98\times 10^{1}$	$1.69\;\times \;10^{2}$	$8.86\;\times \;10^{1}$	$9.01\;\times \;10^{1}$	$2.90\;\times \;10^{1}$
42	$4.18\times 10^{1}$	$2.29\;\times \;10^{2}$	$1.17\;\times \;10^{2}$	$1.22\;\times \;10^{2}$	$5.00\;\times \;10^{1}$
43	$1.12\times 10^{2}$	$2.91\;\times \;10^{2}$	$1.80\;\times \;10^{2}$	$1.82\;\times \;10^{2}$	$3.86\;\times \;10^{1}$
44	$5.55\times 10^{2}$	$2.06\;\times \;10^{3}$	$1.12\;\times \;10^{3}$	$1.13\;\times \;10^{3}$	$3.18\;\times \;10^{2}$
45	$6.46\times 10^{3}$	$8.34\;\times \;10^{3}$	$7.55\;\times \;10^{3}$	$7.48\;\times \;10^{3}$	$3.65\;\times \;10^{2}$
46	1.99	3.47	2.95	2.90	$3.19\;\times \;10^{-1}$
47	$5.75\times 10^{1}$	$1.79\;\times \;10^{2}$	$9.62\;\times \;10^{1}$	$9.90\;\times \;10^{1}$	$2.59\;\times \;10^{1}$
48	$7.79\times 10^{1}$	$2.27\;\times \;10^{2}$	$1.24\;\times \;10^{2}$	$1.31\;\times \;10^{2}$	$3.74\;\times \;10^{1}$
49	4.35	$9.32\;\times \;10^{1}$	$1.97\;\times \;10^{1}$	$2.56\;\times \;10^{1}$	$1.86\;\times \;10^{1}$
50	$1.10\times 10^{1}$	$1.27\;\times \;10^{1}$	$1.19\;\times \;10^{1}$	$1.19\;\times \;10^{1}$	$4.14\;\times \;10^{-1}$
51	$1.00\times 10^{2}$	$4.00\;\times \;10^{2}$	$3.00\;\times \;10^{2}$	$3.04\;\times \;10^{2}$	$7.55\;\times \;10^{1}$
52	$3.69\times 10^{2}$	$2.13\;\times \;10^{3}$	$1.23\;\times \;10^{3}$	$1.24\;\times \;10^{3}$	$4.74\;\times \;10^{2}$
53	$6.41\times 10^{3}$	$8.64\;\times \;10^{3}$	$7.64\;\times \;10^{3}$	$7.56\;\times \;10^{3}$	$5.01\;\times \;10^{2}$
54	$2.33\times 10^{2}$	$2.84\;\times \;10^{2}$	$2.60\;\times \;10^{2}$	$2.59\;\times \;10^{2}$	$1.24\;\times \;10^{1}$
55	$2.59\times 10^{2}$	$3.03\;\times \;10^{2}$	$2.86\;\times \;10^{2}$	$2.86\;\times \;10^{2}$	8.55
56	$2.00\times 10^{2}$	$3.77\;\times \;10^{2}$	$3.44\;\times \;10^{2}$	$3.18\;\times \;10^{2}$	$6.02\;\times \;10^{1}$
57	$5.83\times 10^{2}$	$1.08\;\times \;10^{3}$	$8.51\;\times \;10^{2}$	$8.35\;\times \;10^{2}$	$9.21\;\times \;10^{1}$
58	$1.00\times 10^{2}$	$1.84\;\times \;10^{3}$	$6.85\;\times \;10^{2}$	$7.45\;\times \;10^{2}$	$5.12\;\times \;10^{2}$
61	$6.90\times 10^{-42}$	$1.50\;\times \;10^{-13}$	$1.06\;\times \;10^{-34}$	$3.01\;\times \;10^{-15}$	$2.13\;\times \;10^{-14}$
62	1.34	9.66	3.49	3.94	1.78
63	$-1.39\times 10^{2}$	$-1.31\;\times \;10^{2}$	$-1.36\;\times \;10^{2}$	$-1.36\;\times \;10^{2}$	2.05
64	$4.00\times 10^{-15}$	6.96	3.43	3.50	1.35
65	$-1.38\times 10^{12}$	$-5.00\;\times \;10^{10}$	$-2.99\;\times \;10^{11}$	$-3.61\;\times \;10^{11}$	$2.89\;\times \;10^{11}$
66	$-1.84\times 10^{4}$	$-1.26\;\times \;10^{4}$	$-1.55\;\times \;10^{4}$	$-1.53\;\times \;10^{4}$	$1.38\;\times \;10^{3}$
67	$4.22\times 10^{-97}$	$2.08\;\times \;10^{-85}$	$3.43\;\times \;10^{-91}$	$7.40\;\times \;10^{-87}$	$3.55\;\times \;10^{-86}$
68	$-4.50\times 10^{2}$	$-4.50\;\times \;10^{2}$	$-4.50\;\times \;10^{2}$	$-4.50\;\times \;10^{2}$	0.00
69	0.00	$3.04\;\times \;10^{-1}$	$3.32\;\times \;10^{-2}$	$5.64\;\times \;10^{-2}$	$6.23\;\times \;10^{-2}$
70	$-1.80\times 10^{2}$	$-1.79\;\times \;10^{2}$	$-1.80\;\times \;10^{2}$	$-1.80\;\times \;10^{2}$	$1.30\;\times \;10^{-1}$
71	0.00	$6.12\;\times \;10^{-2}$	$2.40\;\times \;10^{-9}$	$8.61\;\times \;10^{-3}$	$1.23\;\times \;10^{-2}$
72	$1.67\times 10^{-103}$	$6.50\;\times \;10^{-90}$	$3.00\;\times \;10^{-97}$	$1.34\;\times \;10^{-91}$	$9.19\;\times \;10^{-91}$
73	$-4.50\times 10^{2}$	$-4.49\;\times \;10^{2}$	$-4.50\;\times \;10^{2}$	$-4.50\;\times \;10^{2}$	$1.04\;\times \;10^{-1}$
74	$-2.80\times 10^{1}$	$-2.25\;\times \;10^{1}$	$-2.66\;\times \;10^{1}$	$-2.64\;\times \;10^{1}$	1.13
75	$-7.85\times 10^{-1}$	$-7.51\;\times \;10^{-1}$	$-7.69\;\times \;10^{-1}$	$-7.76\;\times \;10^{-1}$	$8.61\;\times \;10^{-3}$
76	$1.30\times 10^{-5}$	1.01	$3.84\;\times \;10^{-4}$	$4.70\;\times \;10^{-2}$	$1.83\;\times \;10^{-1}$
77	$7.50\times 10^{-133}$	$6.88\;\times \;10^{-120}$	$7.06\;\times \;10^{-128}$	$1.41\;\times \;10^{-121}$	$9.73\;\times \;10^{-121}$
78	−3.63	1.27	−2.06	−1.78	1.08
79	$2.09\times 10^{1}$	$7.66\;\times \;10^{1}$	$3.83\;\times \;10^{1}$	$3.96\;\times \;10^{1}$	$1.12\;\times \;10^{1}$
80	$3.18\times 10^{1}$	$2.19\;\times \;10^{2}$	$1.09\;\times \;10^{2}$	$1.15\;\times \;10^{2}$	$6.10\;\times \;10^{1}$
81	$1.83\times 10^{-3}$	$3.38\;\times \;10^{2}$	7.17	$2.27\;\times \;10^{1}$	$5.09\;\times \;10^{1}$
82	$1.87\times 10^{1}$	$1.13\;\times \;10^{4}$	$1.61\;\times \;10^{2}$	$1.23\;\times \;10^{3}$	$2.81\;\times \;10^{3}$
83	$1.41\times 10^{-1}$	$6.71\;\times \;10^{-1}$	$1.87\;\times \;10^{-1}$	$2.32\;\times \;10^{-1}$	$1.03\;\times \;10^{-1}$
84	5.43	9.79	8.56	8.37	$8.78\;\times \;10^{-1}$
85	$3.15\times 10^{-5}$	$5.20\;\times \;10^{-1}$	$1.25\;\times \;10^{-3}$	$2.56\;\times \;10^{-2}$	$9.67\;\times \;10^{-2}$
86	$5.54\times 10^{-4}$	$1.23\;\times \;10^{1}$	$2.93\;\times \;10^{-1}$	1.11	2.19
87	$2.59\times 10^{3}$	$6.29\;\times \;10^{3}$	$4.34\;\times \;10^{3}$	$4.39\;\times \;10^{3}$	$9.33\;\times \;10^{2}$
88	$1.33\times 10^{3}$	$5.75\;\times \;10^{4}$	$1.56\;\times \;10^{4}$	$1.68\;\times \;10^{4}$	$1.35\;\times \;10^{4}$
89	$1.76\times 10^{1}$	$4.06\;\times \;10^{1}$	$2.99\;\times \;10^{1}$	$2.94\;\times \;10^{1}$	5.93
90	$5.68\times 10^{-14}$	$2.15\;\times \;10^{-8}$	$5.68\;\times \;10^{-14}$	$4.31\;\times \;10^{-10}$	$3.04\;\times \;10^{-9}$
91	$-4.49\times 10^{2}$	$-4.35\;\times \;10^{2}$	$-4.44\;\times \;10^{2}$	$-4.44\;\times \;10^{2}$	3.27
92	$-3.87\times 10^{34}$	$-6.10\;\times \;10^{32}$	$-9.52\;\times \;10^{33}$	$-1.08\;\times \;10^{34}$	$8.92\;\times \;10^{33}$
93	0.00	0.00	0.00	0.00	0.00
94	0.00	0.00	0.00	0.00	0.00
95	$8.51\times 10^{-1}$	9.83	3.81	4.08	1.90
